# Altering glycopeptide antibiotic biosynthesis through mutasynthesis allows incorporation of fluorinated phenylglycine residues†

**DOI:** 10.1039/d4cb00140k

**Published:** 2024-08-12

**Authors:** Irina Voitsekhovskaia, Y. T. Candace Ho, Christoph Klatt, Anna Müller, Daniel L. Machell, Yi Jiun Tan, Maxine Triesman, Mara Bingel, Ralf B. Schittenhelm, Julien Tailhades, Andreas Kulik, Martin E. Maier, Gottfried Otting, Wolfgang Wohlleben, Tanja Schneider, Max Cryle, Evi Stegmann

**Affiliations:** a Microbial Bioactive Compounds, Interfaculty Institute of Microbiology and Infection Medicine Tübingen, University of Tübingen Tübingen Germany evi.stegmann@uni-tuebingen.de; b Department of Biochemistry and Molecular Biology, The Monash Biomedicine Discovery Institute, Monash University Clayton VIC 3800 Australia max.cryle@monash.edu; c EMBL Australia, Monash University Clayton VIC 3800 Australia; d ARC Centre of Excellence for Innovations in Peptide and Protein Science Australia; e Institute of Organic Chemistry, University of Tübingen Tübingen Germany; f Institute for Pharmaceutical Microbiology, University Hospital Bonn, University of Bonn Bonn Germany; g Research School of Chemistry, The Australian National University Acton ACT 2601 Australia; h Monash Proteomics and Metabolomics Platform, Monash University Clayton VIC 3800 Australia; i Microbiology/Biotechnology, Interfaculty Institute of Microbiology and Infection Medicine Tübingen, University of Tübingen Tübingen Germany; j Institute for Pharmaceutical Microbiology, University Hospital Bonn, University of Bonn Bonn Germany; k German Centre for Infection Research (DZIF), Partner Site Tübingen Tübingen Germany; l Cluster of Excellence ‘Controlling Microbes to Fight Infections’ (CMFI), University of Tübingen Tübingen Germany

## Abstract

Glycopeptide antibiotics (GPAs) are peptide natural products used as last resort treatments for antibiotic resistant bacterial infections. They are produced by the sequential activities of a linear nonribosomal peptide synthetase (NRPS), which assembles the heptapeptide core of GPAs, and cytochrome P450 (Oxy) enzymes, which perform a cascade of cyclisation reactions. The GPAs contain proteinogenic and nonproteinogenic amino acids, including phenylglycine residues such as 4-hydroxyphenylglycine (Hpg). The ability to incorporate non-proteinogenic amino acids in such peptides is a distinctive feature of the modular architecture of NRPSs, with each module selecting and incorporating a desired amino acid. Here, we have exploited this ability to produce and characterise GPA derivatives containing fluorinated phenylglycine (F-Phg) residues through a combination of mutasynthesis, biochemical, structural and bioactivity assays. Our data indicate that the incorporation of F-Phg residues is limited by poor acceptance by the NRPS machinery, and that the phenol moiety normally present on Hpg residues is essential to ensure both acceptance by the NRPS and the sequential cyclisation activity of Oxy enzymes. The principles learnt here may prove useful for the future production of GPA derivatives with more favourable properties through mixed feeding mutasynthesis approaches.

## Abstract 2

Novel, fluorinated glycopeptide antibiotics (GPAs) have been generated using mutasynthesis approaches. This also expands our knowledge of the tolerance of modified phenylglycine amino acid residues by the GPA biosynthetic assembly machinery.

## Graphical abstract

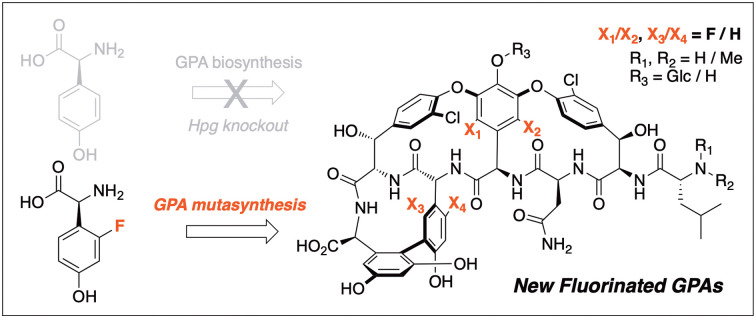

## Introduction

The glycopeptide antibiotics (GPAs) are nonribosomal peptide natural products with antibiotic activity towards Gram-positive bacteria.^1^ The clinical application of two natural (vancomycin, teicoplanin) as well as second generation semi-synthetic derivatives (dalbavancin, oritavancin) for the treatment of resistant bacterial infections,^2^ together with the application of other GPAs as analytical tools (ristomycin),^3^ highlights the importance of this class of molecules. Despite impressive efforts in total synthesis,^4–7^ the production of GPAs remains restricted to *in vitro* biosynthesis in producer strains due to the rigid, three-dimensional structure of these molecules. The rigid structure of GPAs is required for binding to the d-alanyl-d-alanine (d-Ala-d-Ala) termini of lipid II and sequestering this bacterial cell wall precursor.^8^ The requirement for GPA production in bacterial strains makes both the study and subsequent manipulation of GPA biosynthesis of key importance to the generation of modified GPAs.^9,10^ In this context, knowledge of the individual biosynthetic steps involved is essential. The biosynthesis of vancomycin-type GPAs has been extensively studied in *Amycolatopsis balhimycina*, the producer of balhimycin, which differs from vancomycin only in the pendant sugar moieties.^10^ This model system has proved invaluable in developing our understanding of GPA biosynthesis, as *A. balhimycina* was initially the only GPA producer that was genetically tractable.^10^

**Fig. 1:**
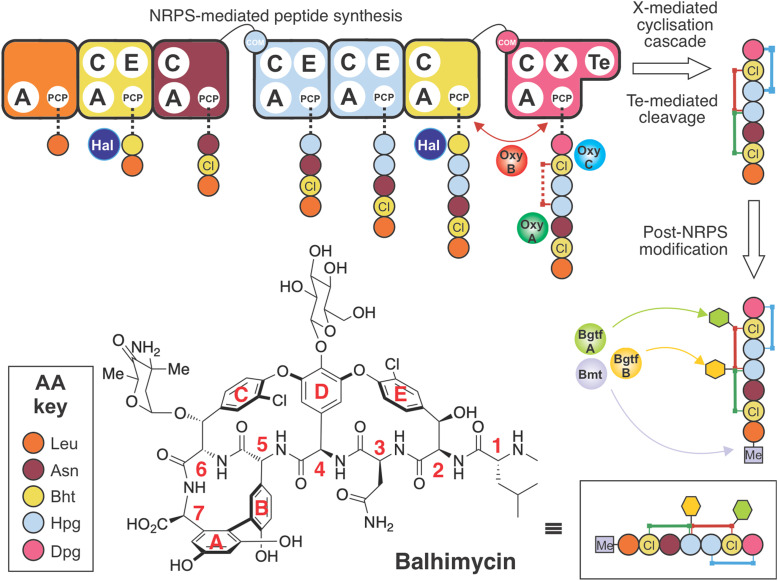
Schematic representation of balhimycin biosynthesis. The initial linear peptide is synthesised by the action of 7-module nonribosomal peptide synthetases (NRPS), with the β-hydroxytyrosine (Bht) residues at modules 2 and 6 also halogenated on the NRPS prior to peptide formation. The complete heptapeptide, when bound to module 7, then becomes the target for the P450 monooxygenase (Oxy) enzyme-mediated crosslinking cascade, with the Oxy enzymes recruited to this module (and hence the peptidyl carrier protein (PCP)-bound peptide substrate) by the neighbouring X-domain. Following crosslinking, the final thioesterase (Te) domain cleaves the crosslinked peptide from the NRPS, where it undergoes two glycosylation (*via* the glycosyltransferases BgtfA and BgtfB) and one methylation events (catalysed by Bmt) to produce the major component of balhimycin. Schematic representation of balhimycin shown in the box. A: adenylation domain; C: condensation domain; COM: communication domain; E: epimerisation domain; PCP: peptidyl carrier protein domain; X: P450 recruitment domain; Te: thioesterase domain; Hal: halogenase; Oxy: Cytochrome P450; AA: amino acid; Asn: asparagine; Bht: β-hydroxytyrosine; Dpg: 3,5-dihydroxyphenylglycine; Leu: leucine; Hpg: 4-hydroxyphenylglycine. Red letters indicate amino acid numbers, and red letters indicate aromatic rings involved in crosslinks.

Lipid II binding GPAs are produced through the combined and sequential actions of a linear nonribosomal peptide synthetase (NRPS),^11^ which assembles the heptapeptide core of GPAs, and cytochrome P450 (Oxy) enzymes, which perform a cascade of cyclisation reactions whilst the peptide remains bound to the NRPS.^12–14^ The NRPS comprises 7 modules,^9,10^ and each module contains the enzymatic domains required for the insertion of one amino acid residue into the growing peptide chain.^11^ Crucial for the specificity of each NRPS module is the activity of an adenylation (A) domain,^15,16^ which both selects the desired amino acid and catalyses its subsequent activation with the consumption of ATP.^17^ Unlike ribosomal biosynthesis, A-domains allow the incorporation of a wide (>300) range of non-proteinogenic amino acids into nonribosomal peptides (NRPs).^18^ In GPA biosynthesis (Fig. 1), this is particularly important due to the high proportion of phenylglycine residues, including both 4-hydroxyphenylglycine (Hpg) and 3,5-dihydroxyphenylglycine (Dpg), found in vancomycin-type GPAs (2 Hpg, 1 Dpg) and in teicoplanin-type GPAs (3 Hpg, 2 Dpg). Recent structural studies have revealed the unusual nature of the substrate selection pocket of the phenylglycine A-domain, which in turn highlights the rigid and linear nature of these unusual amino acids.^19^ Both Hpg^20–23^ and Dpg^24–27^ residues must be produced by dedicated biosynthetic pathways prior to their activation by the NRPS, a process that has been extensively investigated for GPAs in particular, as well as NRPs more broadly.^28^

**Fig. 2:**
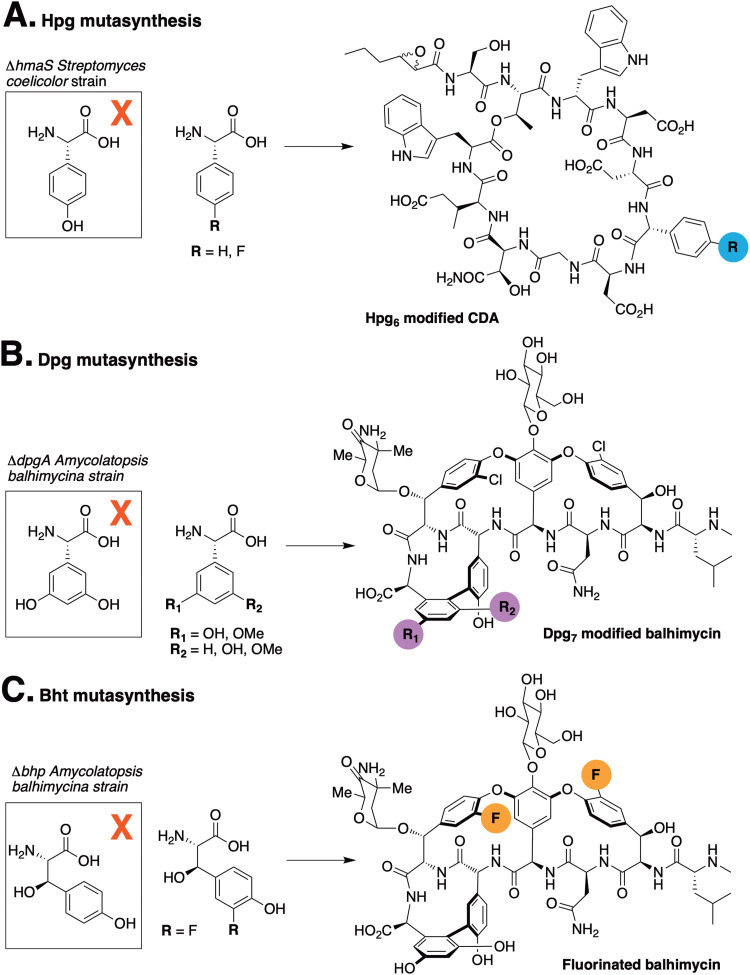
Examples of mutasynthesis campaigns that have targeted the replacement of the non-proteinogenic amino acids 4-hydroxyphenylglycine (Hpg) in CDA biosynthesis (A) and 3,5-dihydroxyphenylglycine (Dpg) and β-hydroxytyrosine (Bht) in balhimycin biosynthesis (B) and (C).

The incorporation of phenylglycine (Phg) residues into NRPS biosynthesis pathways offers the possibility to manipulate the structure of the GPAs using mutasynthesis approaches. Deletion of the biosynthesis pathways that produce such residues prevents the production of the natural product. By supplementing the mutasynthesis strain with structurally related, unnatural amino acids (mutasynthons), it is possible to produce NRP derivatives. Mutasynthesis has been successfully implemented for phenylglycine (in pristinamycin biosynthesis),^29^ Hpg (in calcium-dependent antibiotic (CDA) biosynthesis)^30^ (Fig. 2(A)) and Dpg (in GPA biosynthesis),^31^ (Fig. 2(B) and (C)) which resulted in the isolation of modified natural products in all cases. Such techniques thus offer the possibility to produce novel NRPs in a manner that can be scaled, whilst also introducing potentially valuable modifications into such peptides. In this regard, the incorporation of fluorine^32^ – which is limited in natural biosynthesis pathways^33^ but found in a significant fraction of pharmaceutical compounds due to its favourable properties^34,35^ – exemplifies the potential power of mutasynthesis to produce novel NRPs.^36^

Given both the importance of GPAs in medicine and the limited experiments probing the incorporation of fluorine (F) atoms into these molecules (the sole exception being the replacement of chlorine (Cl) with F atoms in fluorobalhimycin^37^*via* mutasynthesis of β-hydroxytyrosine, Bht), here we have developed a strategy for the incorporation of fluorine atoms within Hpg residues during GPA biosynthesis. These Hpg residues form the core of GPAs and are essential for GPA activity after they have been crosslinked by the Oxy enzyme cascade. This cascade functions in a defined order, with OxyB first installing the C-O-D crosslink between residues 4 and 6, followed by OxyA installing the D-O-E crosslink between residues 2 and 4, and finally OxyC installing the AB crosslink between residues 5 and 7 (OxyE installs the additional F-O-G crosslinking between residues 1 and 3 in teicoplanin type GPAs after the actions of OxyB).^14,38–44^ Here, we demonstrate the importance of maintaining key functional groups that allow the successful biosynthesis of both the linear peptide by the NRPS and the subsequent cyclisation by the Oxy enzymes. In doing so, we have produced novel fluorinated GPAs that are fully crosslinked and retain all other modifications including methylation, chlorination and glycosylation that are typically required to produce an active GPA. Characterisation of one of these fluorinated GPAs has further revealed the impact that these F-atoms have on the structure and hence activity of the molecule, highlighting the major impact that such relatively small changes in structure can have on the activity of these crucial antibiotics.

## Materials and methods

### 1. Construction of *A. balhimycina* Δ*hpg* strain

#### Bacterial strains and plasmids

The bacterial strains and plasmids used in this study are listed in ESI† (Table S1). For routine cloning work, *Escherichia coli* Novablue (Novagen) (Sigma-Aldrich, Germany) was used. To obtain unmethylated DNA for direct transformation, the methylation-deficient strain *E. coli* ET12567^45^ was used. *A. balhimycina* DSM5908 was used for balhimycin production and to generate the *A. balhimycina* DSM5908 Δ*hpg* mutasynthesis strain.

#### Media and cultivation conditions

*A. balhimycina* strains were grown in 10 mL R5 medium^46^ at 29 °C with 120 rpm shaking rate for balhimycin production analysis and their genomic DNA isolation. Erythromycin (50 μg mL^−1^) was used for mutants selection, when appropriate. *E. coli* strains were grown in Luria-Bertani (LB) broth. Ampicillin antibiotic (150 μg mL^−1^) was used as a selection marker for plasmids in gene cloning routine. *Staphylococcus aureus* SG511 and *Micrococcus luteus* (Schroeter) Cohn (ATCC 4698) were grown on Mueller Hinton agar and in broth (MHA and MHB) at 37 °C. *Bacillus subtilis* W168 sacA::pCHlux101 (P_liaI_-*lux*)^47^ was grown in MHB supplemented with chloramphenicol (5 μg mL^−1^) with shaking at 30 °C. *Bacullis subtilis* ATCC 6633 was grown in liquid TSB medium with shaking at 37 °C and spore suspension was prepared using Sporulation Bacillus Medium (SBM). *Lactobacillus plantarum* WJL was grown in MRS liquid medium without shaking at 37 °C and on MRS agar plates at 37 °C.

#### Preparation and manipulation of DNA

Total DNA isolation from *A. balhimycina* strains was performed as described by Kieser^46^ and with the DNA Nucleospin Microbial DNA kit (Bioanalysis, Macherey-Nagel, Germany), respectively. For plasmid isolation the peqGold Plasmid MiniPrep kit (VWR, Life Science, USA), Pure Yield Plasmid MidiPrep System kit (Promega, USA) was used. PCR products were purified from 1% agarose gel with the Wizard® SV Gel and PCR Clean-Up System kit (Promega, USA). Enzymes, including restriction endonucleases, ligase, and Q5 DNA polymerase were used according to manufacturer's recommendations (New England Biolabs, USA; Thermo Fischer Scientific, USA).

#### Strain manipulation

Primers used for PCR were obtained from MWG Biotech AG (MWG; Ebersberg, Germany) and are listed in ESI† (Table S1).

*A. balhimycina* Δ*hpg* was generated as following: for deletion of the *hmaS* and *hmO* genes, the fragment *hpg-inactFr1* (1.5 kb) and the fragment *hpg-inactFr2* (1.5 kb) were amplified by PCR with primer pairs hpg-inactFr1fw/rev and hpg-inactFr2fw/rev, respectively. PCR fragments were cloned into a pJET1.2/blunt cloning vector (Thermo Fischer Scientific, Waltham, USA), resulting in the plasmids pJET-hpg-inactFr1 and pJET-hpg-pJET-hpg-inactFr2, respectively. The *hpg-inactFr1* fragment was digested with XbaI and PstI and ligated into the vector pSP1, which was digested with the same enzymes, to give pSP1-hpg-inactFr1. The *hpg-inactFr2* fragment was digested with EcoRI and XbaI and ligated into the EcoRI-XbaI-digested pSP1-hpg-inactFr1 vector, resulting in pSP1-hpg-inactFr1 + Fr2 (ESI,† Fig. S1). The correctness of pSP1-hpg-inactFr1 + Fr2 was verified by sequencing. For transformation of *A. balhimycina*, the direct transformation method was used as reported previously.^48^ The selection of transformants containing the integrated pSP1-hpg-inactFr1 + Fr2 (after the single cross over event) was performed with erythromycin supplementation (50 μg mL^−1^) in solid and liquid media since the pSP1 vector harbors the erythromycin resistance cassette (*eryR*). To confirm the correctness of transformants harbouring pSP1-hpg-inactFr1 + Fr2, PCR analysis was performed by using the primer pairs (Ery-fw/rev), resulting in the amplification of a 0.9 kb fragment of the *eryR* cassette (ESI,† Fig. S2). For the clones with *eryR*-specific amplificants, the “stress” treatment according to Puk *et al.*^49^ was applied to increase the probability of a second cross-over event, which should result in the loss of the plasmid and the deletion of *hmaS* and *hmO*. To verify the deletion of *hmaS* and *hmO*, a PCR analysis was carried out by using the primer pair hpg-inact-hpg-fw-check/rev-check (ESI,† Fig. S3). The confirmed mutant containing the deletion of the *hmaS* and *hmO* genes was named *A. balhimycina* Δ*hpg*.

### 2. Chemical synthesis

#### Peptide synthesis

Solid phase peptide synthesis of the heptapeptidyl-CoAs 4–8 was performed using optimised conditions as previously reported.^50–52^ Peptides containing 2-F-Phg were prepared as a mixture of diastereomers due to the racemic nature of this amino acid synthon.

#### Synthesis of 2-F-4-Hpg (3)

**Fig. 3:**
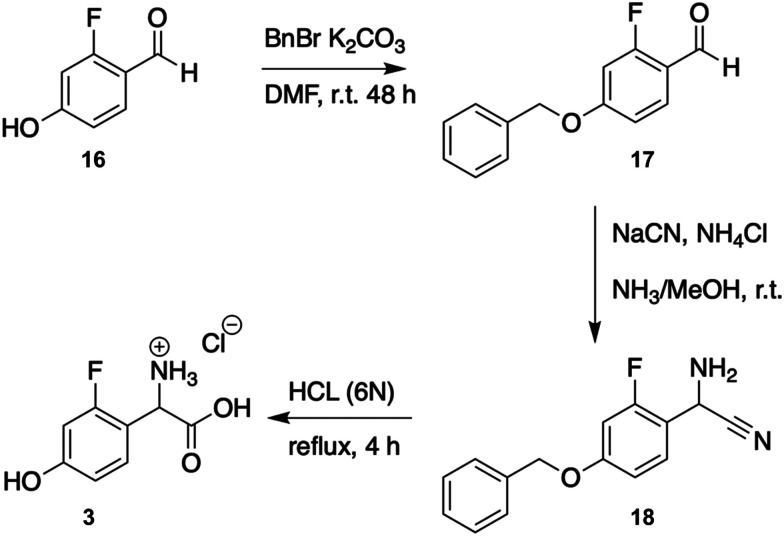
Synthesis of amino acid 3 from 2-fluoro-4-hydroxybenzaldehyde according to the literature.

Racemic F-phenylglycine 4 was synthesised from 2-fluoro-4-hydroxybenzaldehyde 16, (Fig. 3).^53^ First, the hydroxyl group of 16 was protected as benzyl ether to give 4-benzyloxyo-2-fluorobenzaldehyde (17) in 97% yield.^54^ A subsequent Strecker reaction of 17 with sodium cyanide in ammonia gave the α-aminonitrile 18 in 92% yield.^55^ Upon hydrolysis of 18 with hydrochloric acid (6 N) the desired 2-amino-2-(2-fluoro-4-hydroxyphenyl)acetic acid (3) was obtained as hydrochloride salt (94% yield) (ESI,† Protocol S1).

### 3. Mutasynthesis

#### Strain supplementation

**Fig. 4:**
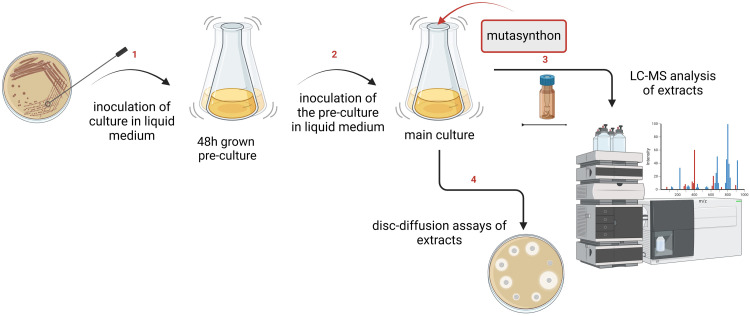
Schematic workflow for supplementation experiments. (1) Strains were inoculated into a liquid production medium and incubated as precultures for 48 hours; (2) 1 mL of the precultures were transferred to the main cultures where the bacteria were further cultured for the mutasynthesis approach. In this step, the main cultures were supplemented with unnatural amino acids, which serve as mutasynthons. (3) After cultivation, culture broth and extracts were prepared and used for HPLC-MS analysis to identify and characterise the compounds produced. (4) A disc-diffusion assay against *Bacillus subtilis* and *Lactococcus plantarum* was performed. The presence of inhibition zones around the discs indicated the potential antimicrobial activity of the produced compounds.

*A. balhimycina* strains were grown in 10 mL R5 medium as a preculture. After 48 hours, 1 mL of the preculture was used to inoculate 8 mL of fresh R5 medium as the main culture. After 24 hours, feeding experiments with *A. balhimycina* Δ*hpg* were performed with different mutasynthons (4-F-phenylglycine (4-F-Phg) (1); 2-F-phenylglycine (2-F-Phg) (2); 2-F-4-hydroxyphenylglycine (2-F-Hpg) (33)) (solubilised in 700 μL 1 N NaOH and neutralised with 500 μL 1 N HCl mg^−1^ compound to a final concentration of 1–2 mg mL^−1^ dissolved in 1 or 1.2 mL NaOH–HCl. Cultures were incubated for 7 days at 29 °C with shaking at 120 rpm (Fig. 4). As a control, *A. balhimycina* Δ*hpg* was fed with Hpg (dissolved in 1 mL R5 medium), which demonstrated the restoration of balhimycin production.

#### Compound extraction and purification

After centrifugation of the cultures (10 mL) at 14 000 rpm for 10 min, the amount of supernatant was reduced *in vacuo* to 1 mL. Additionally, 10 mL of culture filtrates were extracted with ethyl acetate and 1-butanol with a ratio 1 : 1 for 1 h at room temperature (RT) in an overhead shaker. After centrifugation at 5000 rpm for 10 min, the organic phase (ethyl acetate, 1-butanol) was dried *in vacuo* and then dissolved in 200 μL of methanol. The samples were analysed *via* HPLC-MS. For a large-scale cultivation *A. balhimycina* Δ*hpg* was cultivated in 50 × 200 mL R5 medium supplemented with 2-F-4-hydroxyphenylglycine (2-F-Hpg) (6 mg per 200 mL culture). Extraction and purification were performed as described in the ESI† (Protocol S2).

### 4. Compound characterisation

#### HPLC-ESI-MS (HPLC-ESI-MS^2^) and HPLC-HRMS of balhimycin variants

For the detection of balhimycin derivatives, 2.5–10 μL of the concentrated supernatant samples and extracts were injected and analysed by using HPLC-ESI-MS and HPLC-ESI-MS^2^ instrument (XCT 6330 HPLC/MSD Ultra Trap system; Agilent Technologies, Germany) and a Nucleosil 100 C18 column (3 μm, 100 mm by 2 mm internal diameter) fitted with a precolumn (same stationary phase, 10 by 2 mm) (Dr Maisch GmbH, Ammerbuch-Entringen, Germany). Detection of *m*/*z* values (200–2000) was conducted with Agilent DataAnalysis for 6300 series Ion Trap HPLC/MS 6.1 version 3.4 software (Bruker-Daltonics GmbH). The MS parameters were registered as follows: ionization alternating between positive and negative, the capillary voltage was 3.5 kV, and the temperature 350 °C. Tandem MS (MS^2^) was performed in the positive mode with the corresponding target masses. The HPLC parameters were as follows: solvent A was 0.1% formic acid in water, and solvent B was 0.06% formic acid in acetonitrile. The elution was performed by a linear gradient from 0–100% over 15 min of solvent B against solvent A with the flow rate of 0.4 mL min^−1^. UV spectroscopic data were collected by a DAD detector in the range of 230–600 nm.

High-resolution mass spectroscopic data (HRMS) with HPLC were acquired on HR-ESI/APCI-TOF (maXis 4G, Bruker) system with the mass range 50–1800 *m*/*z* in positive ion mode coupled with HPLC UltiMate 3000 system (Thermo Fischer Scientific), using a Luna Omega polar C18, 150 × 4.6 mm, 3 μm dp, 100 Å HPLC column (Phenomenex, Madrid Ave, Torrance, CA, USA). A linear gradient from 5–100% with solvent B (distilled acetonitrile + 0.1% formic acid) against solvent A (bi-distilled water + 0.1% formic acid) starting with 5% of Solvent B for 5 min and then from 5% to 100% with solvent B for 25 min and holding with 100% of solvent B for 10 min at a flow rate of 300 μL min^−1^ and 30 °C column temperature was used to separate 1–5 μL sample. UV spectroscopic data were collected by a DAD detector in the range of 200–600 nm.

#### HRMS analysis of peptide turnovers

Samples were separated on a RSLC 3000 HPLC system (Thermo) coupled to an Orbitrap Fusion Tribrid mass spectrometer (Thermo Scientific). The HPLC system consisted of a trap column Acclaim PepMap 100 (100 μm × 2 cm, nanoViper, C18, 5 μm, 100 Å; Thermo Scientific) and an Acclaim PepMap RSLC analytical column (75 μm × 50 cm, nanoViper, C18, 2 μm, 100 Å; Thermo Scientific). Samples were loaded onto the trap column in μL-pickup mode using 2% acetonitrile, 0.1% TFA loading buffer. Using a 30 min gradient and a flow rate of 250 nL min^−1^, compounds were eluted from the column by increasing concentrations of buffer B (80% acetonitrile, 0.1% formic acid (FA); ranging from 6% to 30%) and ionised in the nanospray source operated at 1.7 kV. The mass spectrometer was operated in both data-dependent acquisition (DDA) and parallel reaction monitoring (PRM) mode to target the appropriate species. Full MS^1^ scans were acquired at 240 000 resolution. MS^2^ spectra were acquired at 15 000 resolution (for both DDA and PRM) with a 1.4 *m*/*z* isolation window. Higher-energy Collision Dissociation (HCD) was used for fragmentation using a fixed normalised collision energy (NCE) of 24 in case of DDA, and a stepped NCE of 22, 26 and 30 in case of PRM. Raw data were manually analysed in XCalibur QualBrowser (Thermo Scientific), with extracted ion chromatograms to the predicted species generated with 10 ppm mass tolerance. MS^2^ spectra corresponding to the predicted mass were manually characterised for ring closures based on predicted neutral loss peaks of non-crosslinked residues.

#### NMR analysis

NMR spectra were recorded at 25 °C. The 1D ^19^F-NMR spectrum was recorded on a Bruker 400 MHz NMR spectrometer, using ^1^H broadband decoupling, a repetition delay of 2.4 s and 20 000 scans. The [^13^C, ^1^H]-HSQC spectra were recorded on a Bruker 800 MHz NMR spectrometer equipped with a TCI cryoprobe, using 12 h for the spectrum of 13/14 and 10 minutes for the spectrum of balhimycin.

#### Disc diffusion bioassays

For antibiotic activity tests of balhimycin variants, concentrated compounds were applied on 6 mm diameter sterile paper discs and dried under the clean bench. *B. subtilis* ATCC 6633 spores and *L. plantarum* WJL were used as test bacteria. *L. plantarum* was grown overnight at 37 °C standing in liquid MRS medium and OD_600_ of 0.5 in 100 μL of 1× PBS was used for plating on MRS agar plates. The plates were incubated overnight at 37 °C and bioactivity was determined by measuring the diameter of the inhibition zones.

#### Susceptibility testing

Minimal inhibitory concentrations of 13/14 and balhimycin against *S. aureus* SG511 and *M. luteus* (Schroeter) Cohn (ATCC 4698) were determined by standard broth microdilution according to the CLSI guidelines^56^ in polypropylene microtiter plates using cation-adjusted MHB (Oxoid). Experiments were performed with three biological replicates.

### 5. Biochemistry

#### Protein expression and purification

PCP-X_tei_ (Tcp12, UniProt protein ID: Q70AZ6), OxyB_bal_ (UniProt protein ID: O87674), OxyA_ris_ (UniProt protein ID: A0A075V230), OxyC_cep_ (UniProt protein ID: O52825 and O52816); PuR (UniProt protein ID: Q6N3B2), PuxB (UniProt protein ID: Q6N2U2), Sfp (R4-4 mutant) and M4 and M5 domains^57^ of Tcp 11 UniProt protein ID: Q70AZ7) were expressed and purified as previously reported.^13,57–60^

#### A-domain characterisation

Activation of phenylglycine substrates 1–3 were tested in triplicate using an established online pyrophosphate^61,62^ and colorimetric assays^63^ together with reported module 5 (A-PCP-C-E) A-domain construct from teicoplanin biosynthesis.^57^

#### Oxy-enzyme activity

Acceptance of peptides 4–8 by the GPA Oxy enzymes were tested using reported conditions.^64,65^ Briefly, the peptidyl-CoAs were loaded onto a PCP-X didomain construct from teicoplanin biosynthesis through the actions of the promiscuous phosphopantetheinyl transferase Sfp (R4-4 mutant).^59^ Peptide loaded PCP-X didomain constructs^13^ were then used as substrates for Oxy enzyme-mediated cyclisation reactions containing either OxyB, OxyA + B or OxyA + B + C. Following incubation, PCP-bound substrates were chemically cleaved as methylamide peptides the addition of methylamine and subsequently isolated using solid phase extraction. The results of Oxy turnover were then characterised by HR-HPLC-MS, with the percentage conversion being determined based on the ratio of cyclised peptide compared to residual starting material. For multi-step Oxy reactions, percentage conversion is calculated from the yield of the previous Oxy step (*i.e.* monocyclic peptide for OxyA and bicyclic peptide for OxyC).^64,65^

### 6. Interaction with the molecular target lipid II

#### Luciferase-based cell wall stress reporter assay

*B. subtilis* luciferase LiaRS reporter assays were performed as previously described.^66^ Briefly, *B. subtilis* W168 sacA::pCHlux101 (P_liaI_-*lux*)^47^ was grown in MHB containing 5 mg mL^−1^ chloramphenicol at 30 °C with agitation to an OD_600_ of 0.5. Cells were added to 96-well white wall chimney plates which contained serially diluted GPAs. Luminescence measurements were performed at 30 °C in a microplate reader Spark 10M (Tecan). At least three independent biological replicate experiments were conducted. Data analysis was performed using Graph Pad Prism 5.01.

#### Synthesis and purification of the peptidoglycan precursor lipid II

Large scale synthesis and purification of lipid II were conducted as previously described.^67^ Briefly, UDP-*N*-acetyl-muramic acid pentapeptide (UDP-MurNAc-pp) which was purified as a crude substrate, 1000 nmol undecaprenyl phosphate (C_55_-P) and 10 μmol UDP-*N*-acetyl-glucosamine (Merck) were incubated with *M. luteus* membranes containing the glycosyltransferases MraY and MurG in 100 mM tris–HCl, 10 mM MgCl_2_, and 0.5% Triton X-100, at pH 7.5 in a total volume of 15 ml at 30 °C for 4 h. Synthesized lipid II was extracted after addition of an equal volume of *n*-butanol/pyridine acetate, pH 4.2 (2 : 1, v/v) and purified *via* HPLC. The concentration of the purified peptidoglycan precursor was quantified on basis of the phosphate content.^68^

#### Complex formation of GPAs with lipid II

Binding of GPAs to lipid II was analysed by incubating 2 nmol of the HPLC-purified cell wall precursor with 2–8 nmol of the respective GPA in 50 mM Tris–HCl, pH 7.5, 10 mM MgCl_2_ in a final volume of 30 μL for 30 min. Free lipid II was extracted from the reaction mixture with an equal volume of *n*-butanol/pyridine acetate, pH 4.2 (2 : 1, v/v), analysed by TLC using chloroform/methanol/water/ammonia (88 : 48 : 10 : 1, v/v/v/v) as the solvent and detected by phosphomolybdic acid staining.^68^

#### Inhibition of lipid II synthesis in vitro

Impact of GPAs on lipid II synthesis was assessed in principle as described above. Briefly, small scale *in vitro* synthesis of lipid II was performed by incubating membrane preparations (200 mg protein) with 5 nmol C_55_-P, 50 nmol UDP-MurNAc-pentapeptide and 50 nmol UDP-*N*-acetylglucosamine in 100 mM tris–HCl, 10 mM MgCl_2_, and 0.5% Triton X-100, at pH 7.5 in a total volume of 50 μl at 30 °C for 2 h. GPAs were added in molar ratios from 1 to 4 with respect to substrate C_55_P. Free lipid-containing peptidoglycan precursors were extracted as described above, analysed by TLC and quantified using Fiji software (National Institutes of Health)^69^. Experiments were performed at least in triplicates.

## Results and discussion

### *A. balhimycina* Δ*hpg* is suitable for mutasynthesis approach

**Fig. 5:**
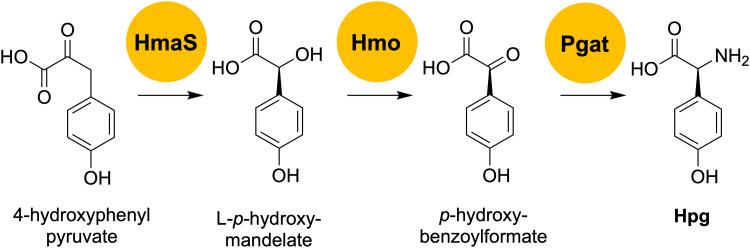
Overview of the final steps of the Hpg biosynthesis pathway. HmaS: 4-hydroxymandelate synthase; Hmo: l-4-hydroxymandelate oxidase; Pgat: amino transferase; Hpg: 4-hydroxyphenylglycine.

To generate an *A. balhimycina* Δ*hpg* mutant suitable for the mutasynthesis of balhimycin, we needed to delete the two key biosynthetic genes *hmaS*, encoding 4-hydroxymandelate synthase, and *hmO*, encoding l-4-hydroxymandelate oxidase (Fig. 5). These deletions prevent the formation of l-*p*-hydroxymandelate, its subsequent oxidation to *p*-hydroxybenzoylformate and thus the transfer of the amino group *via* Pgat to generate Hpg, which is required for balhimycin synthesis.

**Fig. 6:**
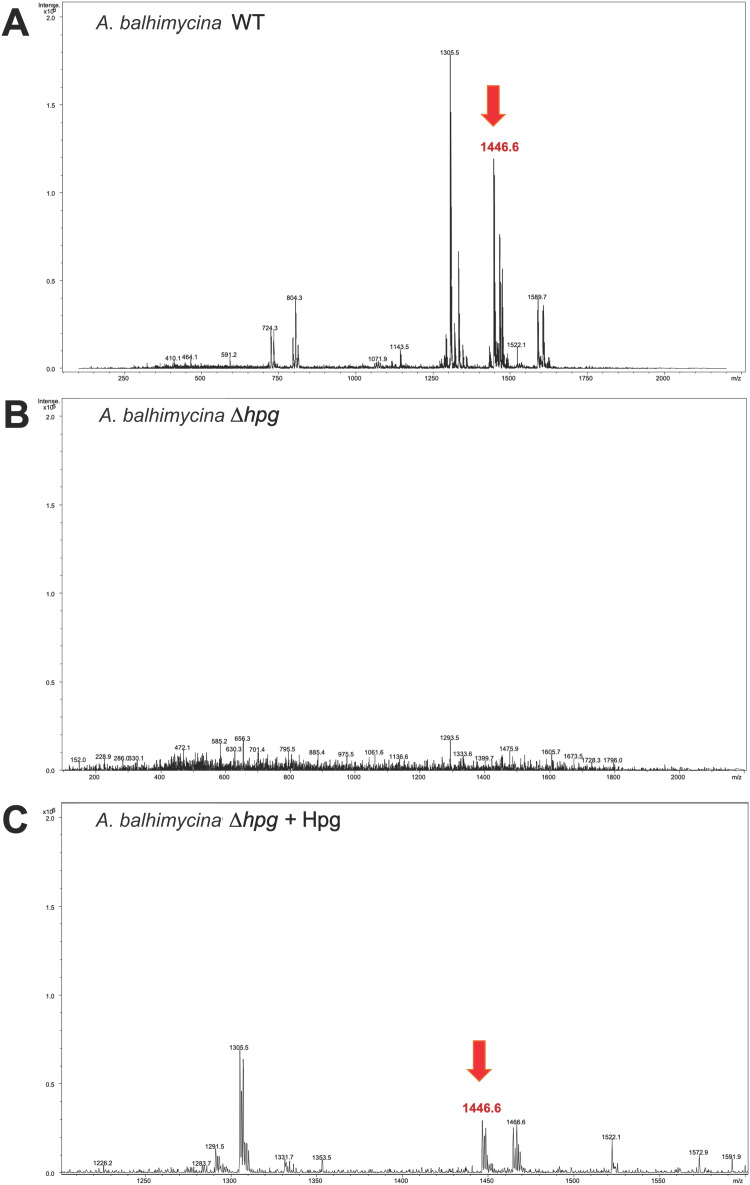
Mass spectra from HPLC-MS analysis of concentrated cultural supernatant from *A. balhimycina* wild type (WT) (A) *A. balhimycina* Δ*hpg* (B) and *A. balhimycina* Δ*hpg* supplemented with 4-hydroxy-l-phenylglycine (Hpg) (C) after cultivation in R5 medium. The detected balhimycin mass (*m*/*z* 1446.6 [M + H]^+^) is indicated by a red arrow.

Deletion of the *hmaS* and *hmo* genes was performed using a non-replicating pSP1 vector carrying an *eryR* cassette for selection. The resulting *A. balhimycina* Δ*hpg* mutant and the *A. balhimycina* wild type (WT) strain (control) were cultivated in R5 medium for 175 h. The concentrated supernatants were analyzed by HPLC-MS, revealing an absence of the balhimycin peak in the chromatogram, thus confirming the inability of *A. balhimycina* Δ*hpg* to produce balhimycin. (Fig. 6(B) and ESI,† Fig. S4). In contrast, the characteristic balhimycin HPLC elution profile pattern and mass (RT 5.3 min with *m*/*z* of 1446.6 [M + H]^+^) were detected in concentrated supernatant samples of *A. balhimycina* WT (Fig. 6(A) and ESI,† Fig. S4). The presence of balhimycin in the extracts was further confirmed by bioactivity assays using *Bacillus subtilis* ATCC 6633 as a test strain. Supplementation of the *A. balhimycina* Δ*hpg* mutant with 4-hydroxy-l-phenylglycine (12 mM) restored balhimycin production (RT 5.3 min with *m*/*z* of 1446.6 [M + H]^+^) (Fig. 6(C) and ESI,† Fig. S4). This demonstrates the successful uptake of external l-Hpg by the mutant strain, followed by its integration into balhimycin by the biosynthetic assembly machinery. Thus, *A. balhimycina* Δ*hpg* proved to be a suitable chassis strain for Phg-based mutasynthesis of balhimycin.

### Supplementation of F-Phg residues demonstrates impaired GPA biosynthesis

**Fig. 7:**
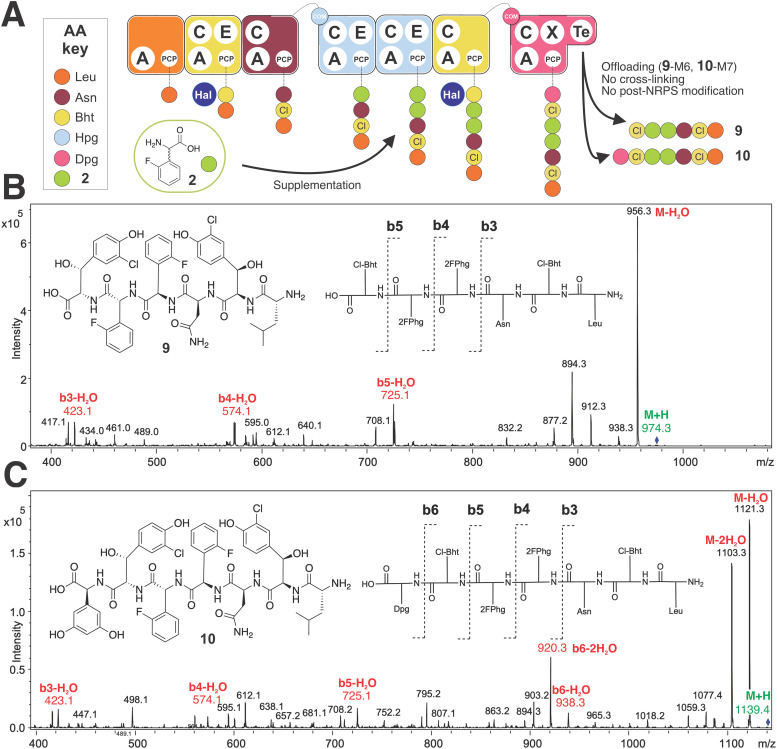
Biosynthetic incorporation of 2-F-Phg 2 into balhimycin biosynthesis *via* mutasynthesis. Schematic of balhimycin biosynthesis showing the incorporation of 2 affording 9 and 10 (A). MS^2^ fragmentation of 9 (B) and 10 (C). Colours are the same as shown in Fig. 1.

To begin our investigations into the mutasynthesis of balhimycin using the *A. balhimycina* Δ*hpg* strain, we explored the supplementation of this strain with F-Phg mutasynthons bearing fluorine residues in either the 2- or 4-position of the aromatic ring 4-fluoro-dl-phenylglycine (4-F-Phg (1)) and 2-fluoro-dl-phenylglycine (2-F-Phg (2)). The mutasynthons were added to the growing culture of *A. balhimycina* Δ*hpg* and concentrated supernatant samples and extracts were analysed using HPLC-MS, HPLC-MS^2^ and HPLC-HRMS methods to investigate the production of possible balhimycin derivatives. Initial analysis of the incorporation of 4-F-Phg ((l) or (d) 1) did not reveal any production of a modified balhimycin peptide or biosynthetic precursors, which was somewhat unexpected given the previous reports of the incorporation of (1) into CDA biosynthesis^30^ (Fig. 2). We next tested the acceptance of 2-F-Phg (2), which resulted in the synthesis of novel fluorinated balhimycin derivatives (Fig. 7(A)). Two compounds (9–10) were detected with an elution time of 8.5 min in both positive ion mode (9, *m*/*z* 974.4[M + H]^+^ and 10, 1139.4 [M + H]^+^) and negative ion mode (9, *m*/*z* 972.3[M − H]^−^ and 10, 1137.4 [M − H]^−^) (ESI,† Fig. S5B and S6A, B). These masses indicated the presence of two new fluorinated balhimycin derivatives, which was confirmed by high-resolution HPLC-MS (ESI,† Fig. S7) and HPLC-MS^2^ analysis (Fig. 7(B) and (C)) (*m*/*z* 974.2713 [M + H]^+^ and 1139.3138 [M + H]^+^). The molecular formula of these compounds were C_44_H_48_Cl_2_F_2_N_7_O_12_ (9) and C_52_H_55_Cl_2_F_2_N_8_O_15_ (10)), which correspond to modified hexapeptide and heptapeptide precursors of balhimycin, respectively (Fig. 7(B) and (C)). The masses of these linear peptides indicated that they were not crosslinked, which was also clearly demonstrated in HPLC-MS^2^ analysis. The characteristic fragmentation pattern observed in these experiments is typical for linear balhimycin hexa- and heptapeptides, albeit here with the presence of two fluorine atoms (Fig. 7(B) and (C)). The lack of subsequent modification of these linear peptides by either glycosyl- or methyltransferases is also in agreement with the specificity of these enzymes for late-stage cyclised GPA intermediates.^70,71^ Additionally, a minor product (11) with a mass of *m*/*z* of 1147.2957 [M + H]^+^ (ESI,† Fig. S5C, S6C and S8) and with the molecular formula C_53_H_51_Cl_2_F_2_N_8_O_15_ was detected in the extracts. This corresponds to a fully tricyclic heptapeptide containing two fluorine atoms as well as an N-terminal methyl group and produced in very small amounts; the absence of the 4-Hpg phenol moiety prevents glycosylation in this case, despite the tricyclic peptide structure. These results indicated that the incorporation of F-Phg residues in GPA biosynthesis appears to be limited in the acceptance of the modified amino acid residues by the respective adenylation domains within module 4 and 5 of the NRPS and/or by the Oxy-mediated peptide cyclisation cascade, which we determined to resolve using biochemical experiments.

### Enzymatic characterisation revealed the acceptance of F-Phg by the GPA biosynthetic machinery

**Fig. 8:**
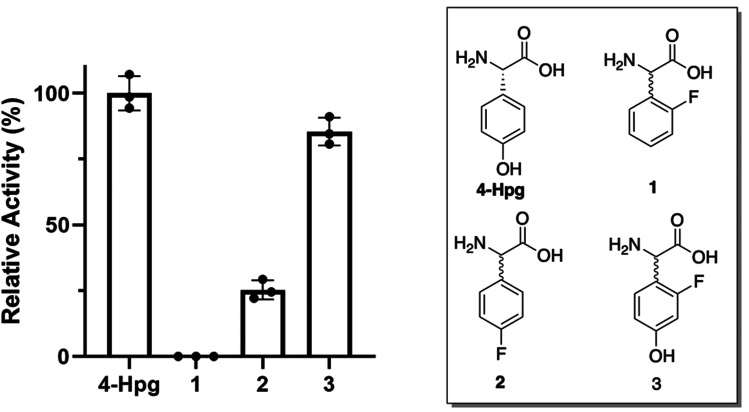
Relative activity of the Hpg-activating A-domain from module 5 of teicoplanin biosynthesis for F-Phg residues 1–2 and F-Hpg 3 compared to the natural substrate 4-Hpg (*n* = 3).

With multiple enzymes that could be contributing to the limited acceptance of modified F-Phg residues 1–2 within GPA biosynthesis, we next explored the acceptance of these modified Phg residues by both the A-domains responsible for their selection and activation and the Oxy-enzymes (Fig. 8). To this end, we first tested the A-domain activation of substrates 1–2 using a module 5 construct from the biosynthetic pathway of the related GPA teicoplanin.^57^ This system was chosen due to challenges associated with the soluble expression of the comparable balhimycin constructs in *E. coli* and the similarity of the Hpg selection pockets found in the A-domains of both the teicoplanin and balhimycin NRPSs,^28^ including the A_1_ domain from teicoplanin biosynthesis that was recently structurally characterised (78.2% ID between A_5_ from teicoplanin and balhimycin, ESI,† Fig. S9 and S10).^19^ The results of these assays showed that the activation of 1 by the NRPS machinery was significantly impaired, with the rate of activation found to be below the detection limit (Fig. 8). The activation of 2 was also reduced compared to Hpg but was still far greater than the activity seen with 1 (∼25% of Hpg activity) (Fig. 8). This showed that the low levels of peptides isolated from the feeding of 2 in mutasynthesis experiments was at least in part due to the reduced acceptance of the Hpg replacement residue by the NRPS machinery, whilst the lack of production seen with the supplementation of 1 was due to the lack of acceptance of this residue.

**Fig. 9:**
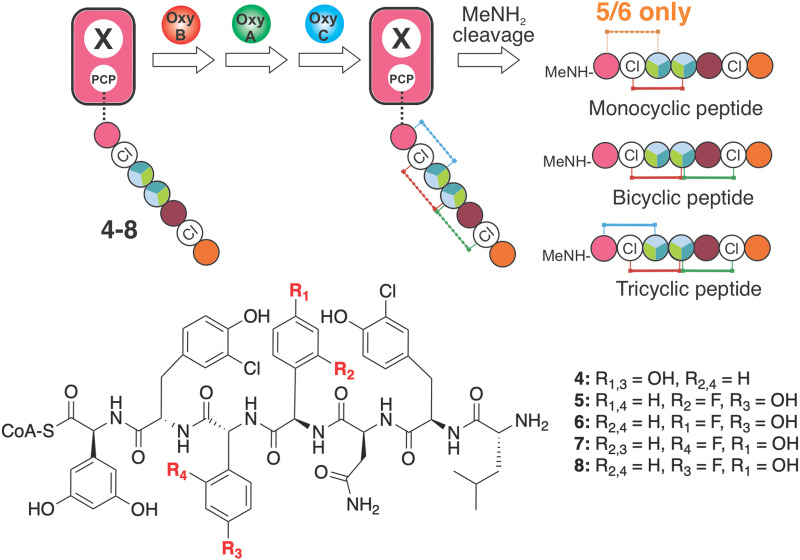
Analysis of the effect of F-Phg residues on Oxy-mediated peptide crosslinking. Schematic representation of the crosslinking assay, in which synthetic heptapeptidyl CoAs 4–8 are first loaded onto a PCP-X didomain from the teicoplanin NRPS through the activities of the transferase Sfp. Subsequent incubation together with Oxy enzymes and redox partners leads to cyclisation of the peptides that are then cleaved *via* the addition of methylamine to generate the peptide methylamides, which are subsequently analysed by HRMS-MS. Amino acids are represented as coloured circles, with the colours are the same as shown in Fig. 1 (plus teal: 4-F-Phg).

Next, we characterised Oxy enzyme activity towards linear heptapeptides bearing a single F-Phg residue (Fig. 9). These peptides (5–8, plus control balhimycin-like peptide 4) were synthesised using solid phase peptide synthesis (SPPS) under modified conditions to overcome the potential racemisation of these residues that is typically seen under standard SPPS conditions (Fig. 9).^51,52^ Each peptide contained either (d)-4-Phg or (d/l)-2-Phg at position 4 or 5 of the peptide and was subsequently converted into the peptidyl-CoA prior to enzymatic loading onto a PCP-X didomain substrate.^13^ Cyclisation assays with these four peptides as well as the balhimycin-peptide control were then undertaken using only the first Oxy enzyme of the cascade (OxyB) (ESI,† Fig. S11, S14, S22, S29 and S36), both the first and second Oxy enzymes (OxyB and OxyA) (ESI,† Fig. S12, S15, S23, S30 and S37) or all three Oxy enzymes OxyA-C (ESI,† Fig. S13, S16, S24, S31 and S38).^64,65^ The results of these turnovers (Table 1) showed that incorporation of either F-Phg residue at position 4 of the peptide structure (5–6) greatly reduced the activity of the first enzyme alone (OxyB, ∼20% of the conversation level seen for 4 (Fig. 9(B) and ESI,† Fig. S14, S22), which would suggest that peptides containing a single crosslink (such as arylomycin)^72^ could be biosynthesised with F-Phg residues within the crosslink, albeit at a reduced level (Table 1). Curiously, the inclusion of either two or three Oxy enzymes then led to greatly reduced conversion by the initial enzyme OxyB for 5–6, which suggests that there could well be inhibition of OxyB by even a low level of bi-/tricyclic peptides present in this assay (for 5 – ESI,† Fig. S14–S21 for 6 – ESI,† Fig. S22–S28).^39^ The level of OxyB conversion alone is also somewhat surprising given the removal of a phenol group from the 4-position in these peptides has been reported to lead to lower cyclisation yields, although mechanistic possibilities exist through which such results can be reconciled.^73^ Perhaps, even more unexpected was the identification of crosslinked peptides containing a crosslink between residues 5 and 7 in the OxyB-mediated turnover reaction of 5 and 6, which to our knowledge are the first examples of OxyB inserting such a ring without prior formation of a C-O-D ring (ESI,† Fig. S18 and S26).^65,73–75^

**Table 1:** Results of the Oxy-mediated turnovers of peptide 4-8 using OxyB alone, OxyB and OxyA or all 3 Oxy enzymes OxyA-C (B, *n* = 3)

Peptides					Oxy enzymes	Conversion		
No.	R_1_	R_2_	R_3_	R_4_		Monocyclic	Bicyclic	Tricyclic
4	OH	H	OH	H	B	78.6 ± 7.5%	<0.5%	—
					BA	71.4 ± 9.7%	10.3 ± 6.5%	—
					BAC	83.3 ± 7.6%	34.6 ± 3.7%	14.2 ± 6.8%

5	H	F	OH	H	B	16.9 ± 10.5%	1.7 ± 2.7%	—
					BA	9.7 ± 1.7%	<0.5%	—
					BAC	18.0 ± 6.7%	<0.5%	ND

6	F	H	OH	H	B	20.7 ± 10.9%	<0.5%	—
					BA	2.9 ± 0.6%	1.0 ± 1.1%	—
					BAC	9.7 ± 2.7%	<0.5%	>0.5%

7	OH	H	H	F	B	34.3 ± 13.5%	6.5 ± 5.5%	—
					BA	28.3 ± 4.3%	7.0 ± 2.3%	—
					BAC	35.8 ± 10.5%	13.8 ± 3.1%	ND

8	OH	H	F	H	B	60.9 ± 27.0%	7.3 ± 11.9%	—
					BA	30.8 ± 2.7%	15.1 ± 3.9%	—
					BAC	31.2 ± 15.0%	15.2 ± 9.5%	<0.5%

Turning to the incorporation of F-Phg residues in position 5 of the peptide (7–8), reasonable OxyB activity was now detected that was also reduced in the presence of additional Oxys (albeit nowhere near as severe). 7–8 also showed reasonable levels of bicyclisation, suggesting that such substrates could be present *in vivo* at least in a bicyclic form (Table 1 and ESI,† Fig. S29–S42). Very limited activity for the final enzyme (OxyC) was detected, which again is in keeping with the importance of maintaining moieties with abstractable protons to enable effective Oxy-mediated peptide crosslinking.^73^ Taken together, the results of these *in vitro* biochemical assays showed that the incorporation of F-Phg residues by the GPA NRPS is poor, and furthermore demonstrated the importance of retaining the OH moiety normally present on Hpg residues to ensure the sequential activity of the Oxy enzymes, which explains the limited formation of 11 in these experiments.

### Mixed feeding mutasynthesis enables the isolation of partially cyclised GPA peptides

**Fig. 10:**
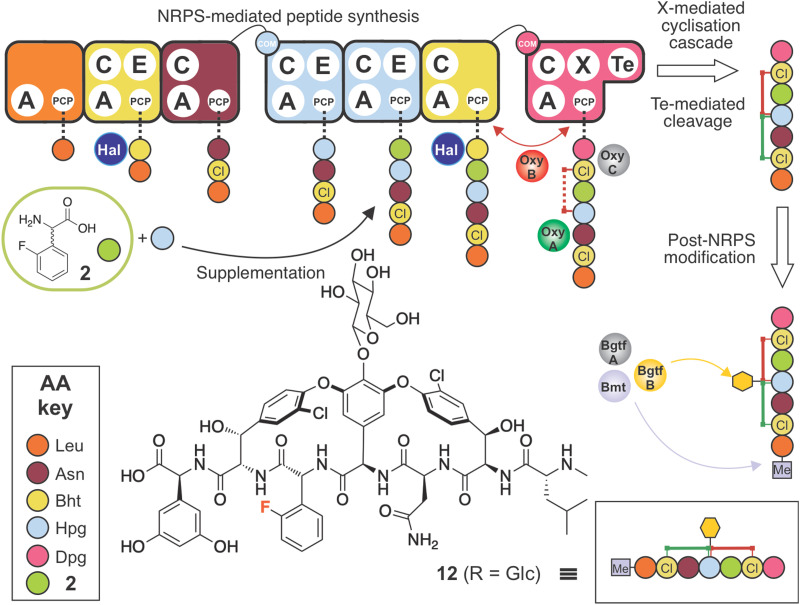
Biosynthetic incorporation of 2-F-Phg 2 into balhimycin biosynthesis *via* mutasynthesis together with supplementation of Hpg. Schematic of balhimycin biosynthesis showing the incorporation of 2 together with Hpg affording 12. Colours are the same as shown in Fig. 1.

Given the results of our *in vitro* assays indicating poor activity of A-domains and Oxy enzymes towards 2-F-Phg 2, we next explored feeding conditions under which we could incorporate both 2 and a Hpg residue during balhimycin biosynthesis in the *A. balhimycina* Δ*hpg* strain (Fig. 10). To this end, we explored a variety of supplementation conditions in which we varied the amount and ratio of both 2-F-Phg and Hpg. The optimised concentration of Hpg (0.02 mg mL^−1^) together with 2-F-Phg (0.6 mg mL^−1^) resulted in detection of a novel balhimycin derivative 12 (*m*/*z* 1309.3 [M + H]^+^) (ESI,† Fig. S5D and S6D), which was further analysed by MS^2^ fragmentation (ESI,† Fig. S43). The data obtained from these experiments supported the structure of 12 comprising a bicyclic heptapeptide core modified with both a glucose moiety (presumably on position 4 of the peptide) and an N-terminal methyl group. Further high resolution HPLC-MS analysis confirmed the mass of this compound as *m*/*z* 1309.3541 [M + H]^+^ (ESI,† Fig. S44) which matches with the anticipated molecular formula (C_59_H_64_Cl_2_FN_8_O_21_) based on the MS^2^ analysis. This result also agrees with the results of our *in vitro* Oxy-cyclisation assays, which showed that effective bicyclisation of linear peptides by the Oxy cascade occurred only with the F-Phg residue in position 5 of the peptide (7–8). The use of mixed feeding experiments to overcome challenges with NRPs encoding two (or more) of the same residues being targeted for mutasynthesis is also likely to be relevant for several other complex NRPs containing Hpg residues (such as feglymycin and ramoplanin).^76,77^

### Synthesis and supplementation of an optimised F-Phg residue enables GPA biosynthesis

**Fig. 11:**
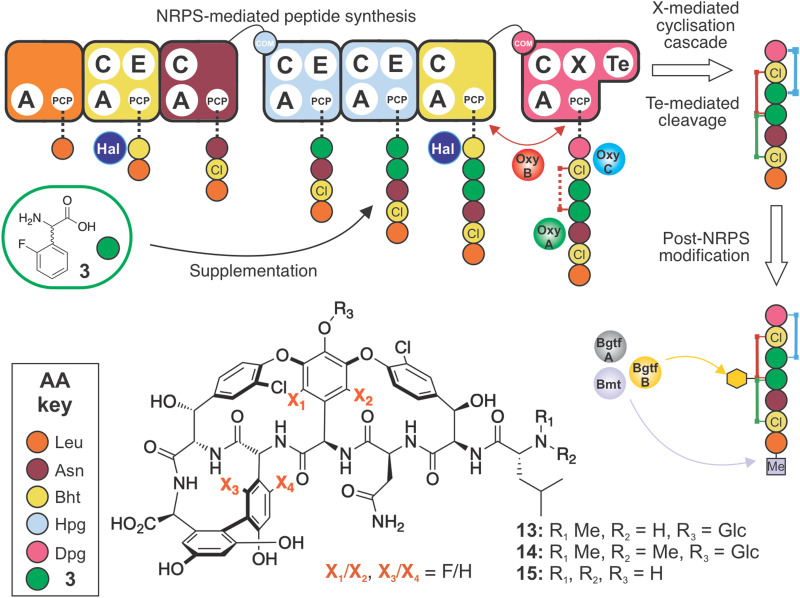
Biosynthetic incorporation of 2-F-Hpg 3 into balhimycin biosynthesis *via* mutasynthesis. Schematic of balhimycin biosynthesis showing the incorporation of 3 affording 13–15. Colours are the same as shown in Fig. 1.

**Fig. 12:**
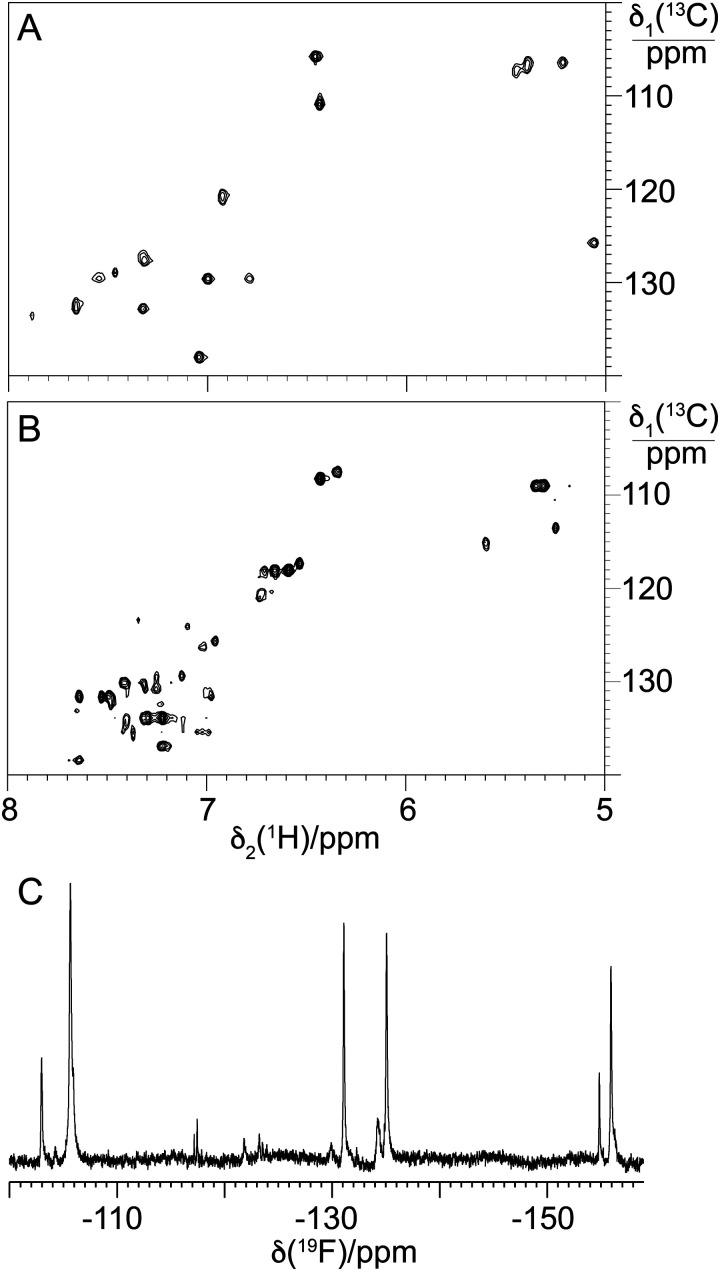
NMR spectra of balhimycin and F-balhimycin. (A) Selected spectral region from the [^13^C,^1^H]-HSQC spectrum of balhimycin, showing the cross-peaks of the aromatic C–H groups. (B) Same as (A) but for F-balhimycin. (C) 1D ^19^F-NMR spectrum of F-balhimycin. The chemical shift axis was calibrated relative to internal TFA at −75.5 ppm.

Having seen the importance of maintaining the 4-OH moiety for successful cyclisation (and as an attachment point for the sugars at residue 4), we next synthesised the compound 2-fluoro-dl-4-hydroxyphenylglycine (2-F-Hpg) (3) in three steps (Fig. 3 and ESI,† Protocol 1) to explore the incorporation of fluorine atoms at both positions 4 and 5 of the balhimycin peptide. After feeding *A. balhimycina* Δ*hpg* with 2-F-Hpg (6 mg in 200 mL culture), the extract was analysed by HPLC-MS. Three new, prominent peaks appeared at RT of 5.8 min, 6.1 min and 7.9 min with masses of *m*/*z* 1341.3 [M + H]^+^ (13), 1355.3 [M + H]^+^ (14) and 1179.3 [M + H]^+^ (15), respectively (Fig. 11 and 12). High resolution MS confirmed the presence of two fluorine atoms in all three of these balhimycin derivatives. The molecular formula for 13 (*m*/*z* 1341.3240 [M + H]^+^) was calculated as C_59_H_61_Cl_2_F_2_N_8_O_22_, for 14 (*m*/*z* 1355.3396 [M + H]^+^) as C_60_H_63_Cl_2_F_2_N_8_O_22_ and for 15 (*m*/*z* 1179.2711 [M + H]^+^) as C_53_H_51_Cl_2_F_2_N_8_O_17_ (ESI,† Fig. S45–S47). Based on this, the structure of 13 corresponds to a tricyclic balhimycin-type heptapeptide containing two fluorine atoms, a d-glucose moiety on Hpg-4, and an N-terminal methyl group (Fig. 11). Compounds 14 and 15 maintain the same tricyclic peptide core as in 13 but with alterations in post-NRPS modification: 14 contains an extra methyl-group at the N-terminus whilst 15 does not contain any modifications to the tricyclic peptide core (Fig. 11). The level of production of these compounds was comparable to that detected for *A. balhimycina* WT (ESI,† Fig. S48), which shows that 4-F-Hpg (3) is a highly effective mutasynthon for the replacement of Hpg within GPA. This is most likely because both phenol moieties are now maintained at residues 4 and 5 of the peptide, which allows the effective cyclisation of the peptide by the Oxy enzyme cascade. The lack of an amino sugar at position 6 of 13 and 14 suggests that there is a difference in the structure of these compounds such that the corresponding glycosyltransferase (BgtfA) is unable to load this sugar, although this was clearly insufficient to prevent the activity of the preceding glycosyl transferase BgtfB^78^ as well as the Oxy enzymes. The low levels of cyclisation observed with mutasynthons 1–2 are reminiscent of the results of *in vivo* deletion experiments that altered the Oxy cascade.^14,43,44^ This once again highlight the importance of an effective cyclisation cascade for high level biosynthesis of GPAs, which in turn stems from the selectivity of the terminal thioesterase domain for highly crosslinked peptides.^79^

### GPA target binding appears influenced by the presence of fluorine atoms

Having seen production of 13–15 by the *A. balhimycina* Δ*hpg* upon feeding with 2-fluoro-dl-4-hydroxyphenylglycine 3, we next scaled the cultures to allow the isolation of larger quantities of these compounds for further characterisation. The removal of 15 from 13/14 was successful, whilst 13/14 did not completely resolve, resulting in the isolation of 4 mg of a mixture of the mono- and dimethylated GPAs (ESI,† Fig. S49). In disc diffusion bioassays for *B. subtilis* (vancomycin-sensitive), the activity of 13/14 was significantly reduced (3.86-fold) compared to balhimycin (ESI,† Fig. S50A and C), while no activity against *L. plantarum* WJL (vancomycin-resistant) was detected (ESI,† Fig. S50D–F). Besides the fluorination, the lack of an amino sugar on residue 6 is a major structural difference between balhimycin and 13/14. Previous analyses have shown that such a modification reduces activity by a factor of 2–5 compared to the doubly glycosylated GPA,^80^ so the reduced activity seen for 13/14 is within this range. Interestingly, it was necessary to dissolve 13/14 in Milli-Q water including 0.002% polysorbate Tween 80 for the activity to occur (ESI,† Fig. S50A and B). This effect was also observed in susceptibility assays for oritavancin using broth microdilution MIC assays.^81^ The antibacterial activity of 13/14 was additionally tested against *S. aureus* SG511,^82^ which is hypersusceptible towards most cell wall biosynthesis inhibitors due to several mutations affecting cell envelope morphology, and *M. luteus* (Schroeter) Cohn (ATCC 4698). Determination of minimal inhibitory concentrations revealed that 13/14 had no activity (MIC >128 μg mL^−1^) against both test strains, while balhimycin showed potent activity against *S. aureus* SG511 (0.25 μg mL^−1^) and *M. luteus* 4968 (0.125 μg mL^−1^).

Given this, and that the activity of BgtfA was inhibited, we next turned to the NMR characterisation of 13/14 to explore the structural effects of the fluorine substituents in these compounds (Fig. 12(C)). Analysis of the aromatic region of the HSQC spectra of 13/14 showed far greater complexity in this region in comparison to balhimycin (Fig. 12(A) and (B)), suggesting that the fluorine atoms within 13/14 were present in different orientations. This was confirmed by the presence of 4 major peaks in the fluorine NMR spectrum (Fig. 12(C)), indicating the presence of mixed regioisomers in 13/14. When combined with the greatly reduced activity of these fluorinated derivatives, the NMR data suggests that the positions of these fluorine atoms are generally deleterious to GPA activity, possibly by occlusion of the d-Ala-d-Ala binding pocket.

**Fig. 13:**
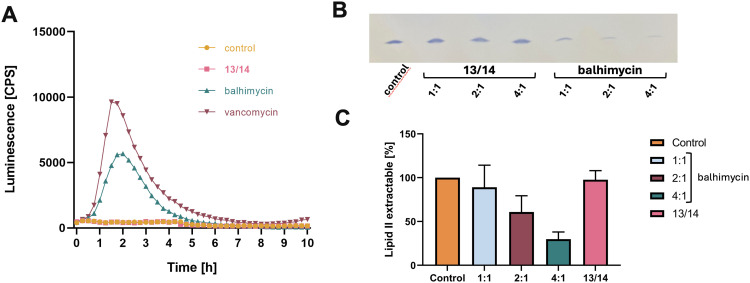
The LiaRS stress response in *Bacillus subtilis* is not induced by 13/14 (A). P_liaI_-*lux* induction in *B. subtilis.* W168 *sacA*::pCHlux101 was monitored over time after addition of 13/14 (0.5 μg mL^−1^, pink line). Balhimycin (0.5 μg mL^−1^, cyan line) and vancomycin (0.25 μg mL^−1^, purple line) were used as control antibiotics. Substitution of balhimycin with fluorine atoms affects the d-Ala-d-Ala binding pocket preventing complexation of lipid II (B). Balhimycin, but not 13/14, forms an extraction stable complex with the ultimate peptidoglycan precursor lipid II indicated by reduction of free lipid II detectable after addition of increasing amounts of GPA. In contrast to 13/14, balhimycin inhibits the synthesis of lipid II catalysed by membranes containing the enzymes MraY and MurG *in vitro* in a dose-dependent manner (C). Antibiotics were added in molar ratios from 1 to 4 with respect to the amount of the substrate C_55_-P. The amount of reaction product synthesized in the absence of antibiotic was set as 100%. Mean values from at least three independent experiments are shown. Error bars represent standard deviation.

GPA binding to lipid II accessible on the bacterial cell surface results in inhibition of transglycosylation and transpeptidation reactions, which elicits cell wall stress that can be monitored using specific bioreporter strains. The LiaRS two-component system (TCS) is known to respond to **l**ipid II biosynthesis cycle **i**nterfering **a**ntibiotics and induction of P_liaI_-*lux* monitored over time was used to determine the cell wall inhibitory effect of the fluorinated GPA derivatives 13/14.^47,83^ Evaluating expressed bioluminescence revealed a strong, concentration-dependent induction of the LiaRS-mediated cell wall stress response by balhimycin and vancomycin (Fig. 13(A) and ESI,† Fig. S51), while the addition of 13/14 had no effect.

To study, whether 13/14 is still able to interact with the purified molecular target lipid II *in vitro*, we tested for complex formation and the impact of balhimycin and 13/14 on the synthesis of lipid II. Whereas balhimycin forms an extraction-stable complex with lipid II and inhibits the formation of lipid II in a dose-dependent manner (Fig. 13(B) and (C)), the interaction of 13/14 with lipid II is significantly reduced confirming the hypothesis that the addition of fluorine atoms dramatically affects the binding of the d-Ala-d-Ala terminus of lipid II.

## Conclusion

The growing threat of GPA-resistant bacteria highlights the need for inventive approaches to alter GPAs to combat the escalation of antibiotic resistance. Although chemical modifications have shown improvements in GPA properties, an ability to manipulate the backbone using the biosynthetic pathway is essential for their commercial production. Mutasynthesis, which involves hijacking the biosynthetic machinery, is a promising solution for achieving this objective. Incorporating unnatural amino acids at consecutive positions in NRPs, such as Hpg residues in GPAs, is a particularly challenging task as it necessitates a high degree of flexibility in the NRPS assembly line. The GPA modification process following peptide assembly is further complicated due to the positioning of Hpg residues in the backbone and the ordered cyclization cascade catalysed by the Oxy enzymes. This means that for GPAs modifications that impact the peptide backbone affect both the NRPS and the oxygenases, making the task of finding effective substitutions more difficult. To understand the intricate interactions involved, a multifaceted approach is necessary. Our research utilises biochemical experiments and molecular biology methods to provide insights into the specificity of NRPS and oxygenases, which are essential for the long-term goal of generating novel GPAs. Our results show that substituted Hpg residues can be incorporated successfully to produce modified GPAs at scale, and further that recent insights into the selectivity of Hpg A-domains offers possible routes to expand mutasynthesis approaches for the generation of new GPAs. The limited activity seen by the fluorinated GPA analogues produced here also demonstrates how well tuned the GPA scaffold is for interactions with lipid II and highlights the necessity for future studies to incorporate computational analysis of product complexes to triage for such modifications prior to undertaking mutasynthesis experiments.

## Conflicts of interest

There are no conflicts to declare.

## Supplementary Material

CB-OLF-D4CB00140K-s001
