# Supplementary material for: Altering glycopeptide antibiotic biosynthesis through mutasynthesis allows incorporation of fluorinated phenylglycine residues
Source: RSC Chem Biol. 2024 Aug 12;5(10):1017–34. doi: 10.1039/d4cb00140k (PMC11376024; doi:10.1039/d4cb00140k)
Supplement: CB-005-D4CB00140K-s001 [file CB-005-D4CB00140K-s001.pdf]

Supplementary Information (SI) for RSC Chemical Biology.
This journal is © The Royal Society of Chemistry 2024

Altering glycopeptide antibiotic biosynthesis through mutasynthesis allows incorporation of fluorinated phenylglycine residues

Voitsekhovskaia and Ho et al.

Supplementary Information

Supplementary Tables .... **Page 2**

Supplementary Figures .... **Page 3**

Supplementary Protocols .... **Page 46**

Supplementary References .... **Page 48**

Supplementary Tables

Table S1. Bacterial strains, plasmids and primers used in this work.

Strain/plasmid/primers	Features	Source or reference
A. balhimycina	Balhimycin producing strain/wild-type, natural isolate of *A. balhimycina* DSM5908	Nadkarni *et al*., (1994)[1]
Δ*hpg*	*hmaS*, *hmO* genes disruption in the balhimycin biosynthetic gene cluster	This study
E. coli		
XL-1 blue	Host strain for routine cloning procedure; genotype: recA1 endA1 gyrA96 thi-1 hsdR17 supE44 relA1 lac [F´ proAB lacI^q ZΔM15 Tn10 (*Tet^R*)].	Agilent Technologies
ET12567	pUZ8002, methylation-defective strain (*dam-13*::Tn*9* *dcm-6 hsdM Cm^R*)	Bierman *et al*., 1992[2]
**Bioassay test strains**		
Bacillus subtilis ATCC 6633	Bioassay test strain	Pelzer *et al*., 1999[3]
Lactobacillus plantarum WJL	Bioassay test strain	Siezen *et al*. 2010[4]
Staphylococcus aureus SG511	Bioassay test strain	Dietrich *et al.*, 2021[5]
Micrococcus luteus (Schroeter) Cohn (ATCC 4698)	Bioassay test strain	American Type Culture Collection
Bacillus subtilis W168 sacA::pCHlux101 (P_{liaI}-*lux*)	Luciferase bioreporter strain	Radeck *et al.*, 2016[6]
**Plasmids**		
pJET1.2/blunt	cloning vector, *lacZ'*-complementation system, *Amp^R*	Thermo Fischer Scientific
pSP1	gene disruption vector, *Ery^R*	Pelzer *et al*., 1997[7]
pSP1hpg-inactFr1+Fr2	contains a 3.095 bp fragment for *hmaS, hmO* gene deletion	This study
**Primers**	**5'→3'**	
hpg-inactFr1fw	TTACTGCAGCCGAGATCCTCG	This study
hpg-inactFr1rev	TTATCTAGAGTCGTCGGCGTTGAAG	This study
hpg-inactFr2fw	TTATCTAGAGAAGTGCTGCTGGACG	This study
hpg-inactFr2rev	TTAGAATTCGTAGACCTCCACGCTG	This study
Ery-fw	CGGCCCGATCTGCCG	This study
Ery-rev	CTACCGCTGCCCGGG	This study
hpg-inact-hpg-fw-check	CGGCCCCCGCATCCC	This study
hpg-inact-hpg-rev-check	GGCCTGCGCCTAGAGACT	This study

*Tet^R - Tetracycline repressor protein; Cm^R – chloramphenicol resistance cassette; Amp^R – ampicillin resistance cassette; Ery^R – erythromycin resistance cassette.

Supplementary Figures

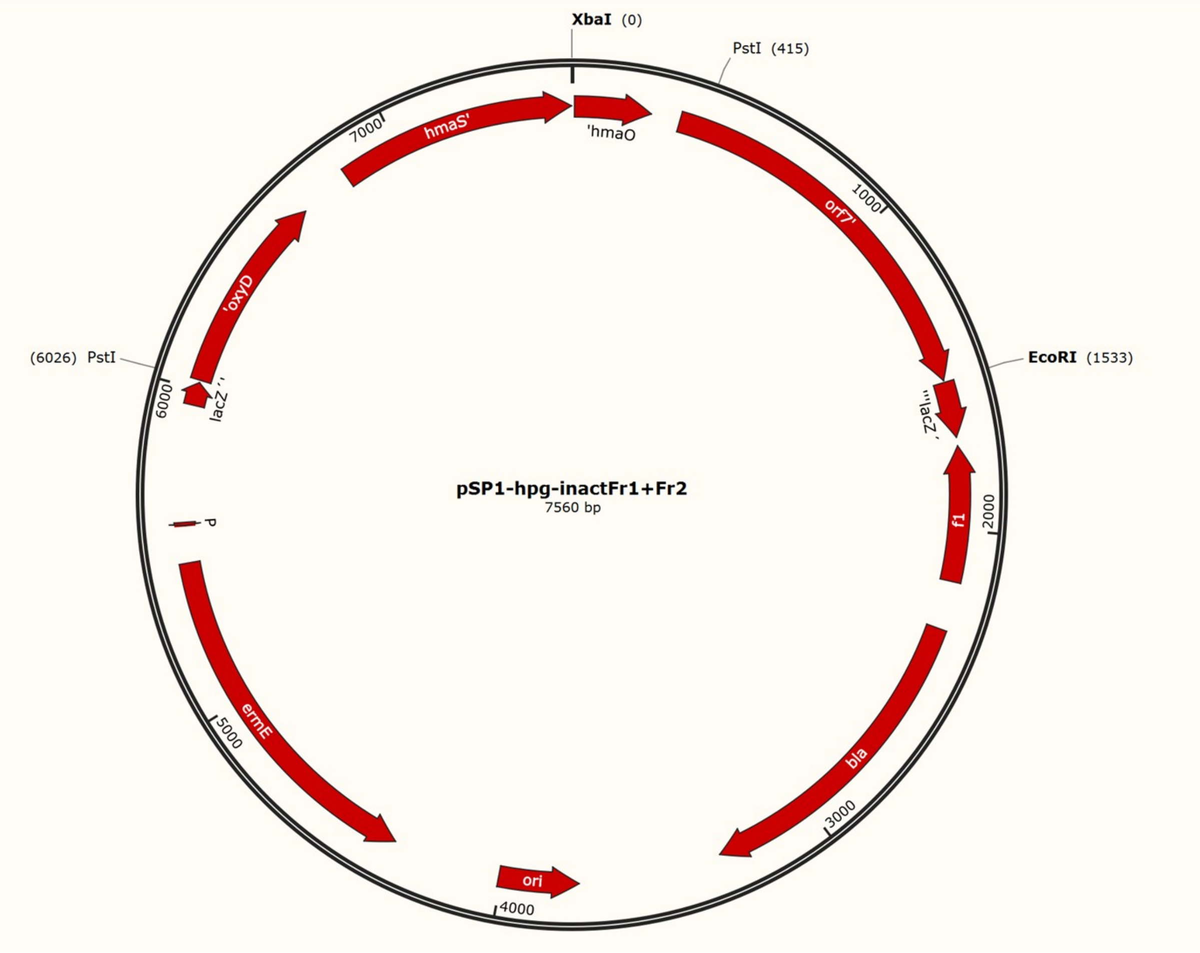

Figure S1. Map of the cloned construct pSP1-hpg-inactFr1+Fr2 for *hmaS*, *hmO* gene deletion.

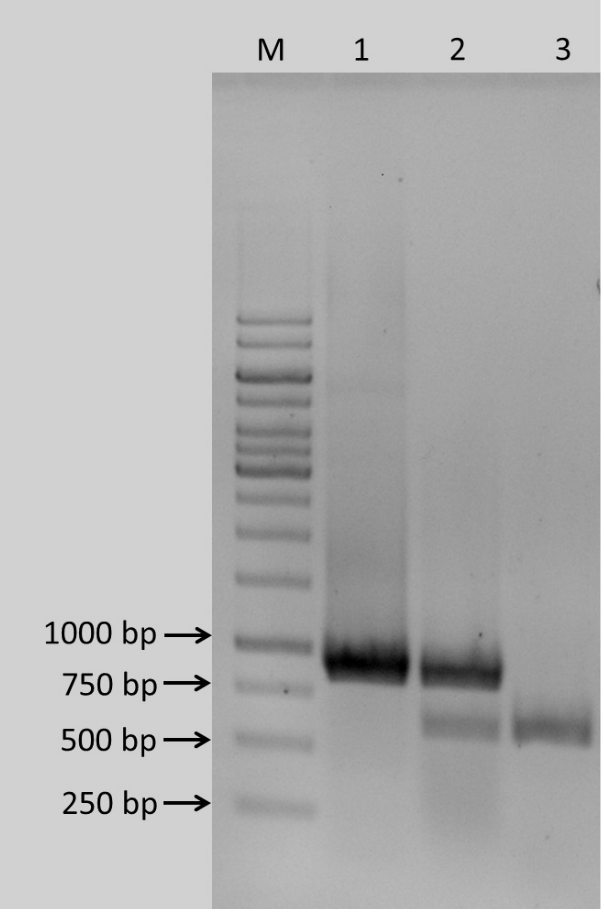

Figure S2. 1% Agarose gel image for verification of the integration of pSP1-hpg-inactFr1+Fr2 in *A. balhimycina* wild type by PCR analysis. Amplicons are shown with primer pairs Ery-fw/rev. Column legend: lane 1: ≈0.9 kb – control plasmid, lane 2 ≈0.9 kb – the *A. balhimycina* containing integrated pSP1-hpg-inactFr1+Fr2, lane 3 - *A. balhimycina* wild type as the negative control, marker (M): 1 kb length marker, Thermo Fisher Scientific.

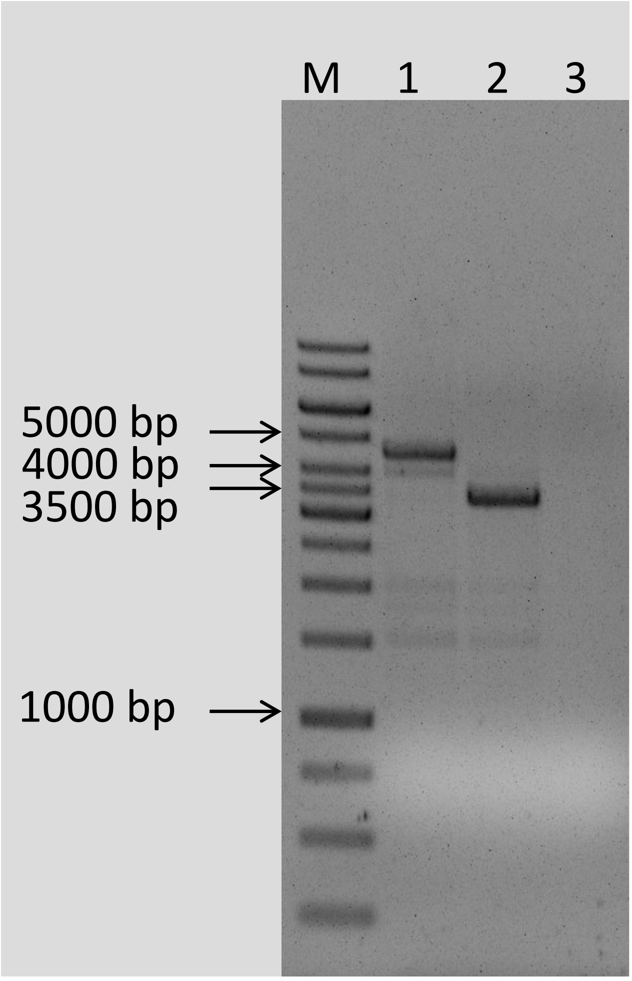

Figure S3. 1% Agarose gel image for verification of the *hmaS, hmO* deletion in *A. balhimycina* by PCR analysis. Amplicons are shown for PCR with primer hpg-inact-hpg-fw-check/rev-check. Column legend: lane 1: ≈ 4.5 kb - *A. balhimycina* wild type as negative control, lane 2: ≈ 3.7 kb - *A. balhimycina* Δ*hpg*, lane 3: milliQ water control, marker (M): 1 kb length marker, Thermo Fisher Scientific.

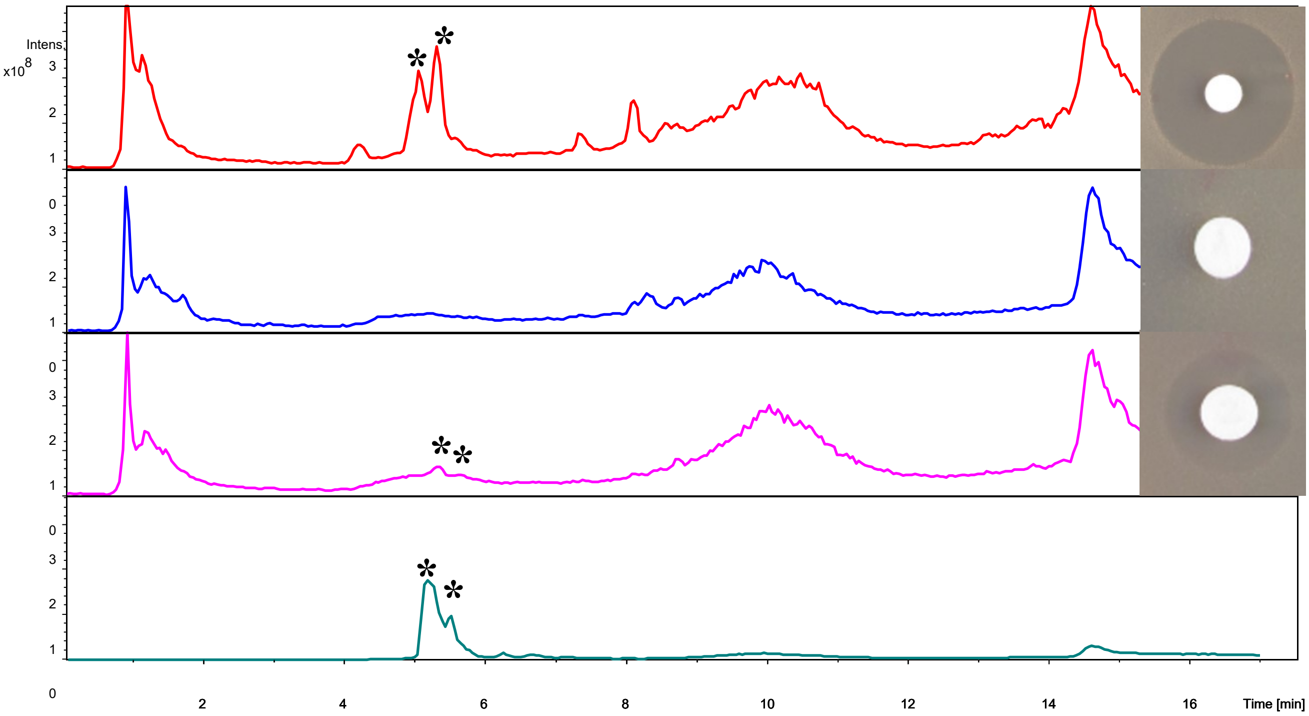

Figure S4. **HPLC-MS chromatograms (TIC, positive ion mode) of concentrated culture filtrate after cultivation of *A. balhimycina* strains in R5 medium.** Red line: *A. balhimycina* wild type, blue line: *A. balhimycina* Δ*hpg*, purple line: *A. balhimycina* Δ*hpg* supplemented with 4-hydroxy-L-phenylglycine, green line: balhimycin standard. The asterisk (*) indicates the peaks corresponding to the balhimycin molecule with *m/z* 1446.6 $[M+H]^+$, detected in both *A. balhimycina* wild type (red line) and *A. balhimycina* Δ*hpg* supplemented with 4-hydroxy-L-phenylglycine (purple line), as well as the balhimycin standard (green line). The bioassays of the concentrated culture filtrates against *B. subtilis* ATCC6633 are presented on the right side.

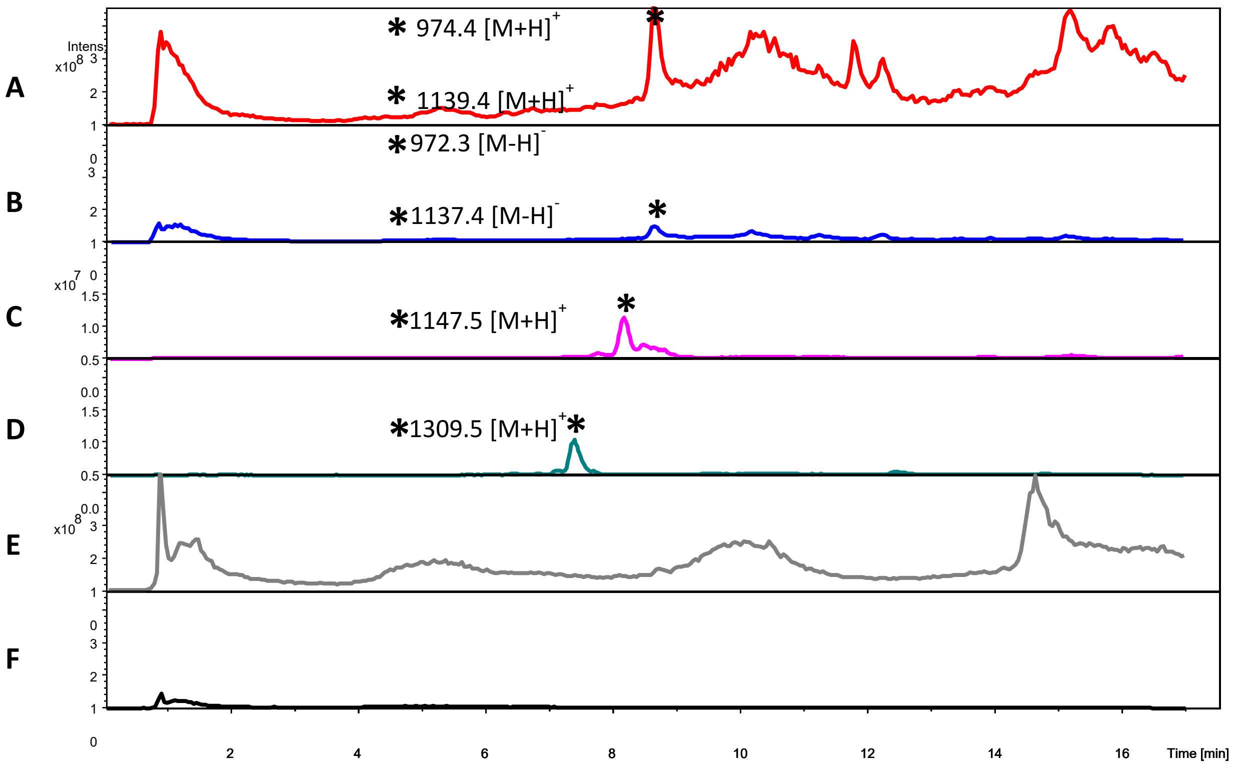

Figure S5. HPLC chromatograms of concentrated culture filtrate after cultivation of *A. balhimycina* *Δhpg* supplemented with 2-fluoro-DL-phenylglycine (A-C) and both 2-fluoro-DL-phenylglycine and 4-hydroxy-L-phenylglycine (D). (A-B). Red line: TIC in positive and blue line: negative ion mode; two mixed and modified hexapeptide and heptapeptide precursors of balhimycin were detected, containing fluorinated substitutions and were non-crosslinked. Peaks indicated with an asterisk (*) correspond to balhimycin derivatives with masses *m/z* of 974.4 $[M+H]^+$ and 1139.4 $[M+H]^+$ in positive ion mode and *m/z* of 972.3 $[M-H]^-$ and 1137.4 $[M-H]^-$ in negative ion mode; (C). Purple line: EIC in positive ion mode; a fully tricyclic heptapeptide containing two fluorine atoms and an N-terminal methyl group was detected, indicated with an asterisk (*) for *m/z* of 1147.5 $[M+H]^+$; (D). Green line: EIC in positive ion mode of cultural filtrate of *A. balhimycina* Δ*hpg* supplemented with both 2-fluoro-DL-phenylglycine and 4-hydroxy-L-phenylglycine; a novel balhimycin derivative with a bicyclic heptapeptide core modified with both a glucose moiety and an N-terminal methyl group was identified, indicated with an asterisk (*) sign for *m/z* of 1309.5 $[M+H]^+$. (E). TIC in positive ion mode, negative control *A. balhimycina Δhpg*; (F). TIC in negative ion mode, negative control *A. balhimycina Δhpg*.

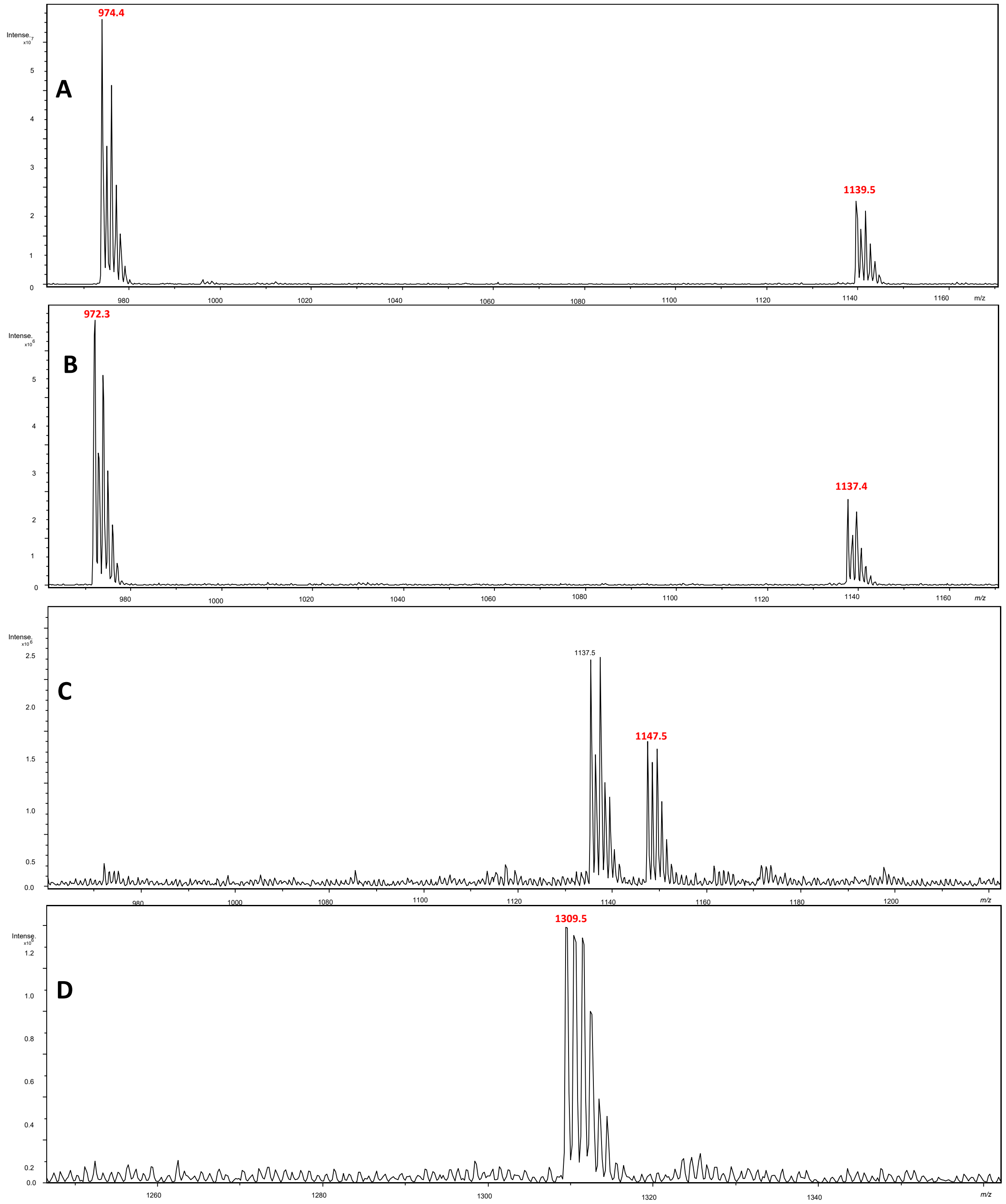

Figure S6. The mass spectra from LC-MS analysis of concentrated culture filtrate of *A. balhimycina* Δ*hpg* supplemented with 2-fluoro-DL-phenylglycine (A-C). (A-B): The novel detected masses with *m/z* of 974.4 [M+H]$^+$ and 1139.4 [M+H]$^+$ in positive ion mode and *m/z* of 972.3 [M-H]$^-$ and 1137.4 [M-H]$^-$ in negative ion mode correspond to modified hexapeptide and heptapeptide precursors of balhimycin containing fluorinated substitutions and which are non-crosslinked; (C). EIC with *m/z* of 1147.2933 [M+H]$^+$ in positive ion mode corresponds to a fully tricyclic heptapeptide containing two fluorine atoms and an N-terminal methyl group; (D). EIC in positive ion mode of culture filtrate of *A. balhimycina* Δ*hpg* supplemented with both 2-fluoro-DL-phenylglycine and 4-hydroxy-L-phenylglycine, where the *m/z* of 1309.5 [M+H]$^+$ was detected and characterized as a bicyclic heptapeptide core modified with both a glucose moiety and an N-terminal methyl group. All masses are identified in positive ion mode are marked with red numbers.

Mass Spectrum SmartFormula Report

Analysis Info

Analysis Name	E:\Iriana\Mirobiol_Irina_IV-Fluorobalh_GE6_01_46221.d	Acquisition Date	12/17/2020 8:57:57 PM
Method	automsms_50-1800_pos_spez.fraglist_xx_0.3.m	Operator	BDAL@DE
Sample Name	Mirobiol_Irina_IV-Fluorobalh	Instrument	maXis 288882.21253
Comment			

Acquisition Parameter

Source Type	ESI	Ion Polarity	Positive	Set Nebulizer	2.0 Bar
Focus	Not active	Set Capillary	4500 V	Set Dry Heater	200 °C
Scan Begin	50 m/z	Set End Plate Offset	-500 V	Set Dry Gas	8.0 l/min
Scan End	1800 m/z	Set Charging Voltage	0 V	Set Divert Valve	Waste
		Set Corona	0 nA	Set APCI Heater	0 °C

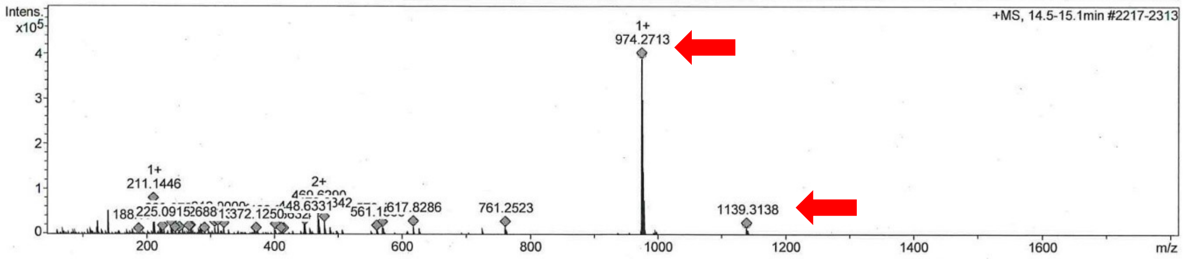

Meas. m/z	#	Ion Formula	m/z	err [ppm]	Mean err [ppm]	rdb	N-Rule	e⁻ Conf	mSigma	Std I	Std Mean m/z	Std I VarNorm	Std m/z Diff	Std Comb Dev
974.271308	1	C42H36Cl2F2N21O2	974.270048	-1.3	-1.8	34.0	ok	even	10.1	6.7	n.a.	n.a.	n.a.	n.a.
→	2	C44H48Cl2F2N7O12	974.270058	-1.3	-1.2	23.0	ok	even	13.0	10.9	n.a.	n.a.	n.a.	n.a.
	3	C45H44Cl2F2N11O8	974.271396	0.1	0.1	28.0	ok	even	15.7	11.1	n.a.	n.a.	n.a.	n.a.
	4	C48H52Cl2F2NO14	974.272744	1.5	1.9	22.0	ok	even	21.7	16.0	n.a.	n.a.	n.a.	n.a.
	5	C46H40Cl2F2N15O4	974.272733	1.5	1.3	33.0	ok	even	23.5	16.2	n.a.	n.a.	n.a.	n.a.
	6	C61H44Cl2F2N3O3	974.272231	0.9	1.6	40.0	ok	even	90.3	58.4	n.a.	n.a.	n.a.	n.a.
1139.313819	1	C50H43Cl2F2N22O5	1139.312641	-1.0	-1.2	39.0	ok	even	34.2	21.6	n.a.	n.a.	n.a.	n.a.
→	2	C52H55Cl2F2N8O15	1139.312651	-1.0	-0.7	28.0	ok	even	35.2	24.5	n.a.	n.a.	n.a.	n.a.
	3	C53H51Cl2F2N12O11	1139.313989	0.1	0.4	33.0	ok	even	40.2	25.7	n.a.	n.a.	n.a.	n.a.
	4	C56H59Cl2F2N2O17	1139.315337	1.3	1.9	27.0	ok	even	46.4	29.9	n.a.	n.a.	n.a.	n.a.
	5	C54H47Cl2F2N16O7	1139.315326	1.3	1.4	38.0	ok	even	47.4	28.5	n.a.	n.a.	n.a.	n.a.

Mirobiol_Irina_IV-Fluorobalh_GE6_01_46221.d
Bruker Compass DataAnalysis 4.4 printed: 2/5/2021 2:27:44 PM by: MS Page 1 of 2

Figure S7. The mass spectrum of high-resolution HPLC-MS analysis of concentrated culture filtrate from *A. balhimycina Δhpg* supplemented with 2-fluoro-DL-phenylglycine. The mass indicated with the red arrow for *m/z* 974.2713 $[M+H]^+$ corresponds to a fluorinated hexapeptide with molecular formula $C_{44}H_{48}Cl_2F_2N_7O_{12}$; the mass indicated with the red arrow for *m/z* 1139.3138 $[M+H]^+$ corresponds to a fluorinated heptapeptide with molecular formula $C_{52}H_{55}Cl_2F_2N_8O_{15}$.

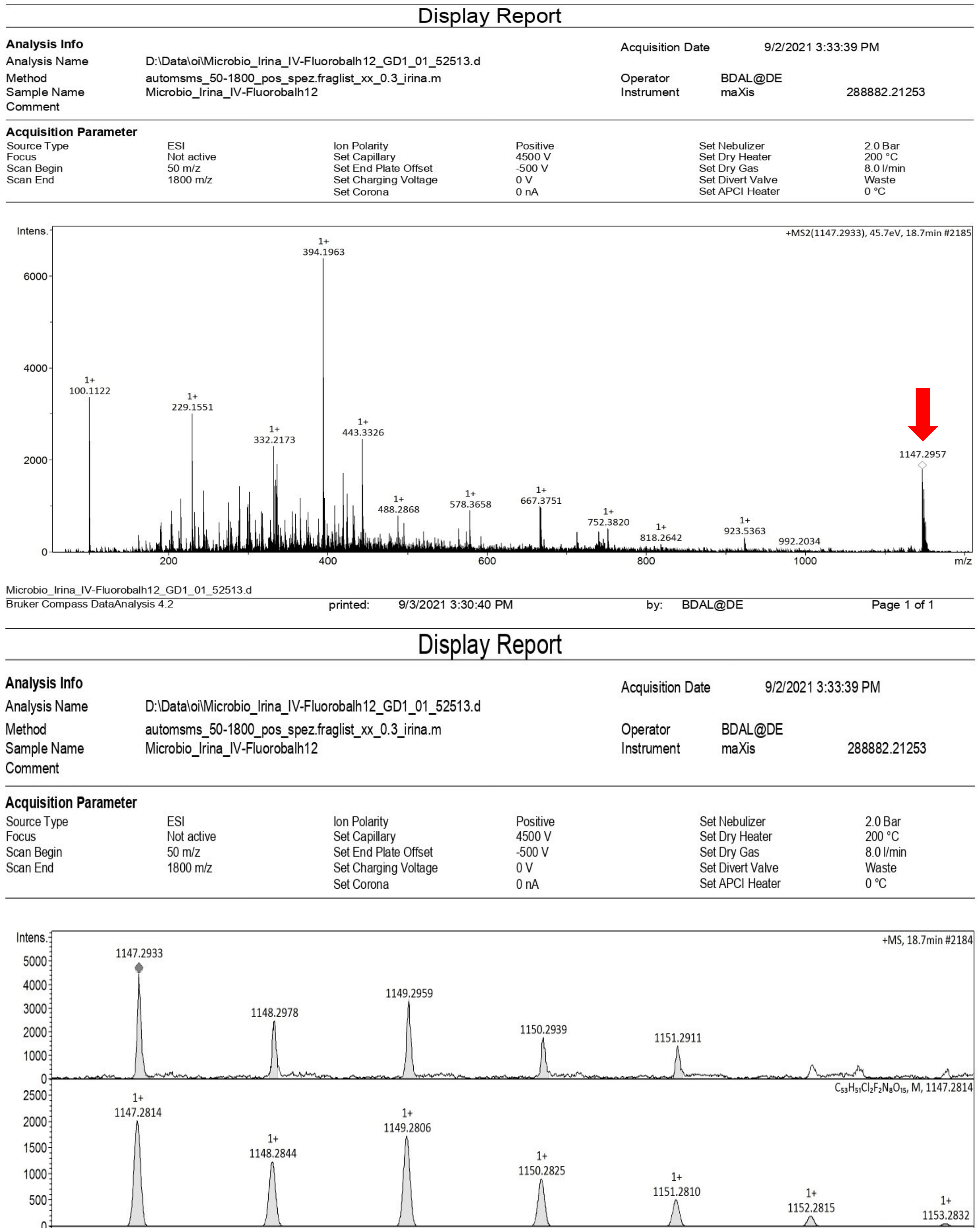

Figure S8. The mass spectrum from high-resolution LC-MS analysis of concentrated cultural liquid from *A. balhimycina Δhpg* supplemented with 2-fluoro-DL-phenylglycine. The mass indicated with the red arrow for *m/z* 1147.2957 $[M+H]^+$ corresponds to fully tricyclic heptapeptide containing two fluorine atoms as well as an N-terminal methyl group with molecular formula $C_{53}H_{51}Cl_2F_2N_8O_{15}$.

```
BpsB_A5         LFRRQAGASPDAVAVVAGERTLSYADLDRESDRLAGHLAGIGVGRGDRVGVVMTRGADLF   60
Tcp11_A5        GFRRRARSSPDAVAVSGGGRTLSYAALDRESDRLAGHLAGRGVRRGDRVGVVMERGTDLF   59
                 ***:* :******* .* ****** ************** ** ********* **:***

BpsB_A5         VALLGVWKAGAAQVPVNVDYPAERIERMLADVGASVAVCVEATRKAVPDGVEPVVVDLPV   120
Tcp11_A5        VALLAIGKAGAAYVPVNLDYPRDRIERMLTDAGVSVAVCVQATSGAVPDGLAPVVMDSPA   119
                ****.: ***** ****:*** :******:*.*.******:**  *****: ***:* *.

BpsB_A5         IGGVRPEAPPVTVGAHDVAYVMYTSGSTGVPKAVAVPHGSVAALASDPGWSQGPGDCVLL   180
Tcp11_A5        IAAAPSEAPPITVGAHDLAYVMYTSGSTGVPKGVAVPHGSVAALAGDPGWSVGPGDGVLM   179
                *...  ****:******:**************.************.***** **** **:

BpsB_A5         HASHAFDASLVEIWVPLVSGARVLVAEPGTVDAERLREAVSRGVTTVHLTAGAFRAVAEE   240
Tcp11_A5        HAPHAFDASLLEIWVPLLSGAHVVVADPGAVDAQRLREAIDRGVTTVHLTAGSFRVLAEE   239
                ** *******:******:***:*:**:**:***:*****:.***********:**.:***

BpsB_A5         SPDSFIGLREILTGGDAVPLASVVRMRQACPDVRVRQLYGPTEITLCATWLVLEPGAATG   300
Tcp11_A5        SPDAFRGLREVLTGGDAVPLASVVRLRETCPEIRVRHLYGPTETTLCATWHLIEPGVATG   299
                ***:* ****:**************:*::**::***:****** ****** ::***.***

BpsB_A5         DVLPIGRPLAGRQAYVLDAFLQPVAPNVTGELYLAGAGLAHGYLGNTAATSERFVANPFS   360
Tcp11_A5        DTLPIGRPLAGRRAYVLDAFLQPVAPNVTGELYLAGAGLARGYLGAAAATAERFVADPFA   359
                *.**********:***************************:**** :***:*****:**:

BpsB_A5         GGGRMYRTGDLARWTDQGELVFAGRADSQVKIRGYRVEPGEVEVALTEVPHVAQAVVVAR   420
Tcp11_A5        AGERMYRTGDLARWTEQGELLFAGRADAQVKIRGYRVEPAEIEAALTAIPEVAQAVVVAR   419
                .* ************:****:******:***********.*:*.*** :*.*********

BpsB_A5         EGQPGEKRLIAYVTAEAGSALESAAVRAHLATRLPEFMVPSVVVVLESFPLTLNGKIDRA   480
Tcp11_A5        EDGPGEKRLIAYVTAAGQPGPDPAAVREHLGERLPEFMVPAAVVVLDGFPLTINGKIDRA   479
                *. ************ .  . : **** **. ********:.****:.****:*******

BpsB_A5         AL 482
Tcp11_A5        AL 482
                **
```

Figure S9. Comparison of A-domain sequences for module 5 A-domains from balhimycin (BpsB) and teicoplanin (Tcp11) biosynthesis. The sequence identify of these domains is 78.2%. A-domain specificity code residues are highlighted in green, with additional Glu residue important for Hpg-activating domains highlighted in cyan.

Protein	A-domain selectivity code[8]											Description
	1	2	3	*	4	5	6	7	8	9	10	
A_5 Teicoplanin	D	A	L	E	H	L	G	L	L	C	K	Hpg pocket module 5
A_5 Balhimycin	D	A	V	E	H	L	G	L	L	C	K	
A_1 Teicoplanin	D	A	F	E	H	L	G	L	L	C	K	Hpg pocket module 1 (structurally characterised)[9]

* Residue outside of canonical 10 AA code important for controlling His-4 rotamer

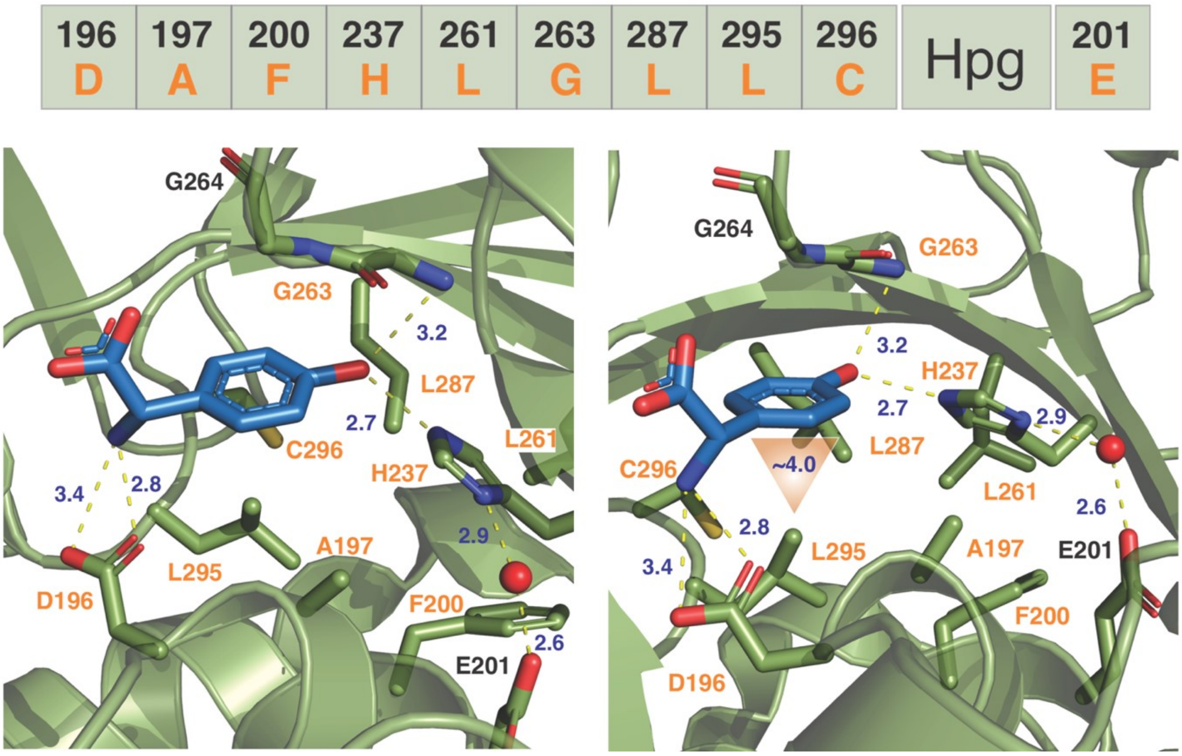

Figure S10. Analysis of A-domain specificity codes. Comparison of the A-domain selectivity codes for the module 5 A-domains from balhimycin (BpsB) and teicoplanin (Tcp11) biosynthesis with that of the structurally characterised module 1 A-domain from teicoplanin (Tcp9)[9] (Upper). Structure of A_1 domain from teicoplanin biosynthesis (lower). The only difference in the code between these Hpg-activating A-domains is found at position 3, which is a hydrophobic residue that faces away from the substrate.[9]

MS Analysis of *in vitro* Turnovers (SI Figure S11 - S42)

1 Turnover of PCP-X_{tei} control 4

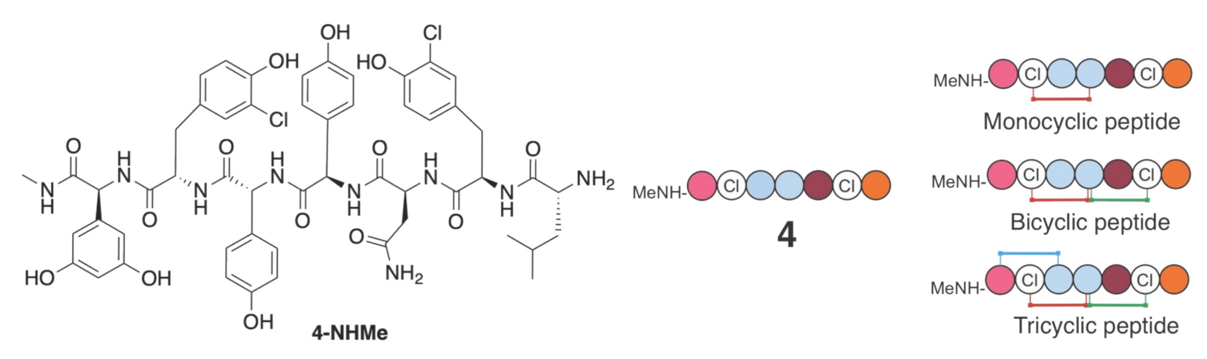

1.1 OxyB

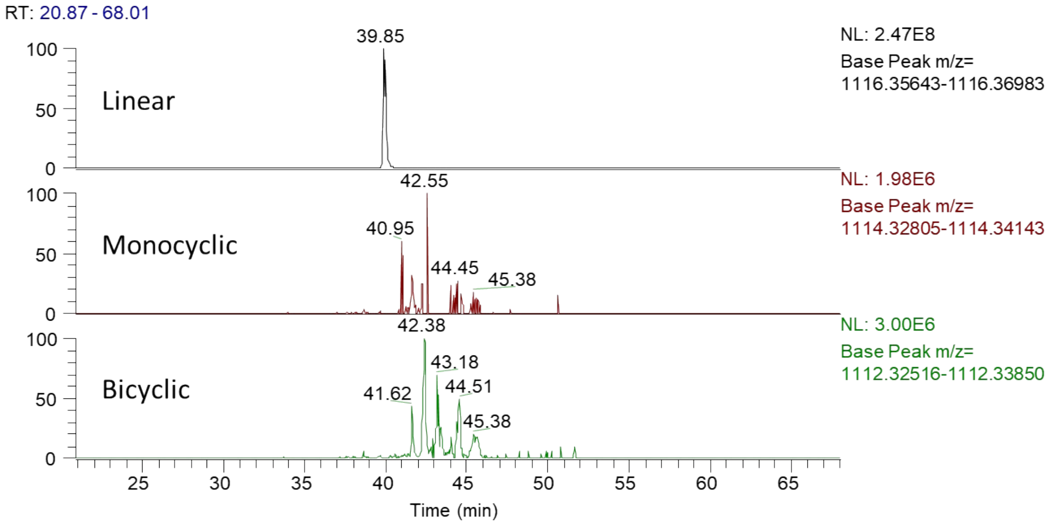

Figure S11. HPLC-HRMS analysis of the turnover of control 4 loaded on PCP-X_{tei} di-domain using OxyB. Extracted ion chromatograms for masses corresponding to the linear (black), monocyclic (red), and bicyclic (green) products (Orbitrap Fusion, $[M+H]^+$).

1.2 OxyB and A

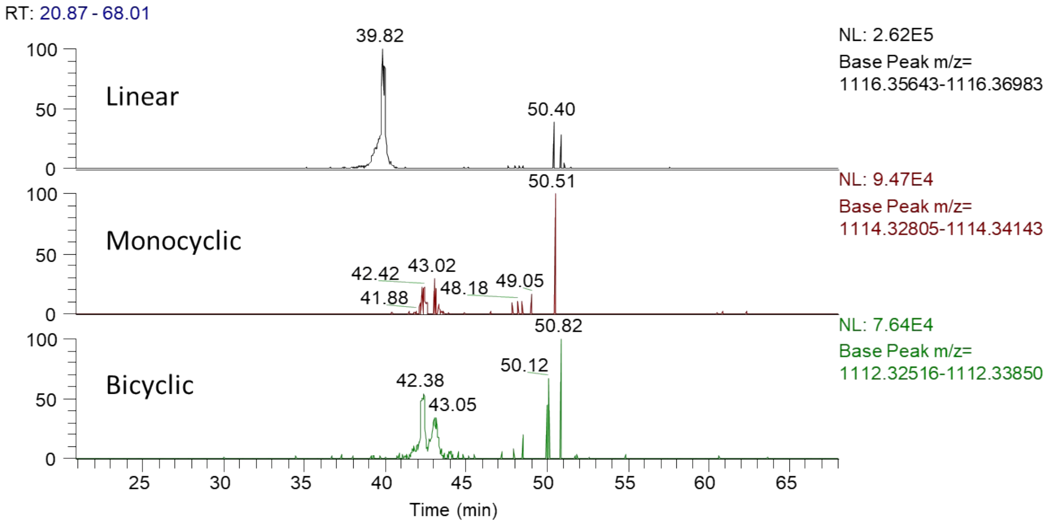

Figure S12. HPLC-HRMS analysis of the turnover of control 4 loaded on PCP-X$_{tei}$ di-domain using OxyB and A. Extracted ion chromatograms for masses corresponding to the linear (black), monocyclic (red), and bicyclic (green) products (Orbitrap Fusion, $[M+H]^+$).

1.3 OxyA-C

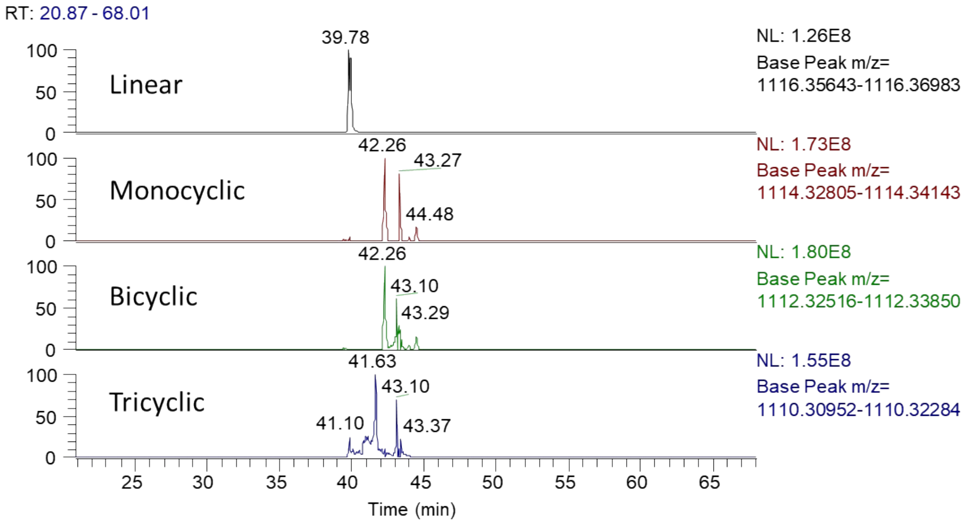

Figure S13. HPLC-HRMS analysis of the turnover of control 4 loaded on PCP-X$_{tei}$ di-domain using OxyA-C. Extracted ion chromatograms for masses corresponding to the linear (black), monocyclic (red), bicyclic (green), and tricyclic (blue) products (Orbitrap Fusion, $[M+H]^+$).
These compounds were characterised as previously reported.[10]

2 Turnover of PCP-X$_{tei}$ 5

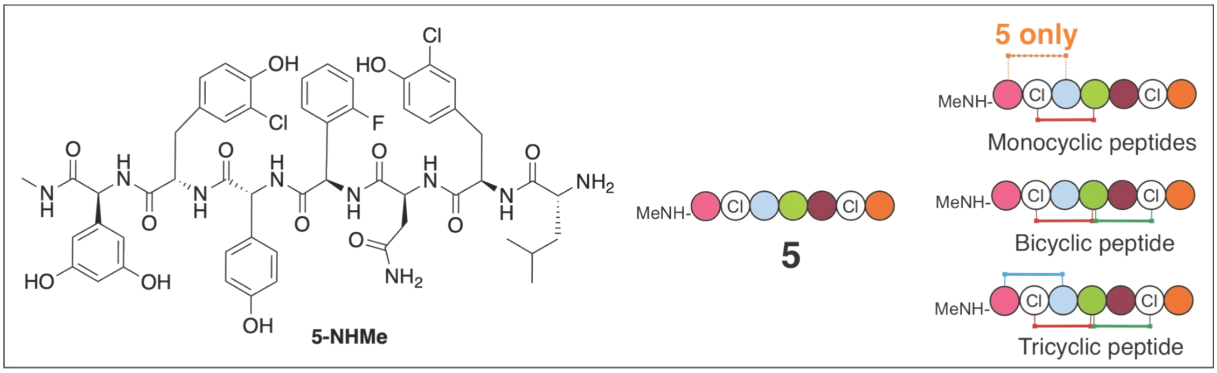

2.1 OxyB

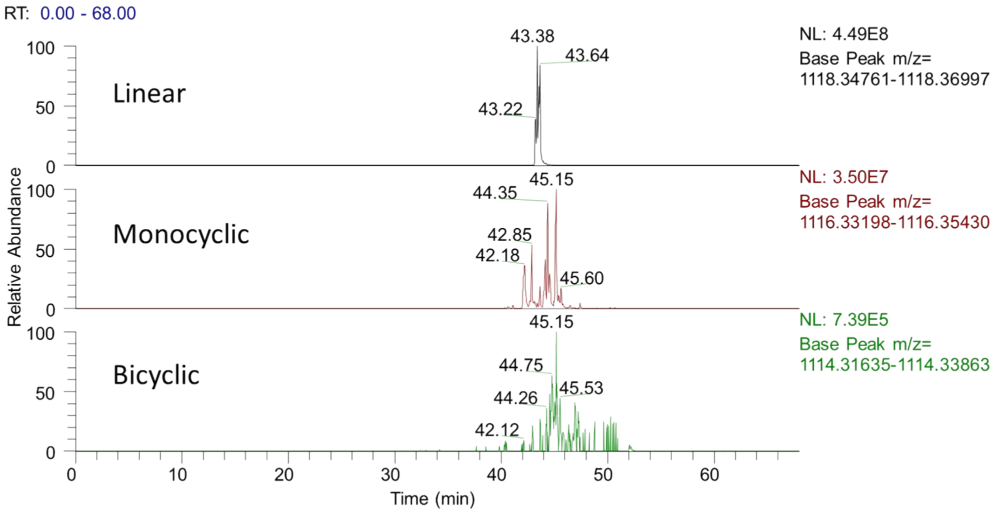

Figure S14. HPLC-HRMS analysis of the turnover of 5 loaded on PCP-X$_{tei}$ di-domain using OxyB. Extracted ion chromatograms for masses corresponding to the linear (black), monocyclic (red), and bicyclic (green) products (Orbitrap Fusion, $[M+H]^+$).

2.2 OxyB and A

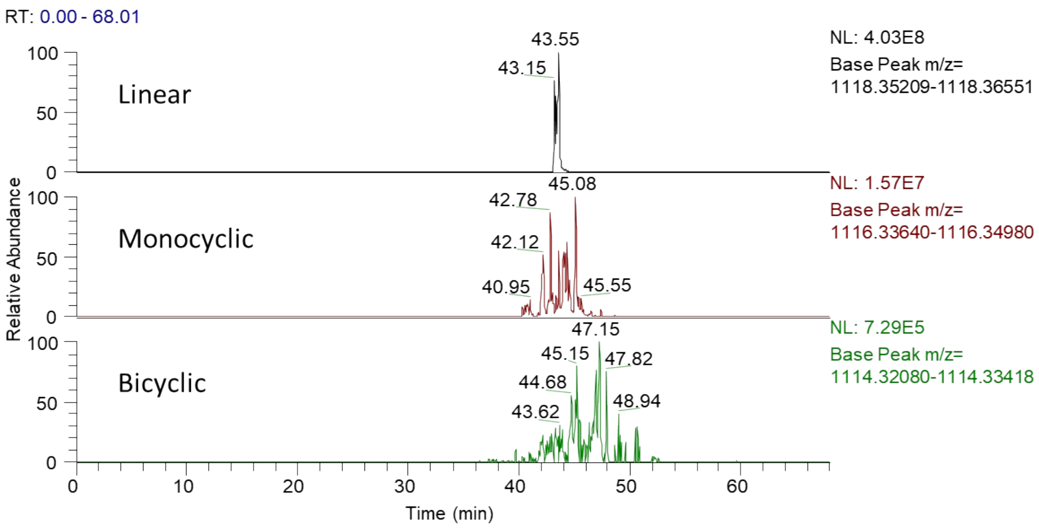

Figure S15. HPLC-HRMS analysis of the turnover of 5 loaded on PCP-X_{tei} di-domain using OxyB and A. Extracted ion chromatograms for masses corresponding to the linear (black), monocyclic (red), and bicyclic (green) products (Orbitrap Fusion, $[M+H]^+$).

2.3 OxyA-C

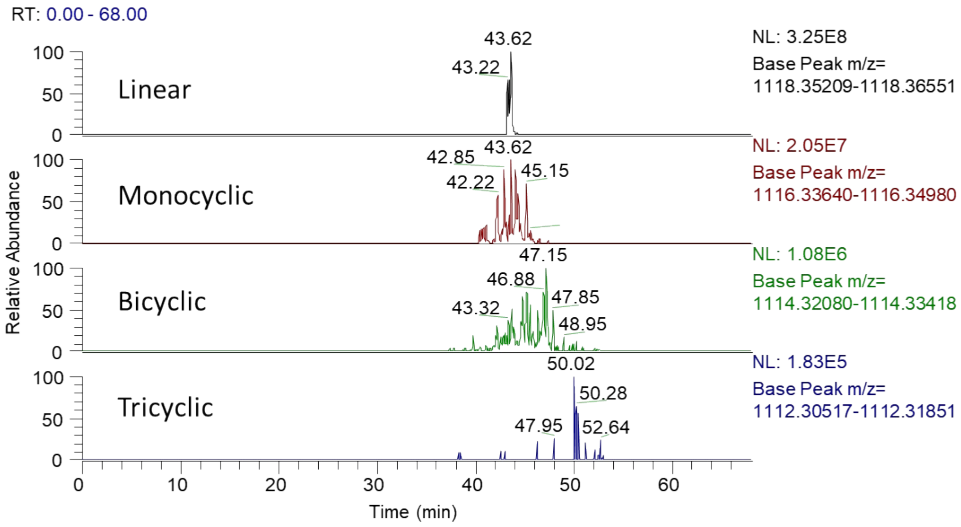

Figure S16. HPLC-HRMS analysis of the turnover of 5 loaded on PCP-X_{tei} di-domain using OxyA-C. Extracted ion chromatograms for masses corresponding to the linear (black), monocyclic (red), bicyclic (green), and tricyclic (blue) products (Orbitrap Fusion, $[M+H]^+$).

2.4 Linear compound characterisation

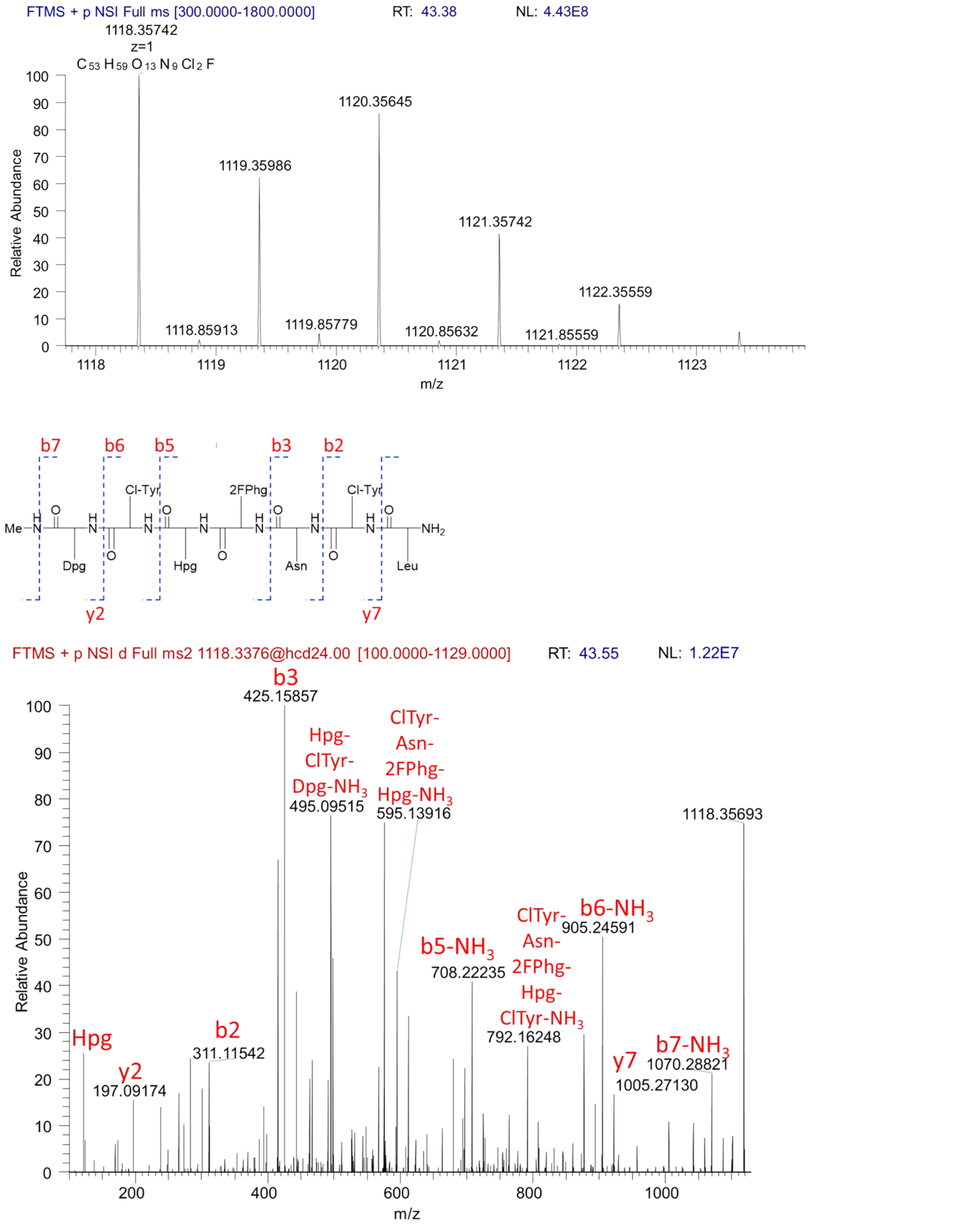

Figure S17.HPLC-HRMS analyses for the cleaved linear product from the turnover of 5. Top: Accurate mass and isotopic distribution of linear product. Bottom: Tabulated MS^2 fragmentation.

2.5 Monocyclic compound characterisation

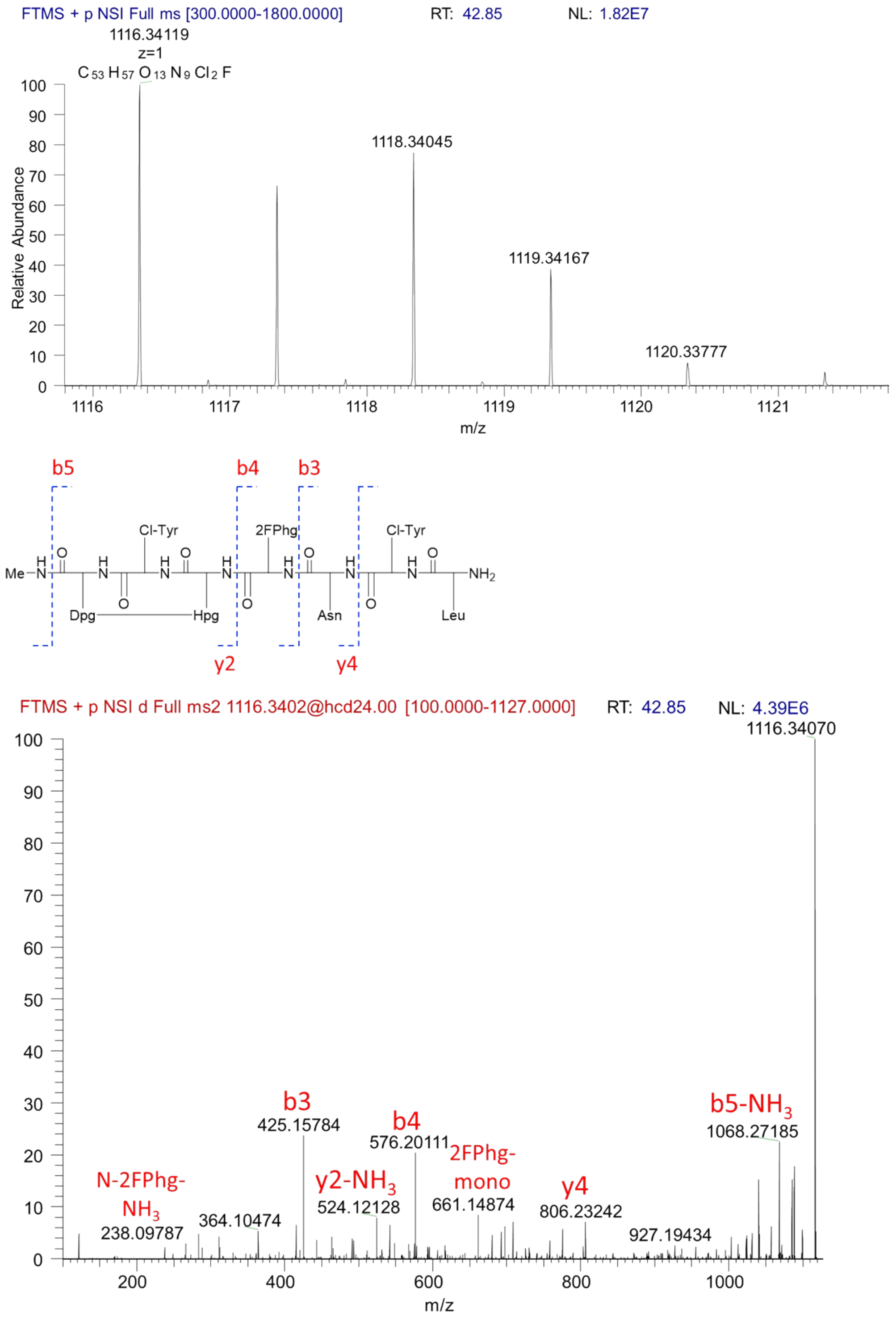

Figure S18. HPLC-HRMS analyses for the first (AB ring) cleaved monocyclic product from the turnover of 5. Top: Accurate mass and isotopic distribution of monocyclic product. Bottom: Tabulated MS[2] fragmentation.

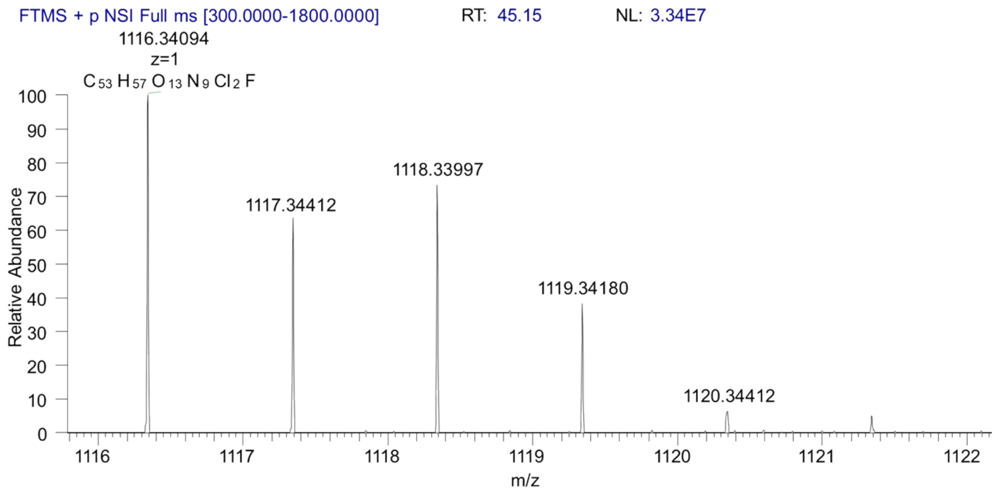

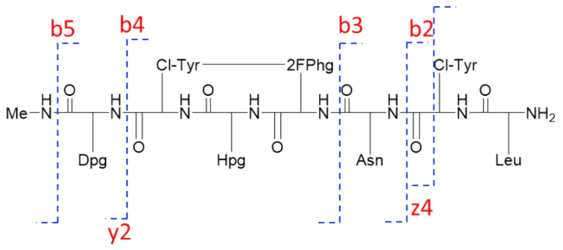

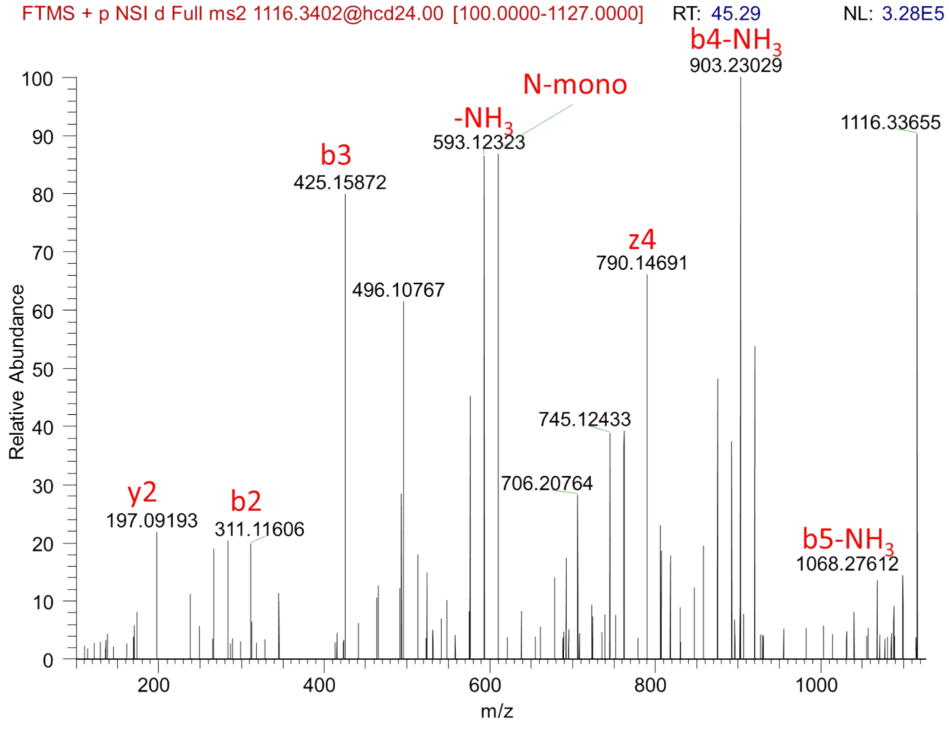

Figure S19. HPLC-HRMS analyses for the second (CD ring) cleaved monocyclic product from the turnover of 5. Top: Accurate mass and isotopic distribution of monocyclic product. Bottom: Tabulated MS^2 fragmentation.

2.6 Bicyclic compound characterisation

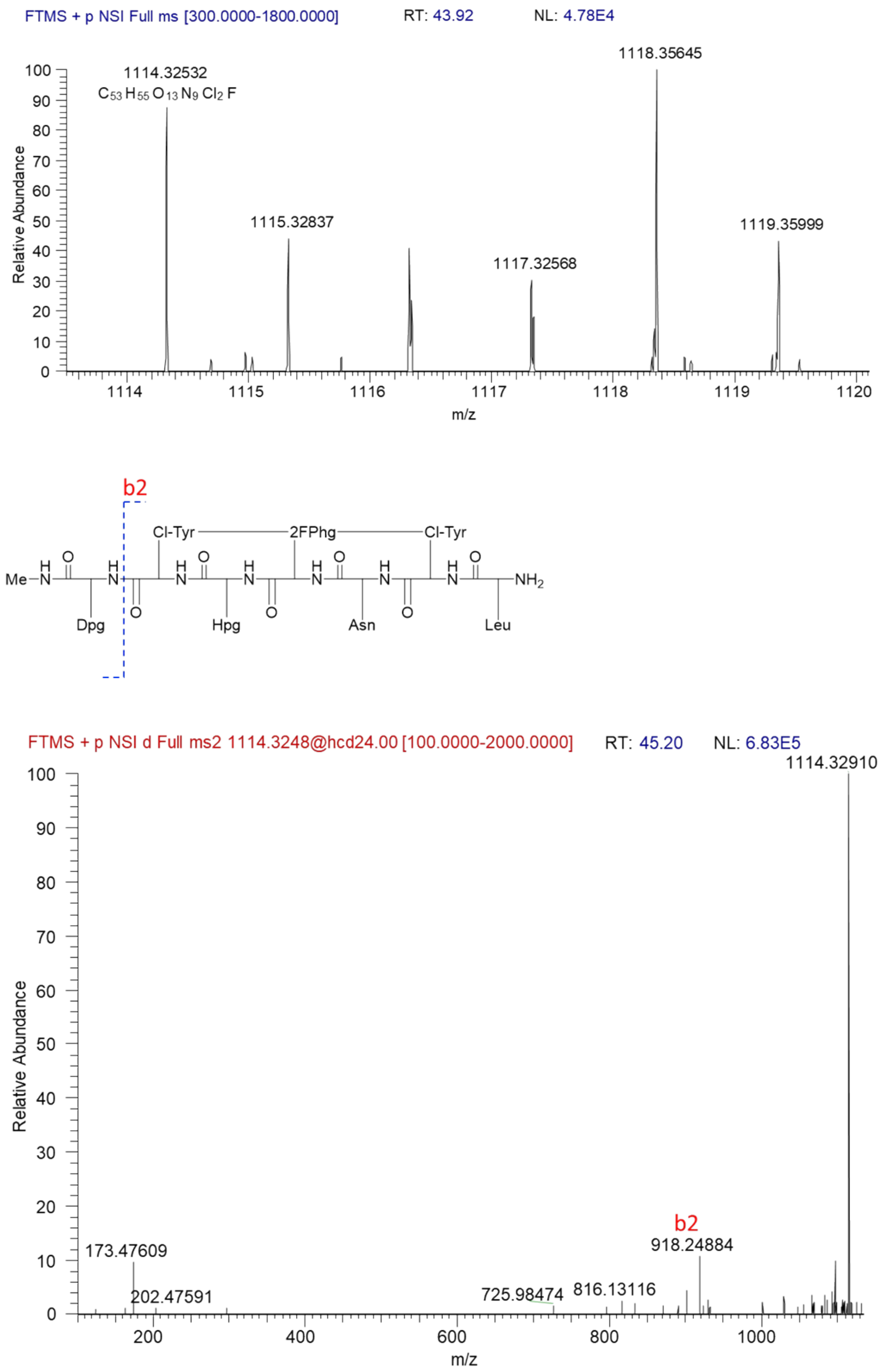

Figure S20. HPLC-HRMS analyses for the cleaved bicyclic product from the turnover of 5. Top: Accurate mass and isotopic distribution of bicyclic product. Bottom: Tabulated MS^2 fragmentation.

2.7 Tricyclic compound characterisation

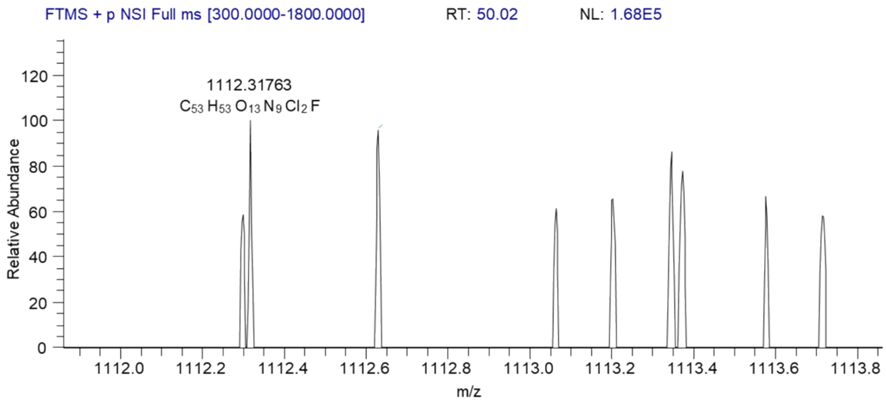

Figure S21. HPLC-HRMS analyses for the cleaved tricyclic product from the turnover of 5. Accurate mass and isotopic distribution of tricyclic product.

3 Turnover of PCP-X_{tei} 6

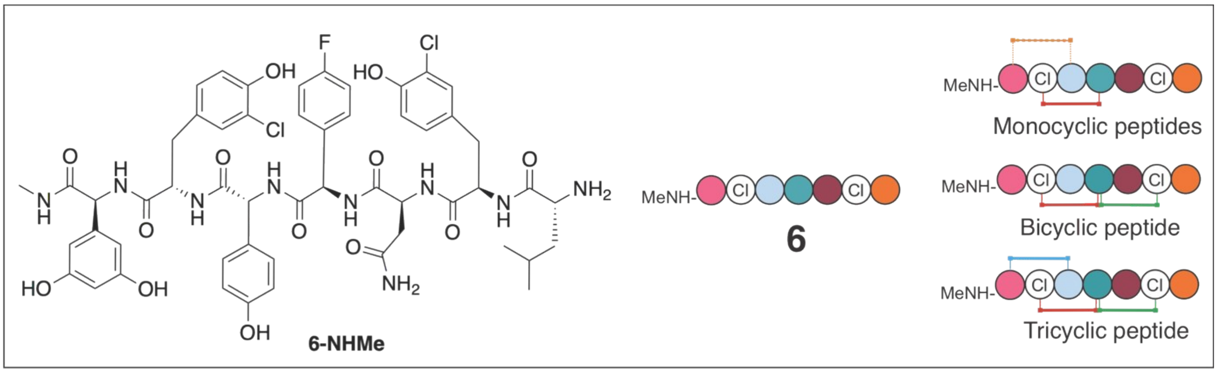

3.1 OxyB

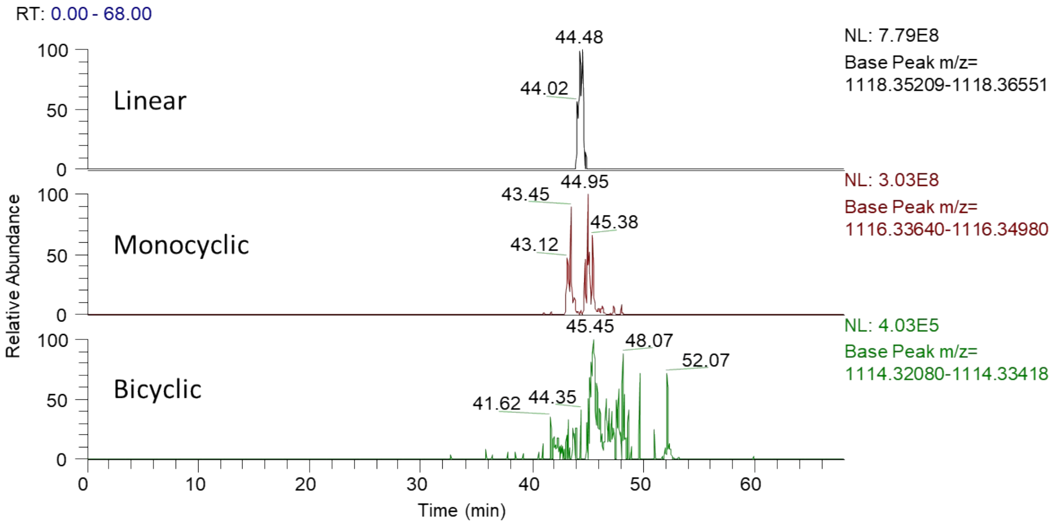

Figure S22. HPLC-HRMS analysis of the turnover of 6 loaded on PCP-X_{tei} di-domain using OxyB. Extracted ion chromatograms for masses corresponding to the linear (black), monocyclic (red), and bicyclic (green) products (Orbitrap Fusion, $[M+H]^+$).

3.2 OxyB and A

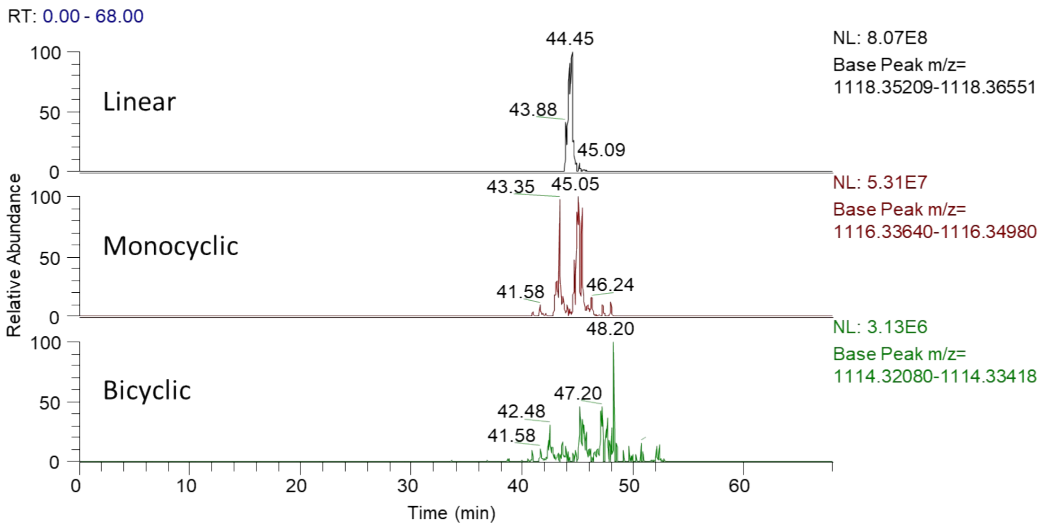

Figure S23. HPLC-HRMS analysis of the turnover of 6 loaded on PCP-X$_{tei}$ di-domain using OxyB and A. Extracted ion chromatograms for masses corresponding to the linear (black), monocyclic (red), and bicyclic (green) products (Orbitrap Fusion, $[M+H]^+$).

3.3 OxyA-C

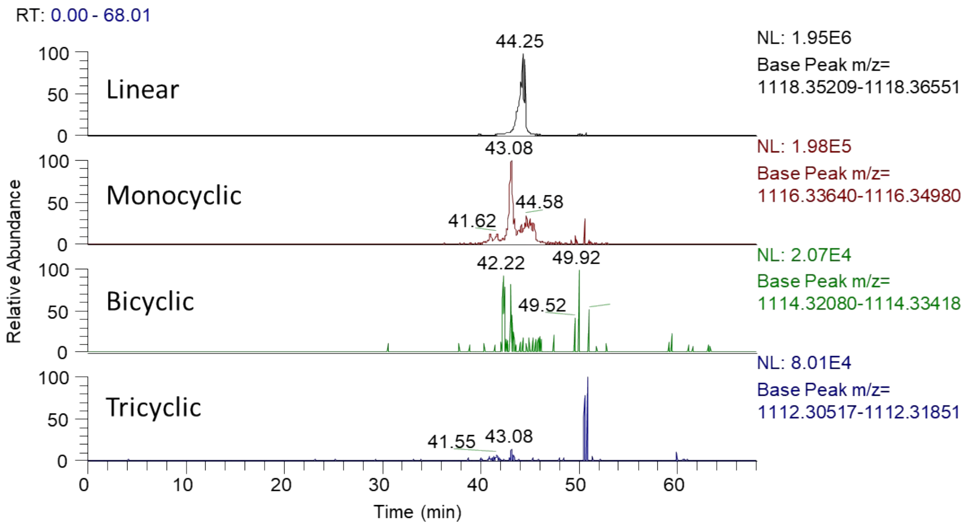

Figure S24. HPLC-HRMS analysis of the turnover of 6 loaded on PCP-X$_{tei}$ di-domain using OxyA-C. Extracted ion chromatograms for masses corresponding to the linear (black), monocyclic (red), bicyclic (green), and tricyclic (blue) products (Orbitrap Fusion, $[M+H]^+$).

3.4 Linear compound characterisation

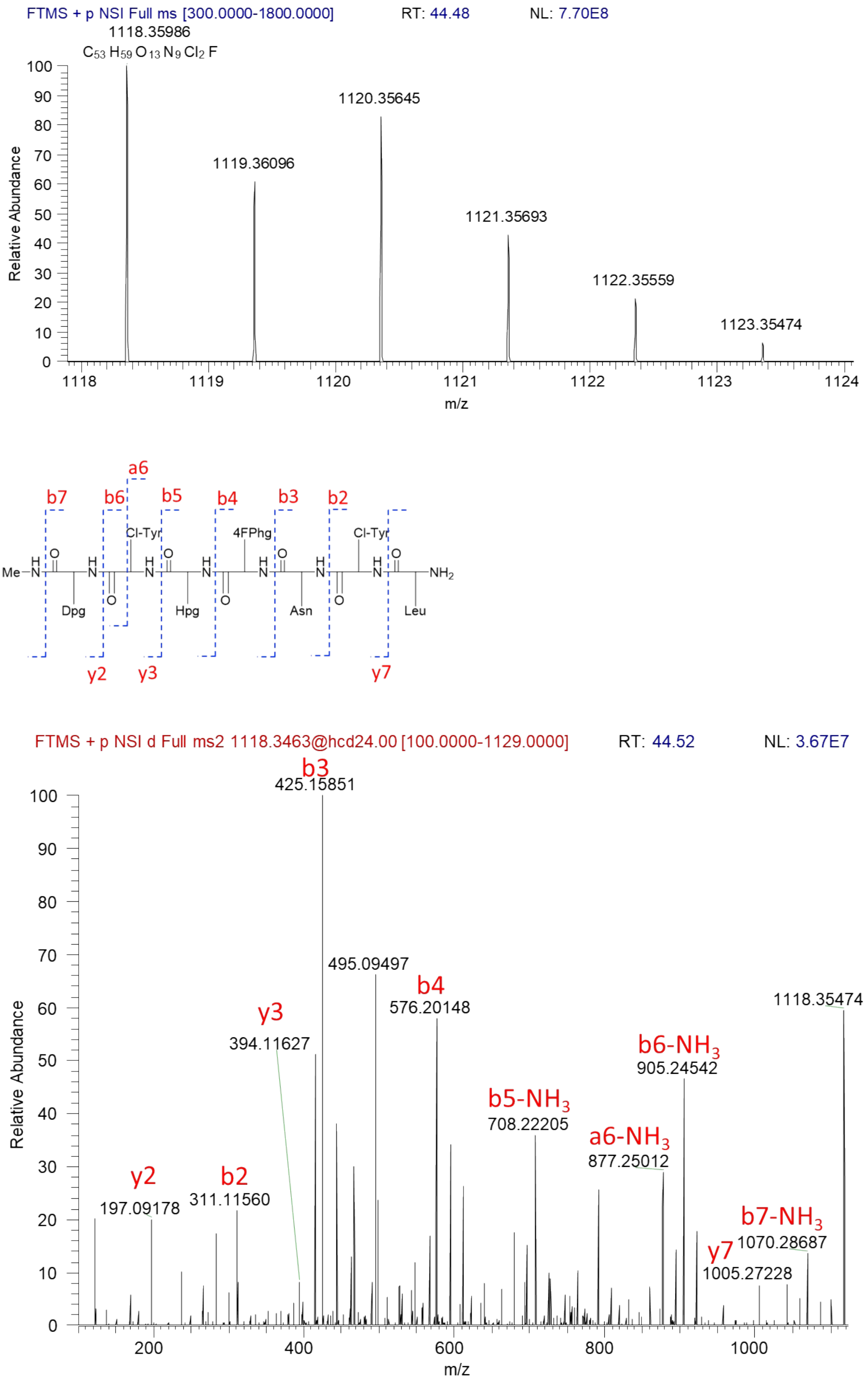

Figure S25. HPLC-HRMS analyses for the cleaved linear product from the turnover of 6. Top: Accurate mass and isotopic distribution of linear product. Bottom: Tabulated MS[2] fragmentation.

3.5 Monocyclic compound characterisation

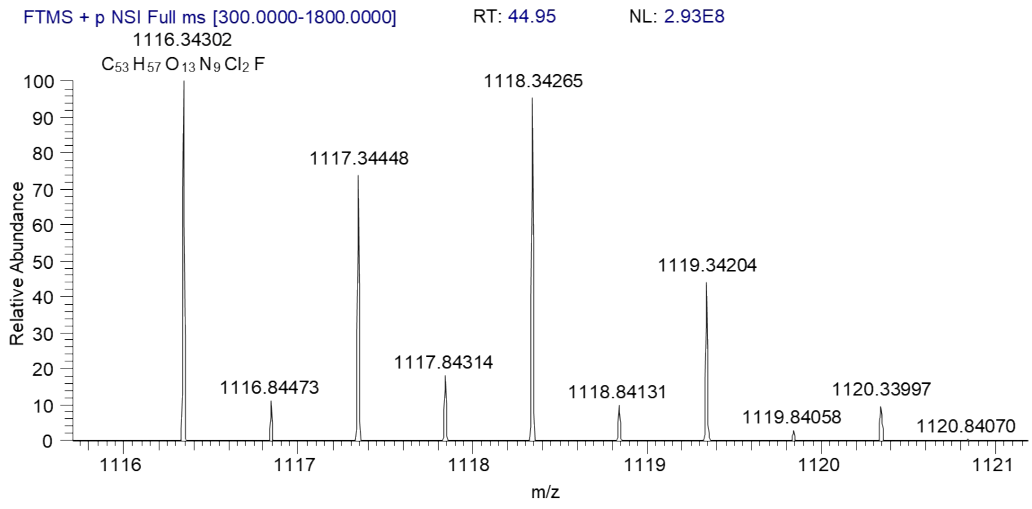

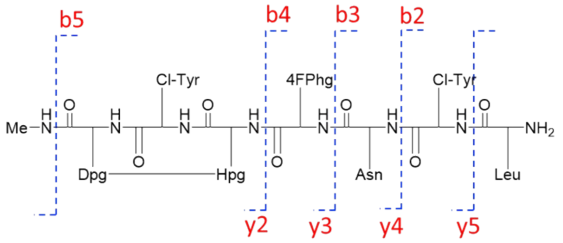

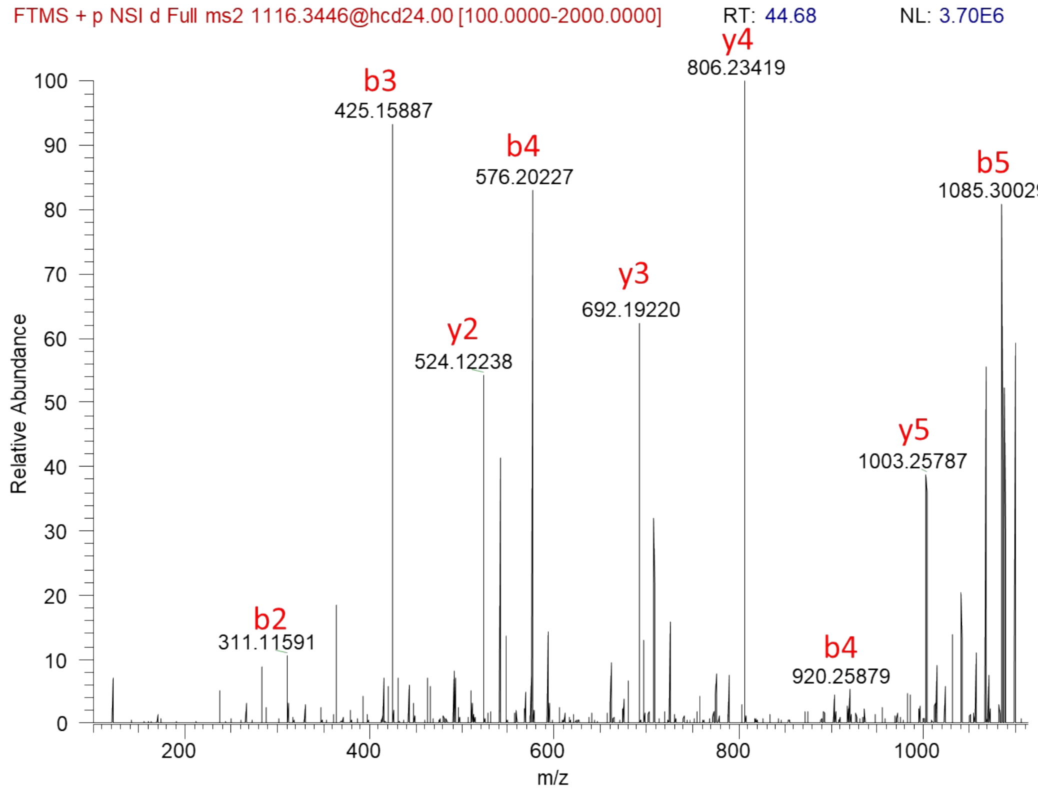

Figure S26. HPLC-HRMS analyses for the cleaved (AB ring) monocyclic product from the turnover of 6. Top: Accurate mass and isotopic distribution of monocyclic product. Bottom: Tabulated MS[2] fragmentation.

3.6 Bicyclic compound characterisation

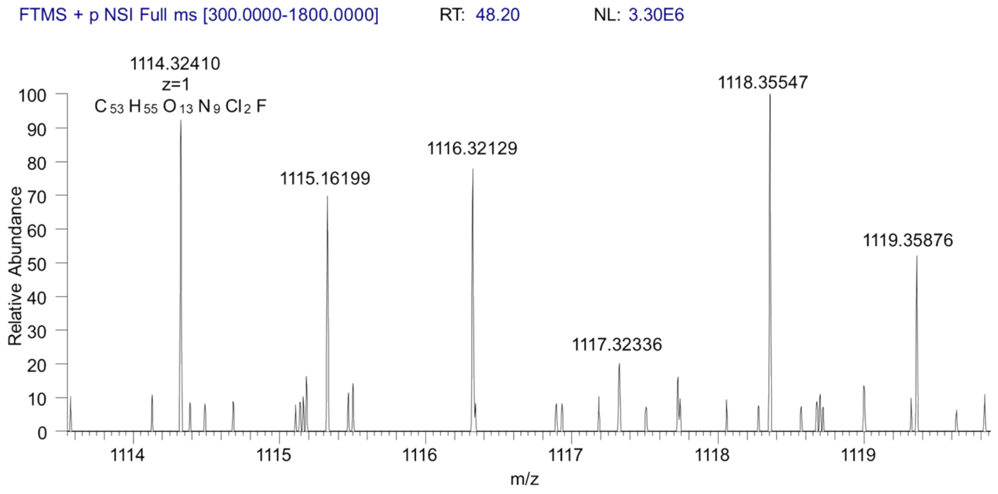

Figure S27. HPLC-HRMS analyses for the cleaved bicyclic product from the turnover of 6. Accurate mass and isotopic distribution of bicyclic product.

3.7 Tricyclic compound characterisation

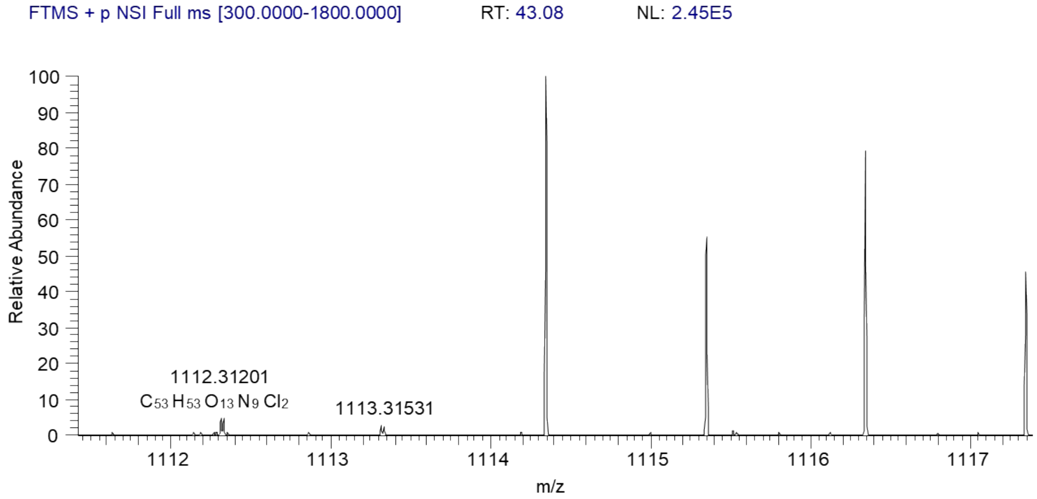

Figure S28. HPLC-HRMS analyses for the cleaved tricyclic product from the turnover of 6. Accurate mass and isotopic distribution of tricyclic product.

4 Turnover of PCP-X_{tei} 7

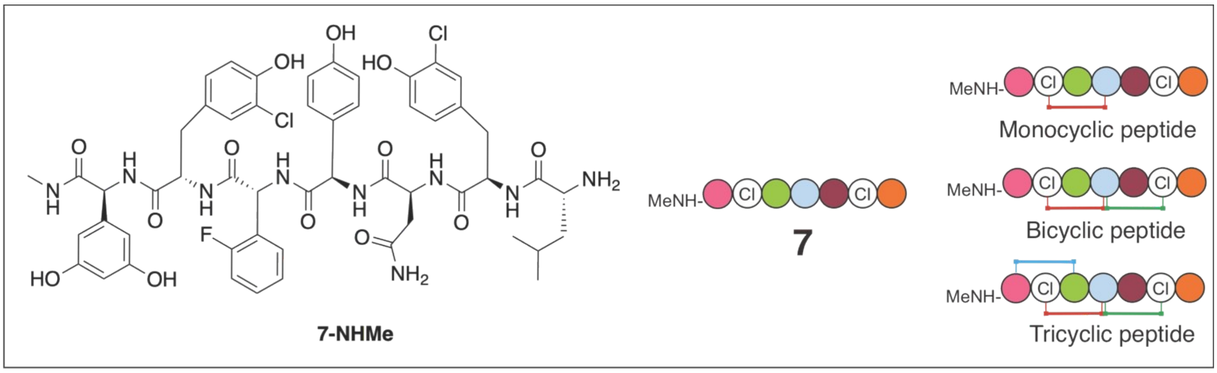

4.1 OxyB

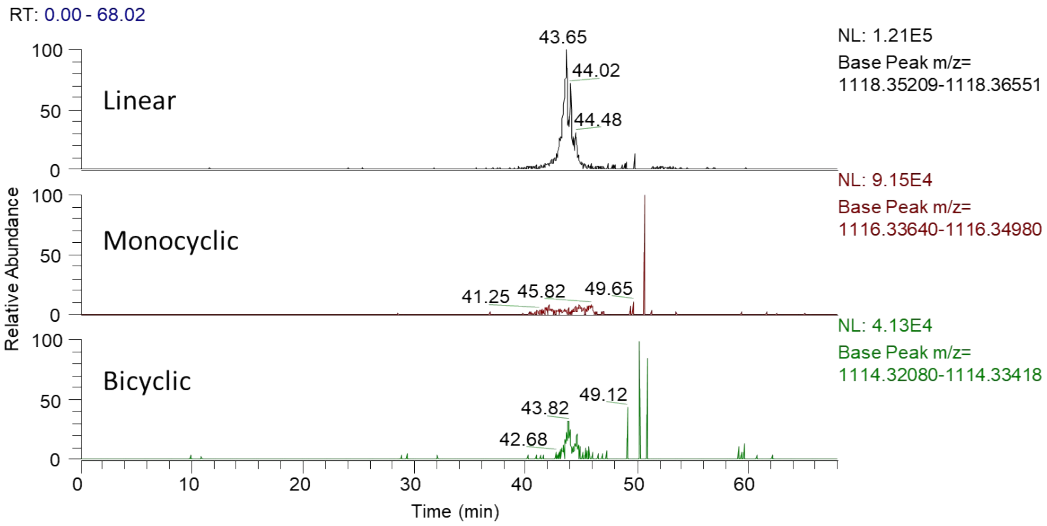

Figure S29. HPLC-HRMS analysis of the turnover of 7 loaded on PCP-X_{tei} di-domain using OxyB. Extracted ion chromatograms for masses corresponding to the linear (black), monocyclic (red), and bicyclic (green) products (Orbitrap Fusion, $[M+H]^+$).

4.2 OxyB and A

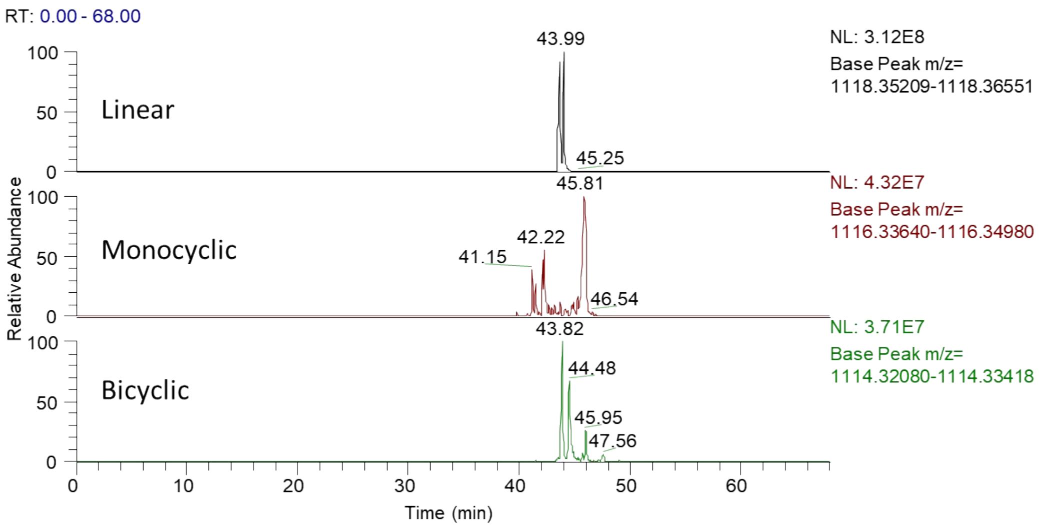

Figure S30. HPLC-HRMS analysis of the turnover of 7 loaded on PCP-X$_{tei}$ di-domain using OxyB and A. Extracted ion chromatograms for masses corresponding to the linear (black), monocyclic (red), and bicyclic (green) products (Orbitrap Fusion, $[M+H]^+$).

4.3 OxyA-C

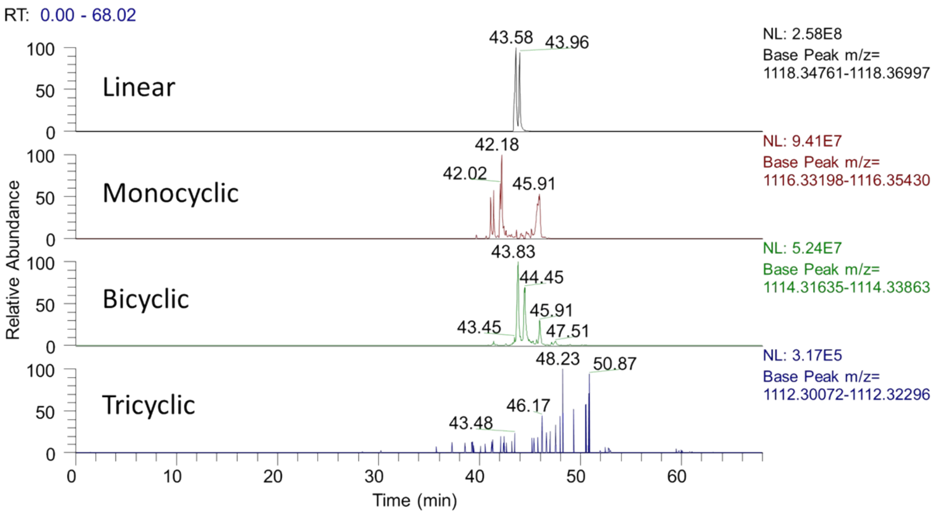

Figure S31. HPLC-HRMS analysis of the turnover of 7 loaded on PCP-X$_{tei}$ di-domain using OxyA-C. Extracted ion chromatograms for masses corresponding to the linear (black), monocyclic (red), bicyclic (green), and tricyclic (blue) products (Orbitrap Fusion, $[M+H]^+$).

4.4 Linear compound characterisation

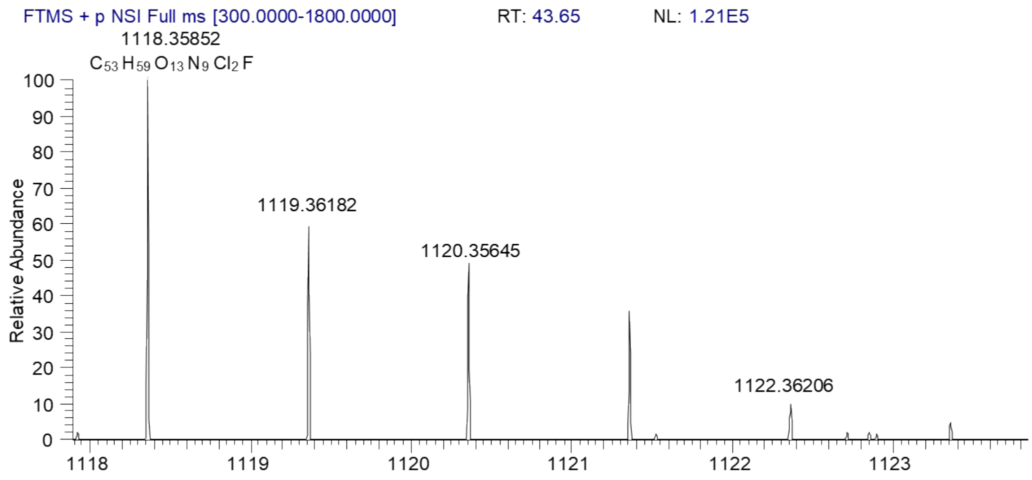

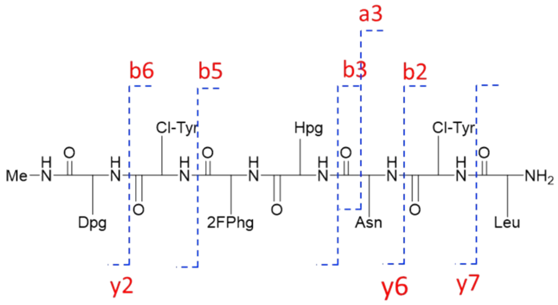

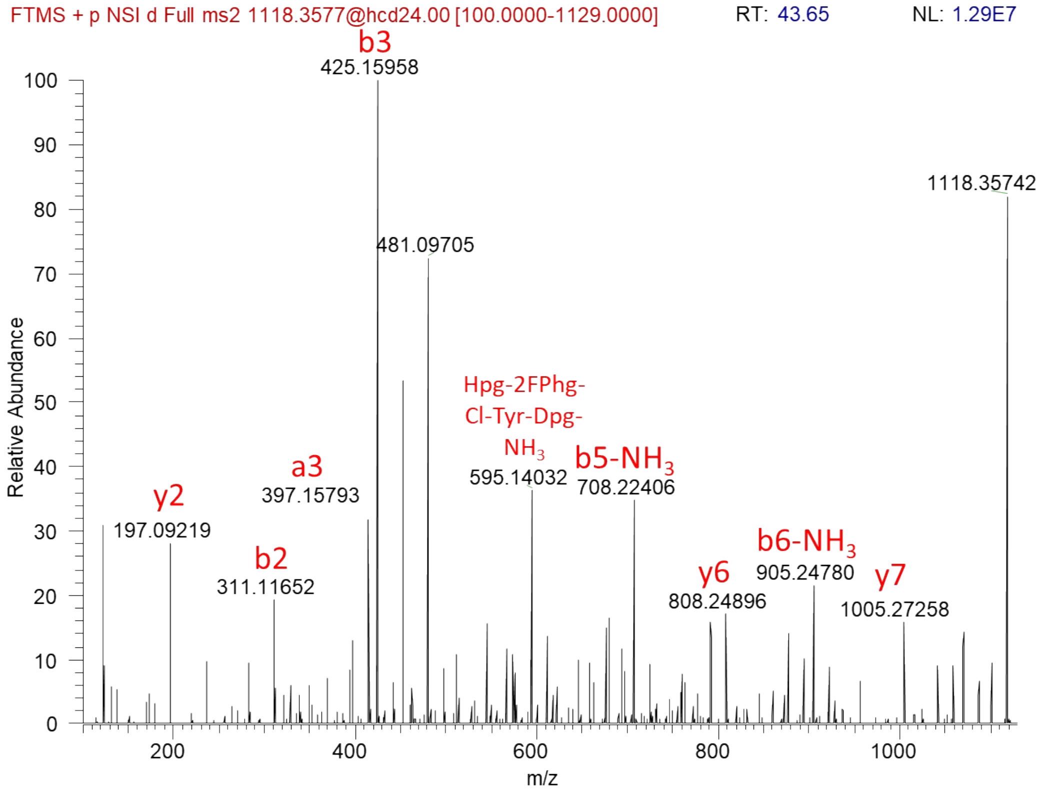

Figure S32. HPLC-HRMS analyses for the cleaved linear product from the turnover of 7. Top: Accurate mass and isotopic distribution of linear product. Bottom: Tabulated MS[2] fragmentation.

4.5 Monocyclic compound characterisation

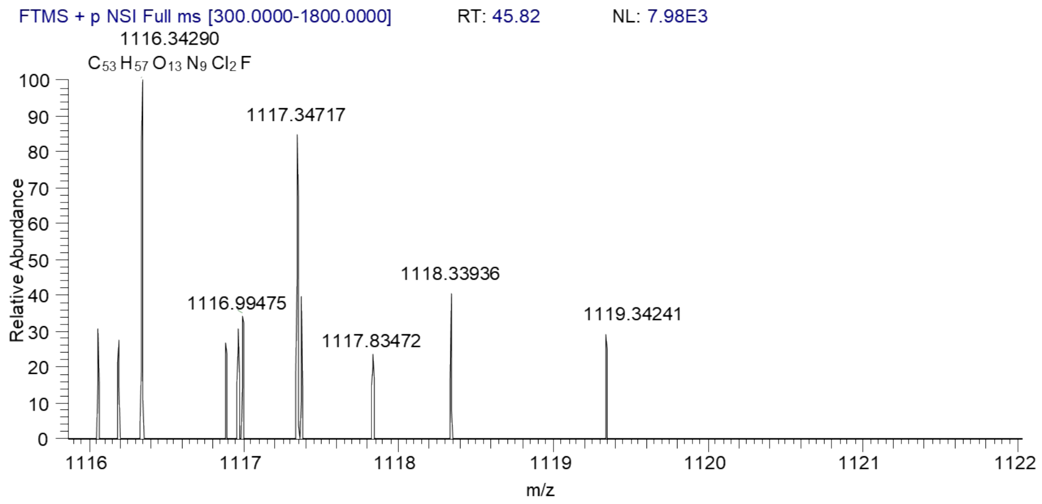

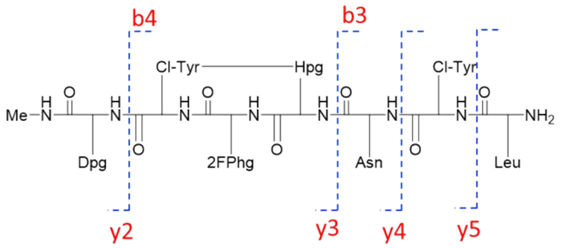

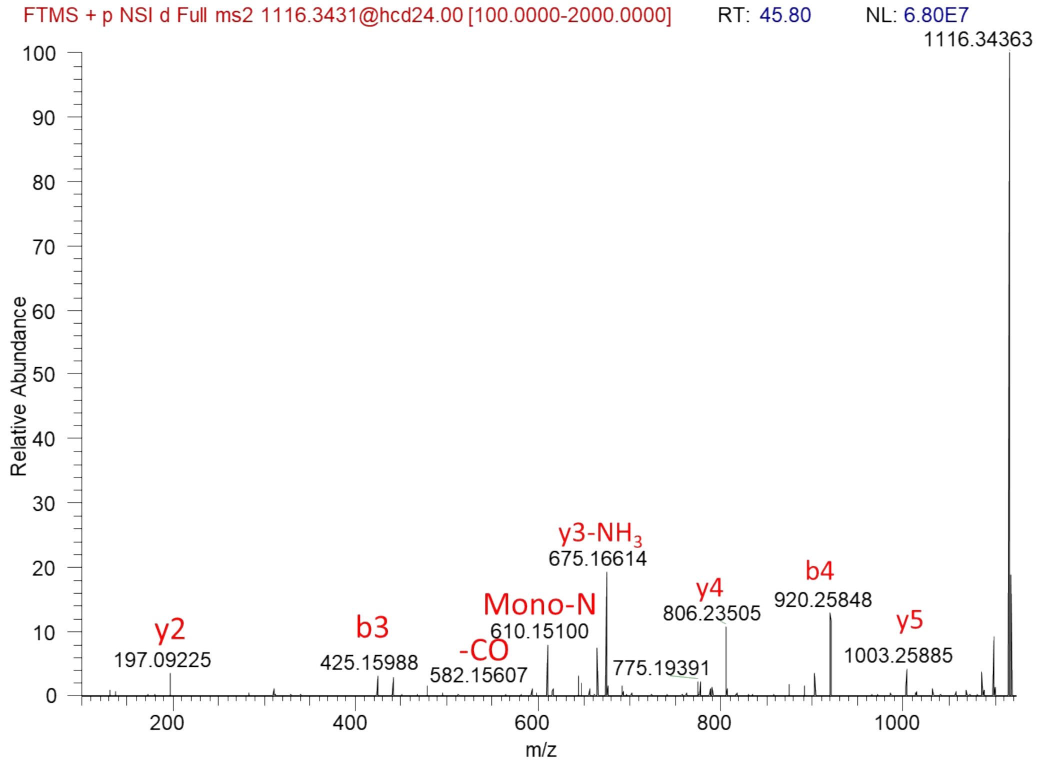

Figure S33. HPLC-HRMS analyses for the cleaved (CD ring) monocyclic product from the turnover of 7. Top: Accurate mass and isotopic distribution of monocyclic product. Bottom: Tabulated MS^2 fragmentation.

4.6 Bicyclic compound characterisation

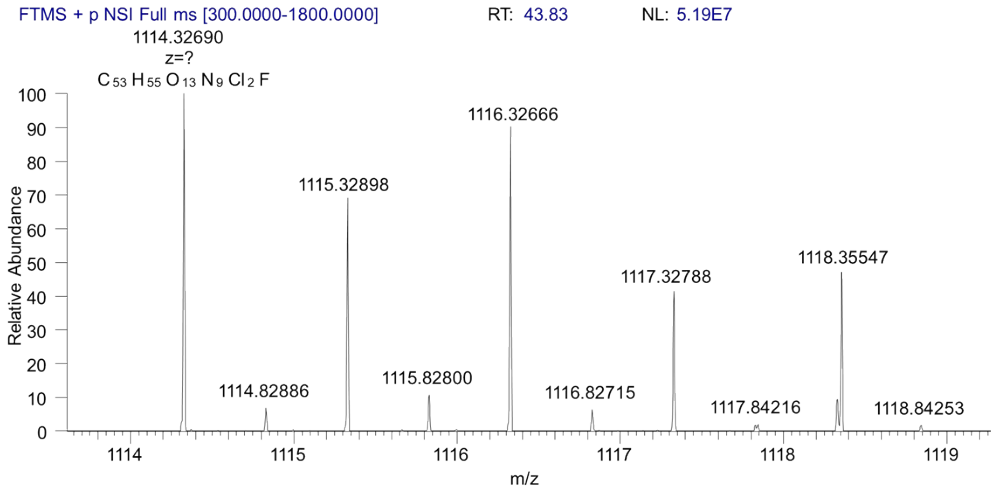

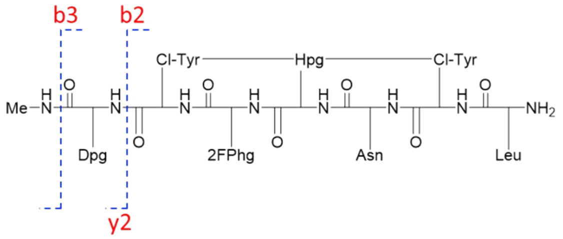

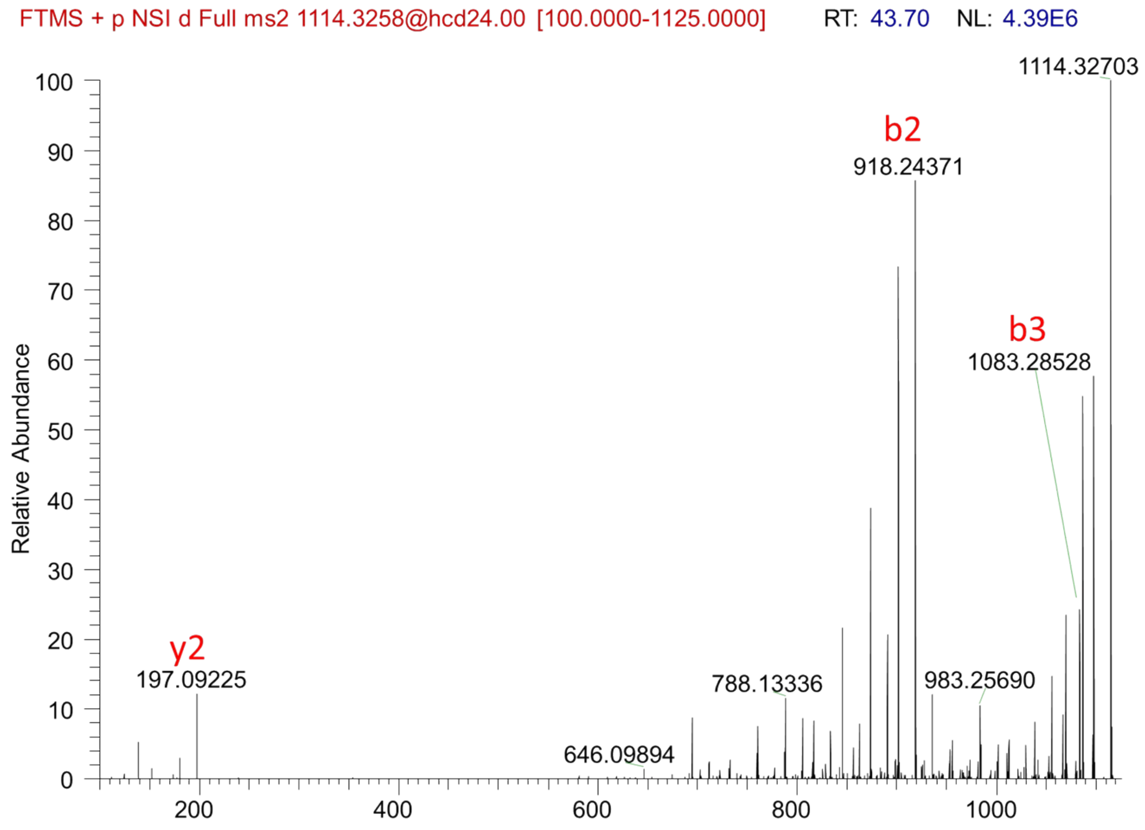

Figure S34. HPLC-HRMS analyses for the cleaved bicyclic product from the turnover of 7. Top: Accurate mass and isotopic distribution of bicyclic product. Bottom: Tabulated MS[2] fragmentation.

4.7 Tricyclic compound characterisation

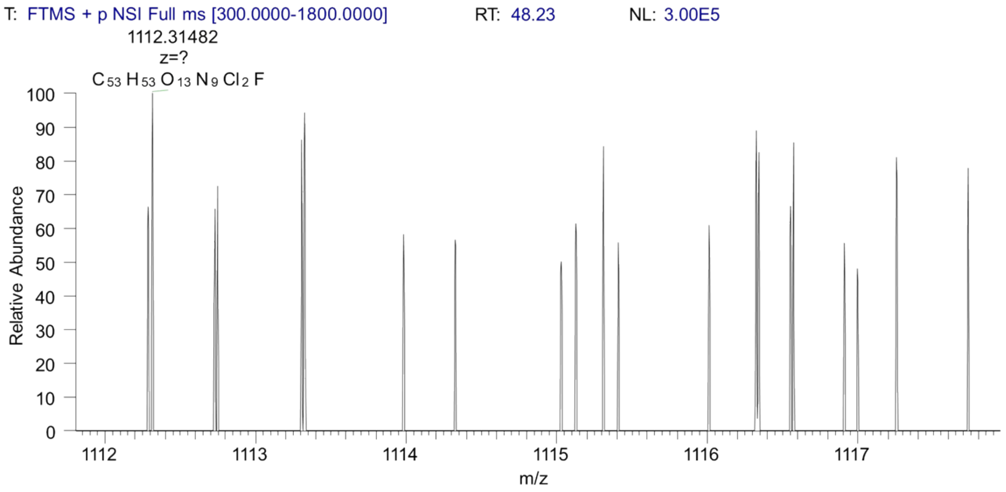

Figure S35. HPLC-HRMS analyses for the cleaved tricyclic product from the turnover of 7. Accurate mass and isotopic distribution of tricyclic product.

5 Turnover of PCP-X_{tei} 8

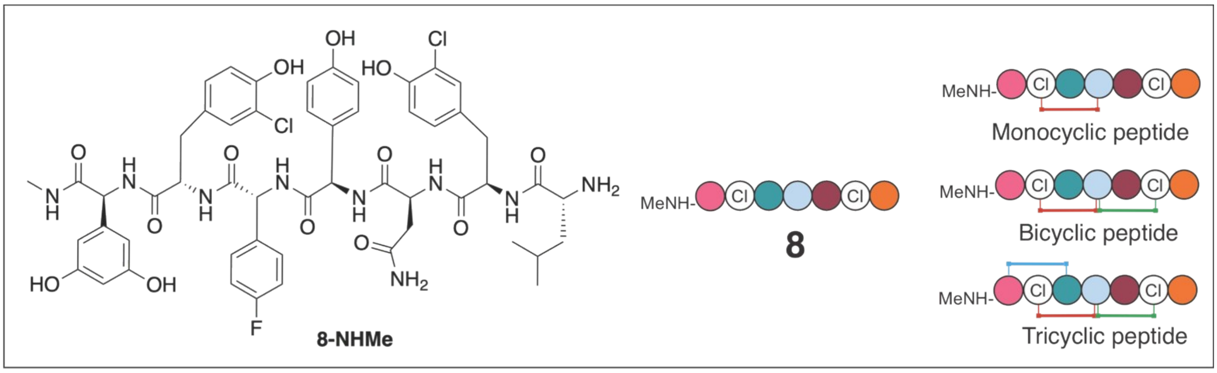

5.1 OxyB

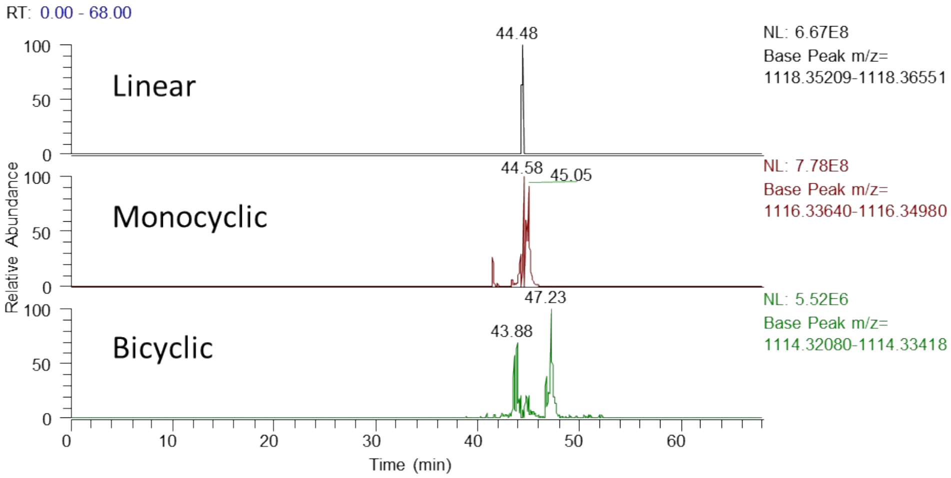

Figure S36. HPLC-HRMS analysis of the turnover of 8 loaded on PCP-X_{tei} di-domain using OxyB. Extracted ion chromatograms for masses corresponding to the linear (black), monocyclic (red), and bicyclic (green) products (Orbitrap Fusion, $[M+H]^+$).

5.2 OxyB and A

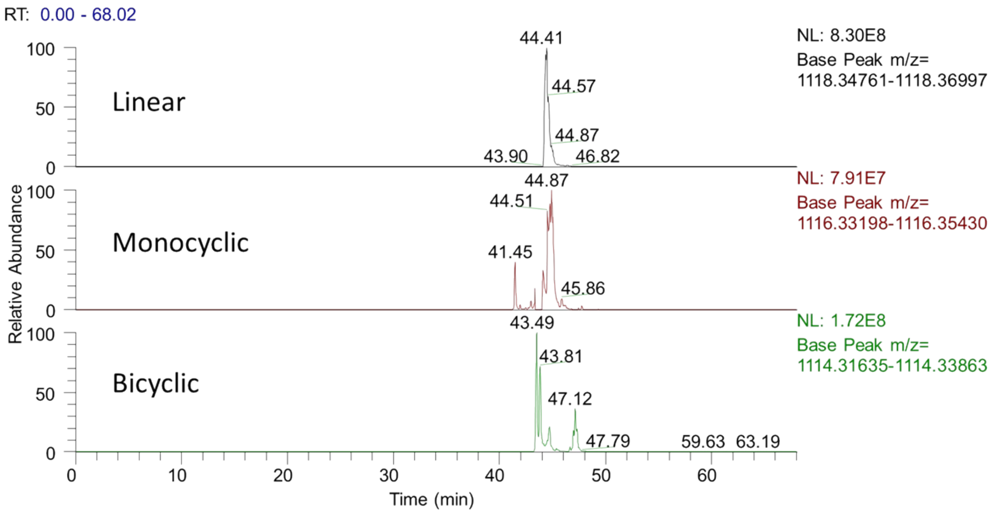

Figure S37. HPLC-HRMS analysis of the turnover of 8 loaded on PCP-X_{tei} di-domain using OxyB and A. Extracted ion chromatograms for masses corresponding to the linear (black), monocyclic (red), and bicyclic (green) products (Orbitrap Fusion, $[M+H]^+$).

5.3 OxyA-C

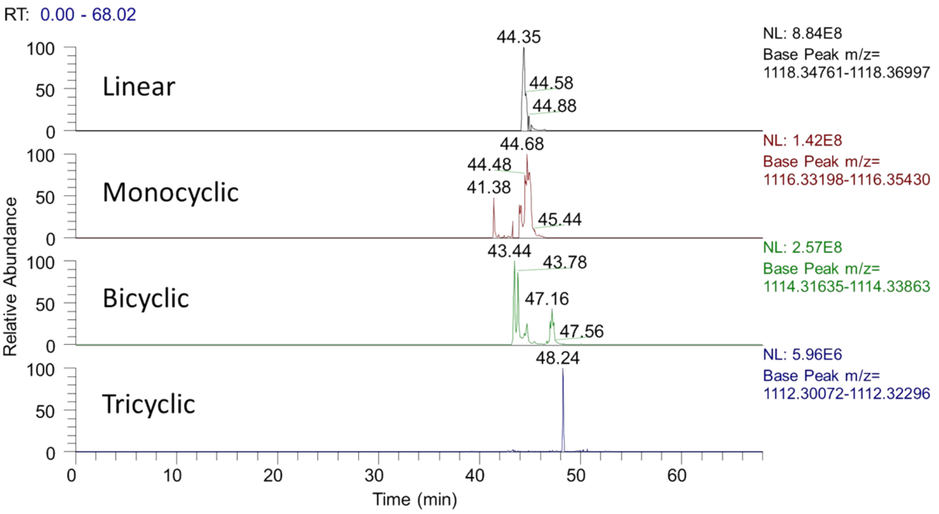

Figure S38. HPLC-HRMS analysis of the turnover of 8 loaded on PCP-X_{tei} di-domain using OxyA-C. Extracted ion chromatograms for masses corresponding to the linear (black), monocyclic (red), bicyclic (green), and tricyclic (blue) products (Orbitrap Fusion, $[M+H]^+$).

5.4 Linear compound characterisation

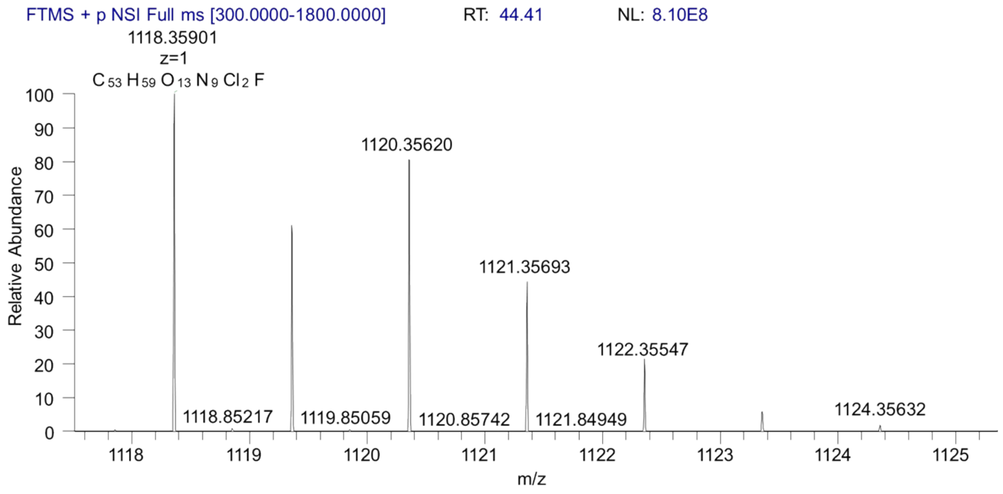

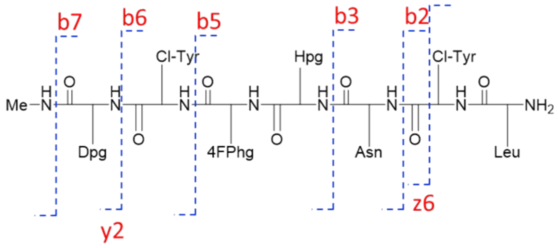

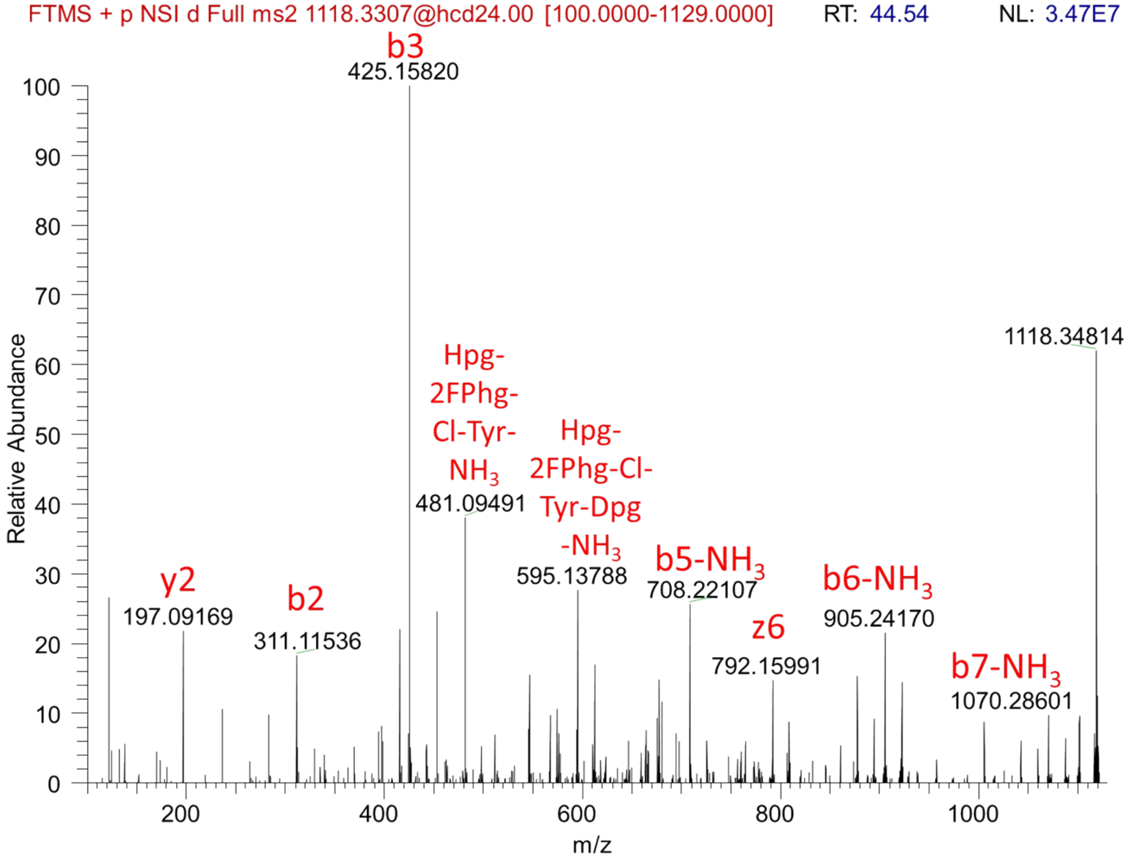

Figure S39. HPLC-HRMS analyses for the cleaved linear product from the turnover of 8. Top: Accurate mass and isotopic distribution of linear product. Bottom: Tabulated MS[2] fragmentation.

5.5 Monocyclic compound characterisation

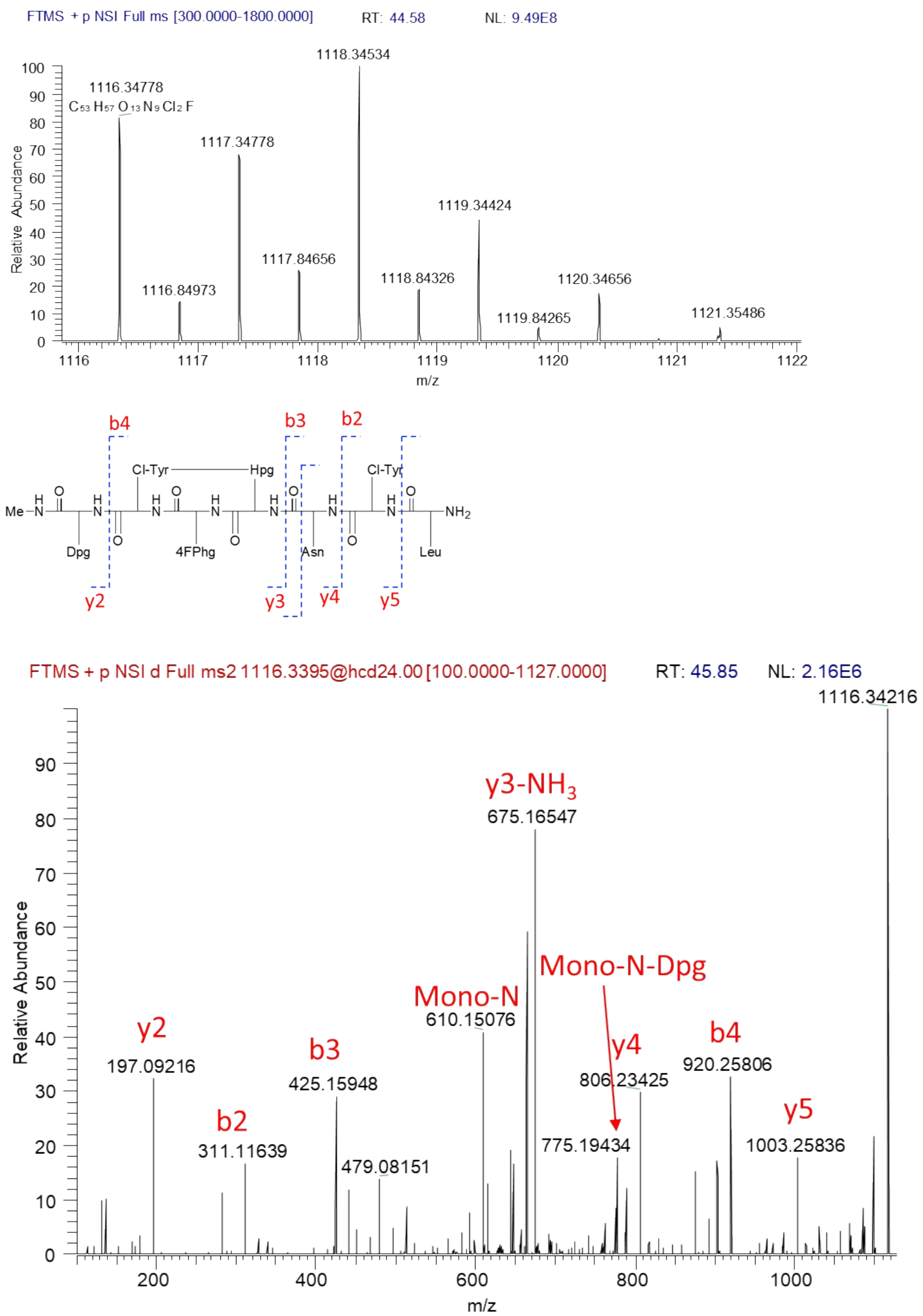

Figure S40. HPLC-HRMS analyses for the cleaved (CD ring) monocyclic product from the turnover of 8. Top: Accurate mass and isotopic distribution of monocyclic product. Bottom: Tabulated MS^2 fragmentation.

5.6 Bicyclic compound characterisation

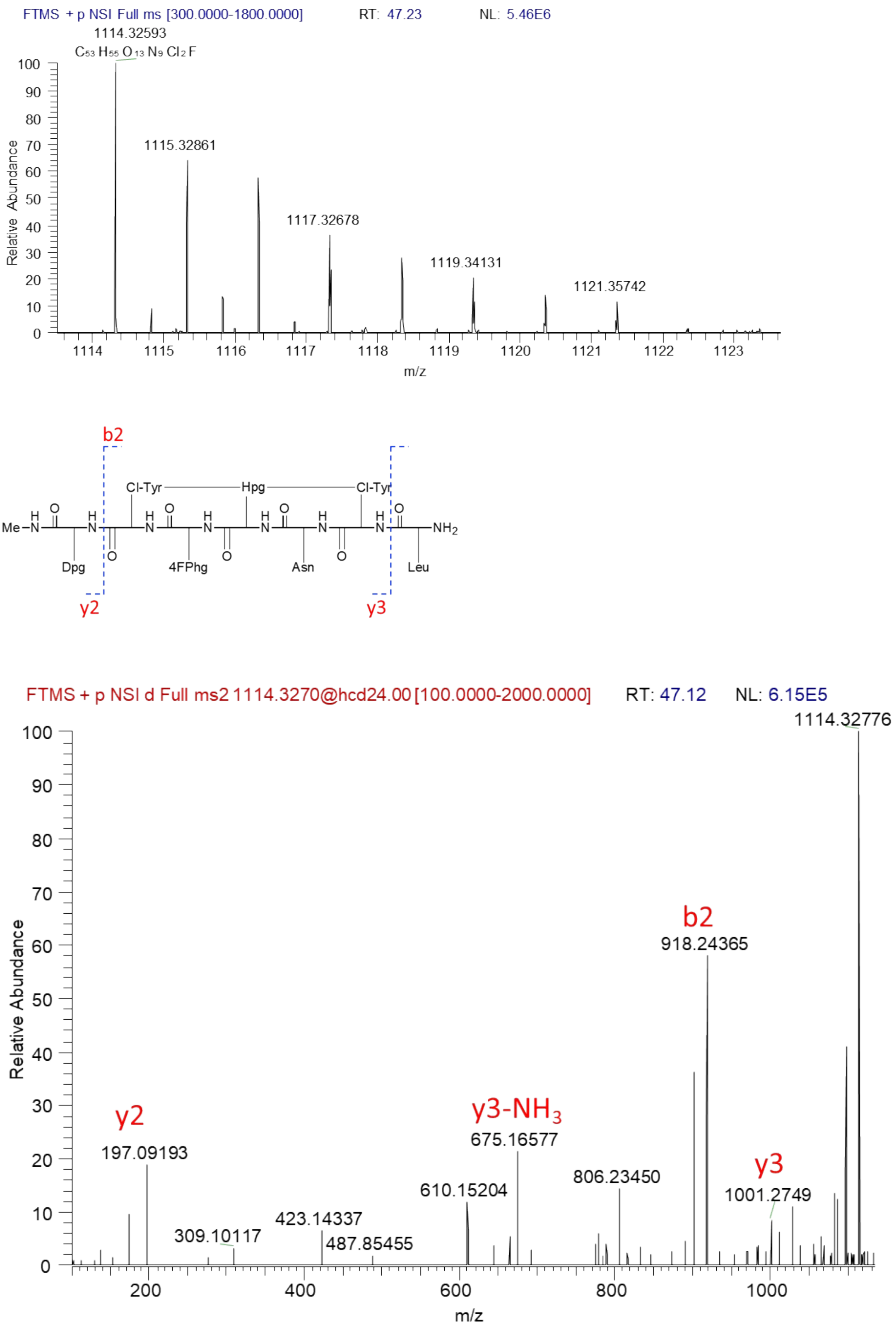

Figure S41. HPLC-HRMS analyses for the cleaved bicyclic product from the turnover of 8. Top: Accurate mass and isotopic distribution of bicyclic product. Bottom: Tabulated MS[2] fragmentation.

5.7 Tricyclic compound characterisation

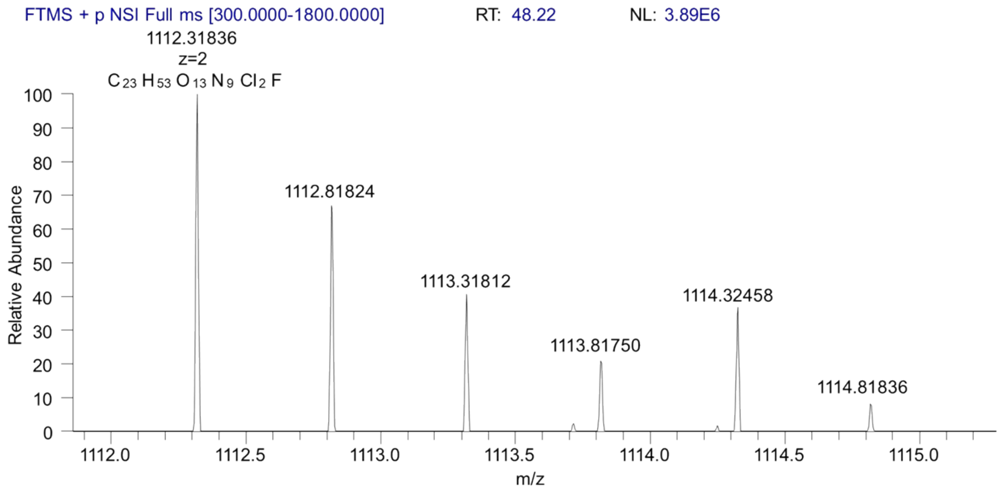

Figure S42. HPLC-HRMS analyses for the cleaved tricyclic product from the turnover of 8. Accurate mass and isotopic distribution of tricyclic product.

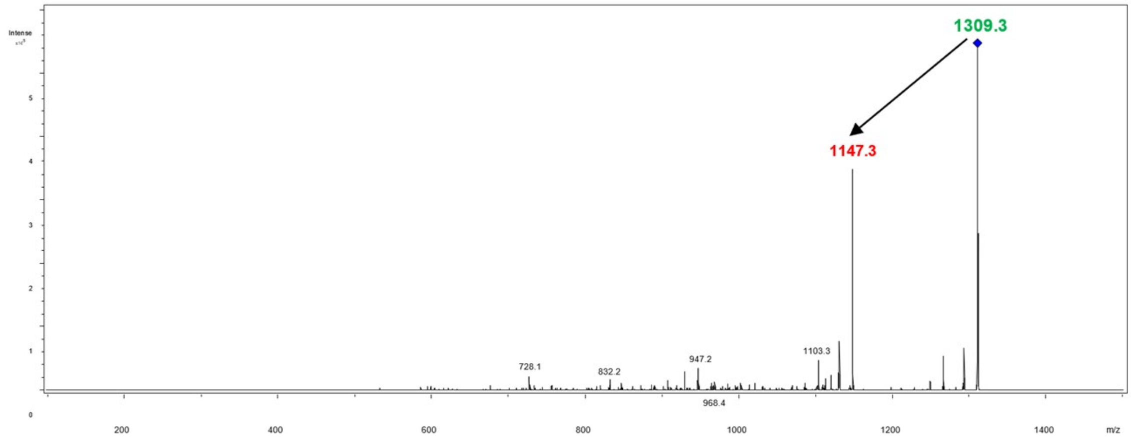

Figure S43. MS[2] fragmentation pattern of new fluorinated balhimycin derivative. Mass with *m/z* 1309.3 $[M+H]^+$ obtained after feeding of *A. balhimycina* Δ*hpg* with both 2-fluoro-DL-phenylglycine and 4-hydroxy-L-phenylglycine (marked with green) as the glycosylated and tri-cyclized heptapeptide with mass signal of 1147.3 $[M+H]^+$ (marked with red) indicating the loss of D-glucose sugar. The black arrow indicates the steps of fragmentation of targeted mass.

Mass Spectrum SmartFormula Report

Analysis Info

Analysis Name	E:\Iriana\Mirobiol_Irina_IV-Fluorobalh_GE6_01_46221.d	Acquisition Date	12/17/2020 8:57:57 PM
Method	automsms_50-1800_pos_spez.fraglist_xx_0.3.m	Operator	BDAL@DE
Sample Name	Mirobiol_Irina_IV-Fluorobalh	Instrument	maXis 288882.21253
Comment			

Acquisition Parameter

Source Type	ESI	Ion Polarity	Positive	Set Nebulizer	2.0 Bar
Focus	Not active	Set Capillary	4500 V	Set Dry Heater	200 °C
Scan Begin	50 m/z	Set End Plate Offset	-500 V	Set Dry Gas	8.0 l/min
Scan End	1800 m/z	Set Charging Voltage	0 V	Set Divert Valve	Waste
		Set Corona	0 nA	Set APCI Heater	0 °C

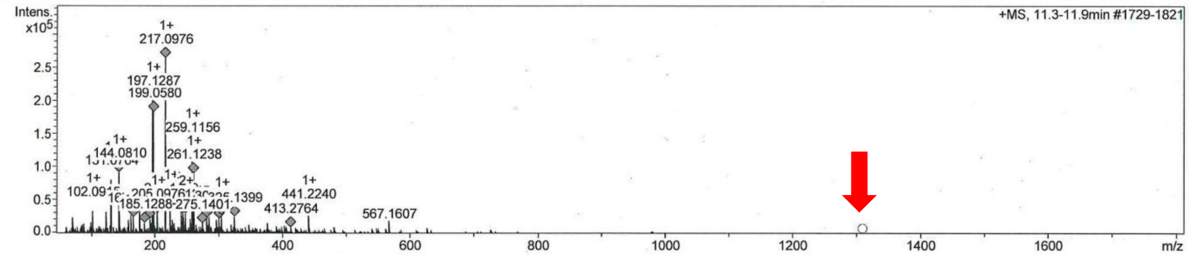

Meas. m/z	#	Ion Formula	m/z	err [ppm]	Mean err [ppm]	rdb	N-Rule	e⁻ Conf	mSigma	Std I	Std Mean m/z	Std I VarNorm	Std m/z Diff	Std Comb Dev
1309.357140	1	C59H64Cl2FN8O21	1309.354161	-2.3	-1.0	31.0	ok	even	124.8	114.3	n.a.	n.a.	n.a.	n.a.
	2	C60H60Cl2FN12O17	1309.355499	-1.3	-0.3	36.0	ok	even	127.3	115.7	n.a.	n.a.	n.a.	n.a.
	3	C63H68Cl2FN2O23	1309.356846	-0.2	0.6	30.0	ok	even	131.5	118.7	n.a.	n.a.	n.a.	n.a.
	4	C64H64Cl2FN6O19	1309.358184	0.8	1.3	35.0	ok	even	135.4	120.4	n.a.	n.a.	n.a.	n.a.
	5	C65H60Cl2FN10O15	1309.359521	1.8	2.0	40.0	ok	even	140.1	122.3	n.a.	n.a.	n.a.	n.a.

Mirobiol_Irina_IV-Fluorobalh_GE6_01_46221.d
Bruker Compass DataAnalysis 4.4 printed: 2/5/2021 2:34:04 PM by: MS Page 1 of 1

Figure S44. The mass spectrum from HR HPLC-MS analysis of concentrated culture supernatant from *A. balhimycina* Δ*hpg* supplemented with 2-fluoro-DL-phenylglycine and 4-hydroxy-L-phenylglycine. The mass indicated with the red arrow for *m/z* 1309.3541 $[M+H]^+$ corresponds to a fluorinated bicyclic heptapeptide with both a glucose moiety and an N-terminal methyl group with molecular formula $C_{59}H_{64}Cl_2FN_8O_{21}$.

Mass Spectrum SmartFormula Report

Analysis Info			
Analysis Name	F:\Daten\Maxis_2021\Stegmann\Irina\Irina_211020\Microbio_Irina_IV-4_GC1_01_53299.d	Acquisition Date	10/19/2021 10:55:43 PM
Method	automsms_50-1800_pos_spez.fraglist_xx_0.3.m	Operator	BDAL@DE
Sample Name	Microbio_Irina_IV-4	Instrument	maXis 288882.21253
Comment			

Acquisition Parameter

Source Type	ESI	Ion Polarity	Positive	Set Nebulizer	2.0 Bar
Focus	Not active	Set Capillary	4500 V	Set Dry Heater	200 °C
Scan Begin	50 m/z	Set End Plate Offset	-500 V	Set Dry Gas	8.0 l/min
Scan End	1800 m/z	Set Charging Voltage	0 V	Set Divert Valve	Waste
		Set Corona	0 nA	Set APCI Heater	0 °C

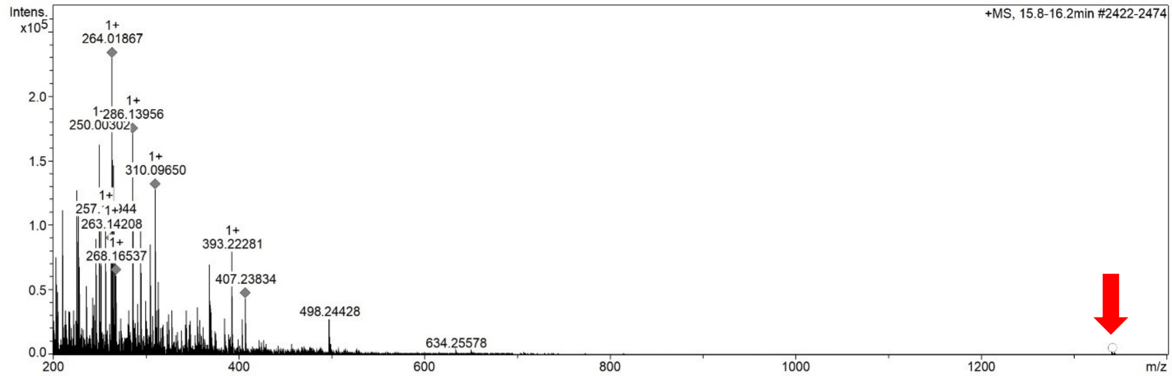

Meas. m/z	#	Ion Formula	m/z	err [ppm]	Mean err [ppm]	rdb	N-Rule	e⁻ Conf	mSigma	Std I	Std Mean m/z	Std I VarNorm	Std m/z Diff	Std Comb Dev
1341.32060	1	C59H61Cl2F2N8O22	1341.32400	2.54	1.74	32.0	ok	even	127.1	114.2	n.a.	n.a.	n.a.	n.a.

Microbio_Irina_IV-4_GC1_01_53299.d
Bruker Compass DataAnalysis 4.4 printed: 5/11/2023 3:09:03 PM by: MS Page 1 of 1

Display Report

Analysis Info			
Analysis Name	F:\Daten\Maxis_2021\Stegmann\Irina\Irina_211020\Microbio_Irina_IV-4_GC1_01_53299.d	Acquisition Date	10/19/2021 10:55:43 PM
Method	automsms_50-1800_pos_spez.fraglist_xx_0.3.m	Operator	BDAL@DE
Sample Name	Microbio_Irina_IV-4	Instrument	maXis 288882.21253
Comment			

Acquisition Parameter

Source Type	ESI	Ion Polarity	Positive	Set Nebulizer	2.0 Bar
Focus	Not active	Set Capillary	4500 V	Set Dry Heater	200 °C
Scan Begin	50 m/z	Set End Plate Offset	-500 V	Set Dry Gas	8.0 l/min
Scan End	1800 m/z	Set Charging Voltage	0 V	Set Divert Valve	Waste
		Set Corona	0 nA	Set APCI Heater	0 °C

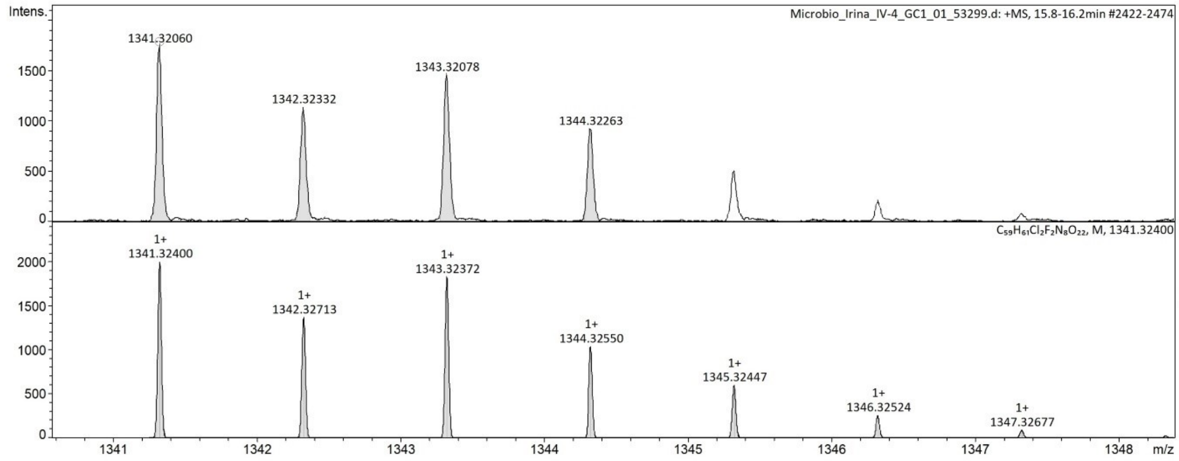

Microbio_Irina_IV-4_GC1_01_53299.d
Bruker Compass DataAnalysis 4.4 printed: 5/11/2023 3:07:59 PM by: MS Page 1 of 1

Figure S45. The mass spectrum from HR HPLC-MS analysis of concentrated culture supernatant from *A. balhimycina* Δ*hpg* supplemented with 2-fluoro-DL-4-hydroxyphenylglycine. The mass indicated with the red arrow for *m/z* 1341.3240 $[M+H]^+$ corresponds to a tricyclic balhimycin-type heptapeptide containing two fluorine atoms with a D-glucose moiety and an N-terminal methyl group with molecular formula $C_{59}H_{61}Cl_2F_2N_8O_{22}$.

Mass Spectrum SmartFormula Report

**Analysis Info**		Acquisition Date	10/19/2021 10:55:43 PM	
Analysis Name	F:\Daten\Maxis_2021\Stegmann\Irina\Irina_211020\Microbio_Irina_IV-4_GC1_01_53299.d			
Method	automsms_50-1800_pos_spez.fraglist_xx_0.3.m	Operator	BDAL@DE	
Sample Name	Microbio_Irina_IV-4	Instrument	maXis	288882.21253
Comment				

Acquisition Parameter

Source Type	ESI	Ion Polarity	Positive	Set Nebulizer	2.0 Bar
Focus	Not active	Set Capillary	4500 V	Set Dry Heater	200 °C
Scan Begin	50 m/z	Set End Plate Offset	-500 V	Set Dry Gas	8.0 l/min
Scan End	1800 m/z	Set Charging Voltage	0 V	Set Divert Valve	Waste
		Set Corona	0 nA	Set APCI Heater	0 °C

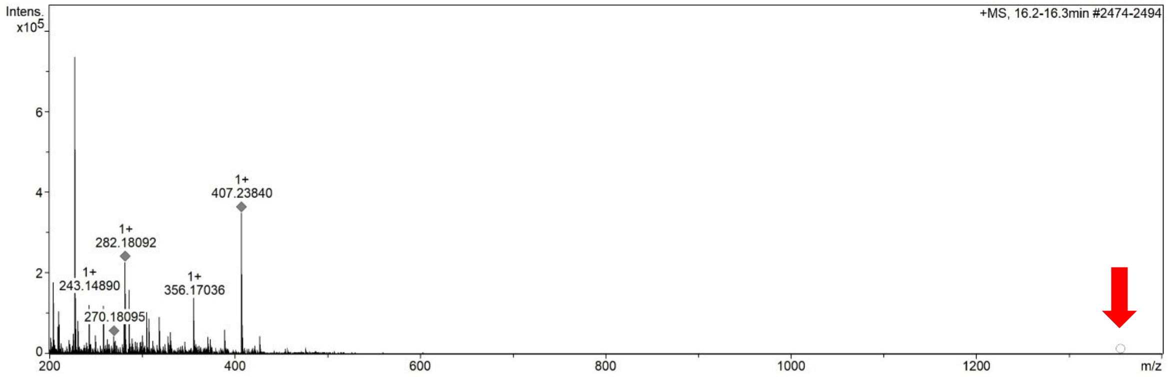

Meas. m/z	#	Ion Formula	m/z	err [ppm]	Mean err [ppm]	rdb	N-Rule	e⁻ Conf	mSigma	Std I	Std Mean m/z	Std I VarNorm	Std m/z Diff	Std Comb Dev
1355.33601	1	C60H63Cl2F2N8O22	1355.33965	2.69	1.47	32.0	ok	even	240.9	239.1	n.a.	n.a.	n.a.	n.a.

Microbio_Irina_IV-4_GC1_01_53299.d
Bruker Compass DataAnalysis 4.4 printed: 5/11/2023 3:15:13 PM by: MS Page 1 of 1

Display Report

**Analysis Info**		Acquisition Date	10/19/2021 10:55:43 PM	
Analysis Name	F:\Daten\Maxis_2021\Stegmann\Irina\Irina_211020\Microbio_Irina_IV-4_GC1_01_53299.d			
Method	automsms_50-1800_pos_spez.fraglist_xx_0.3.m	Operator	BDAL@DE	
Sample Name	Microbio_Irina_IV-4	Instrument	maXis	288882.21253
Comment				

Acquisition Parameter

Source Type	ESI	Ion Polarity	Positive	Set Nebulizer	2.0 Bar
Focus	Not active	Set Capillary	4500 V	Set Dry Heater	200 °C
Scan Begin	50 m/z	Set End Plate Offset	-500 V	Set Dry Gas	8.0 l/min
Scan End	1800 m/z	Set Charging Voltage	0 V	Set Divert Valve	Waste
		Set Corona	0 nA	Set APCI Heater	0 °C

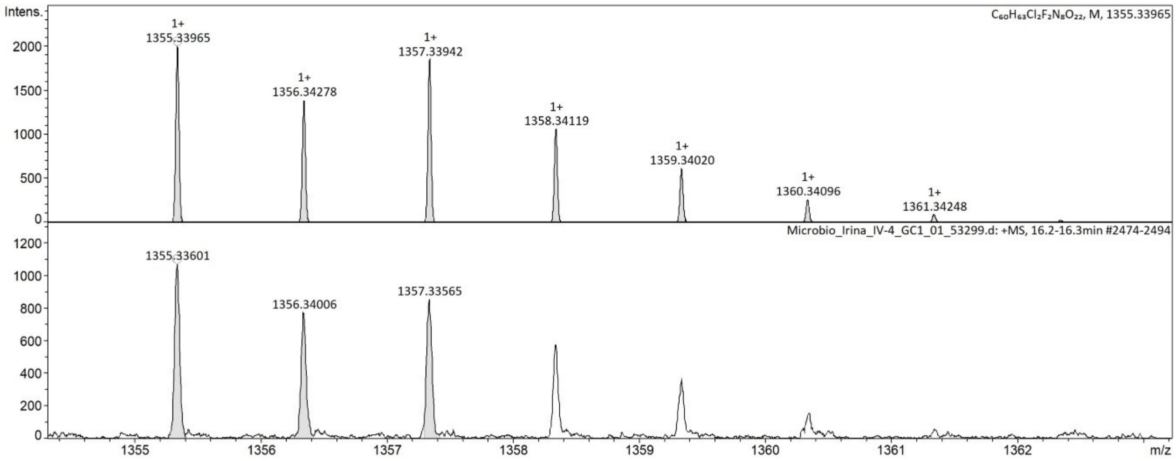

Microbio_Irina_IV-4_GC1_01_53299.d
Bruker Compass DataAnalysis 4.4 printed: 5/11/2023 3:14:54 PM by: MS Page 1 of 1

Figure S46. The mass spectrum from HR LC-MS analysis of concentrated culture supernatant from *A. balhimycina* Δ*hpg* supplemented with 2-fluoro-DL-4-hydroxyphenylglycine. The mass indicated with the red arrow for *m/z* 1355.3396 $[M+H]^+$ corresponds to a tricyclic balhimycin-type heptapeptide

containing two fluorine atoms with a D-glucose moiety and two N-terminal methyl groups with molecular formula $C_{60}H_{63}Cl_2F_2N_8O_{22}$.

Mass Spectrum SmartFormula Report

Analysis Info

Analysis Name	F:\Daten\Maxis_2021\Stegmann\Irina\Irina_211020\Microbio_Irina_IV-4_GC1_01_53299.d	Acquisition Date	10/19/2021 10:55:43 PM
Method	automsms_50-1800_pos_spez.fraglist_xx_0.3.m	Operator	BDAL@DE
Sample Name	Microbio_Irina_IV-4	Instrument	maXis 288882.21253
Comment			

Acquisition Parameter

Source Type	ESI	Ion Polarity	Positive	Set Nebulizer	2.0 Bar
Focus	Not active	Set Capillary	4500 V	Set Dry Heater	200 °C
Scan Begin	50 m/z	Set End Plate Offset	-500 V	Set Dry Gas	8.0 l/min
Scan End	1800 m/z	Set Charging Voltage	0 V	Set Divert Valve	Waste
		Set Corona	0 nA	Set APCI Heater	0 °C

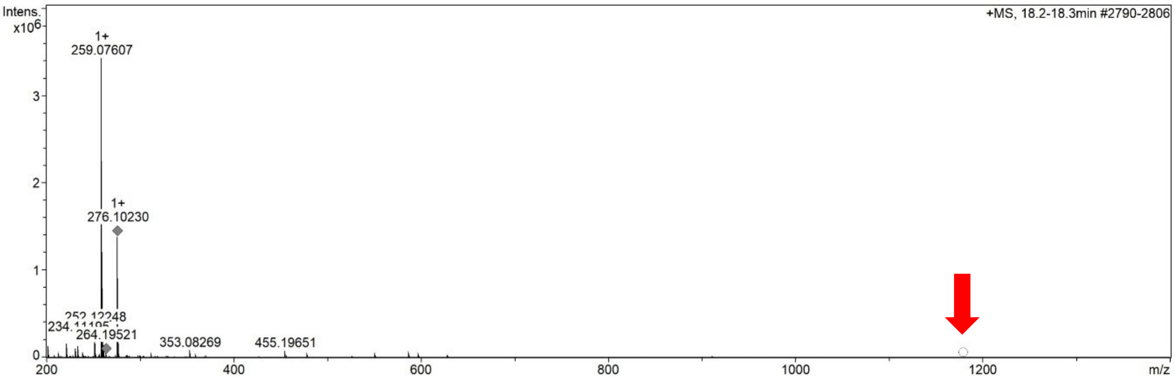

Meas. m/z	#	Ion Formula	m/z	err [ppm]	Mean err [ppm]	rdb	N-Rule	e⁻ Conf	mSigma	Std I	Std Mean m/z	Std I VarNorm	Std m/z Diff	Std Comb Dev
1179.26831	1	C53H51Cl2F2N8O17	1179.27118	2.43	0.92	31.0	ok	even	307.6	395.3	n.a.	n.a.	n.a.	n.a.

Microbio_Irina_IV-4_GC1_01_53299.d
Bruker Compass DataAnalysis 4.4 printed: 5/11/2023 2:51:24 PM by: MS Page 1 of 1

Display Report

Analysis Info

Analysis Name	F:\Daten\Maxis_2021\Stegmann\Irina\Irina_211020\Microbio_Irina_IV-4_GC1_01_53299.d	Acquisition Date	10/19/2021 10:55:43 PM
Method	automsms_50-1800_pos_spez.fraglist_xx_0.3.m	Operator	BDAL@DE
Sample Name	Microbio_Irina_IV-4	Instrument	maXis 288882.21253
Comment			

Acquisition Parameter

Source Type	ESI	Ion Polarity	Positive	Set Nebulizer	2.0 Bar
Focus	Not active	Set Capillary	4500 V	Set Dry Heater	200 °C
Scan Begin	50 m/z	Set End Plate Offset	-500 V	Set Dry Gas	8.0 l/min
Scan End	1800 m/z	Set Charging Voltage	0 V	Set Divert Valve	Waste
		Set Corona	0 nA	Set APCI Heater	0 °C

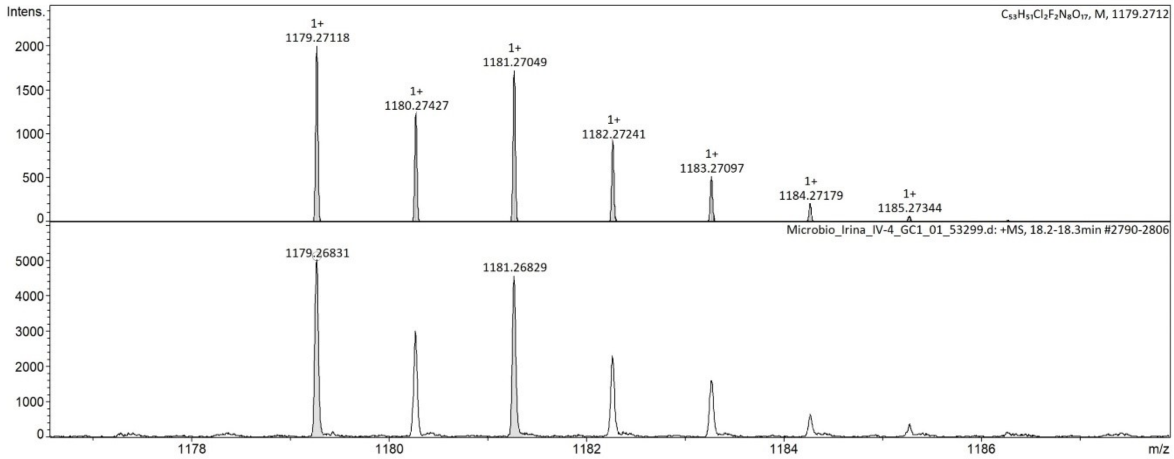

Microbio_Irina_IV-4_GC1_01_53299.d
Bruker Compass DataAnalysis 4.4 printed: 5/11/2023 2:52:53 PM by: MS Page 1 of 1

Figure S47. The mass spectrum from HR HPLC-MS analysis of concentrated culture supernatant from *A. balhimycina* Δ*hpg* supplemented with 2-fluoro-DL-4-hydroxyphenylglycine. The mass indicated with the red arrow for *m/z* 1179.2711 $[M+H]^+$ corresponds to a tricyclic balhimycin-type heptapeptide

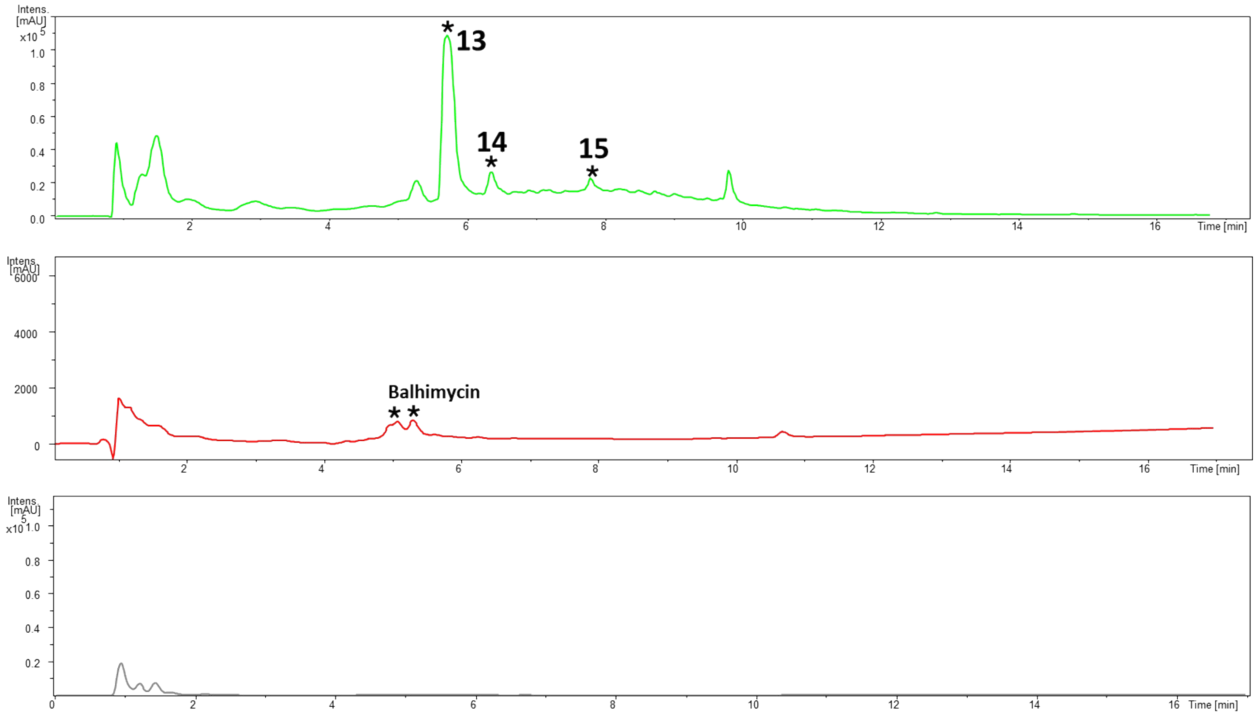

containing two fluorine atoms with molecular formula $C_{53}H_{51}Cl_2F_2N_8O_{17}$.

Figure S48 HPLC-DAD chromatograms of concentrated culture supernatant after cultivation in R5 medium. Green line - from *A. balhimycina* Δ*hpg* supplemented with 2-fluoro-DL-4-hydroxyphenylglycine; red line - from *A. balhimycina* WT; grey line - *A. balhimycina* Δ*hpg* negative control. The asterisk (*) sign indicates the peaks where the balhimycin molecule and novel derivatives (13,14,15) were detected.

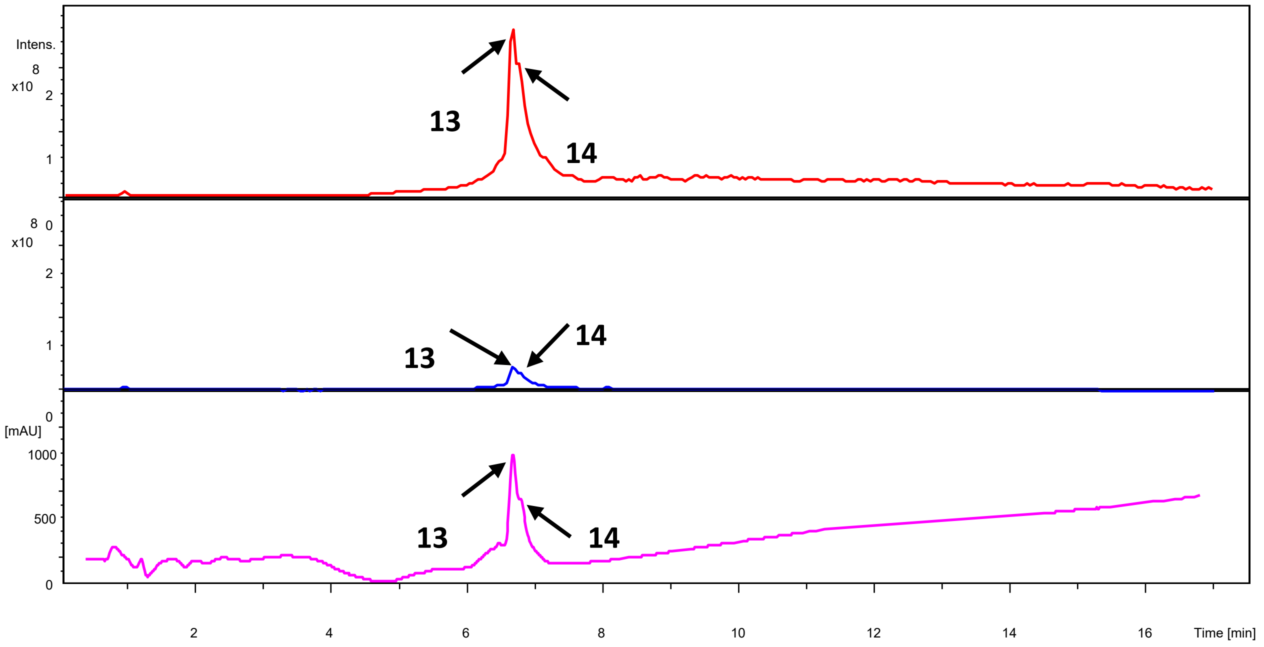

Figure S49 HPLC-MS chromatograms of purified balhimycin derivatives 13/14 from *A. balhimycina* Δ*hpg* supplemented with 2-fluoro-DL-4-hydroxyphenylglycine (4-F-Hpg). Red line – TIC in positive ion mode, blue line – TIC in negative ion mode, purple line – UV chromatogram (200-600 nm). The arrow indicates balhimycin derivatives 13/14 eluted as a mixture.

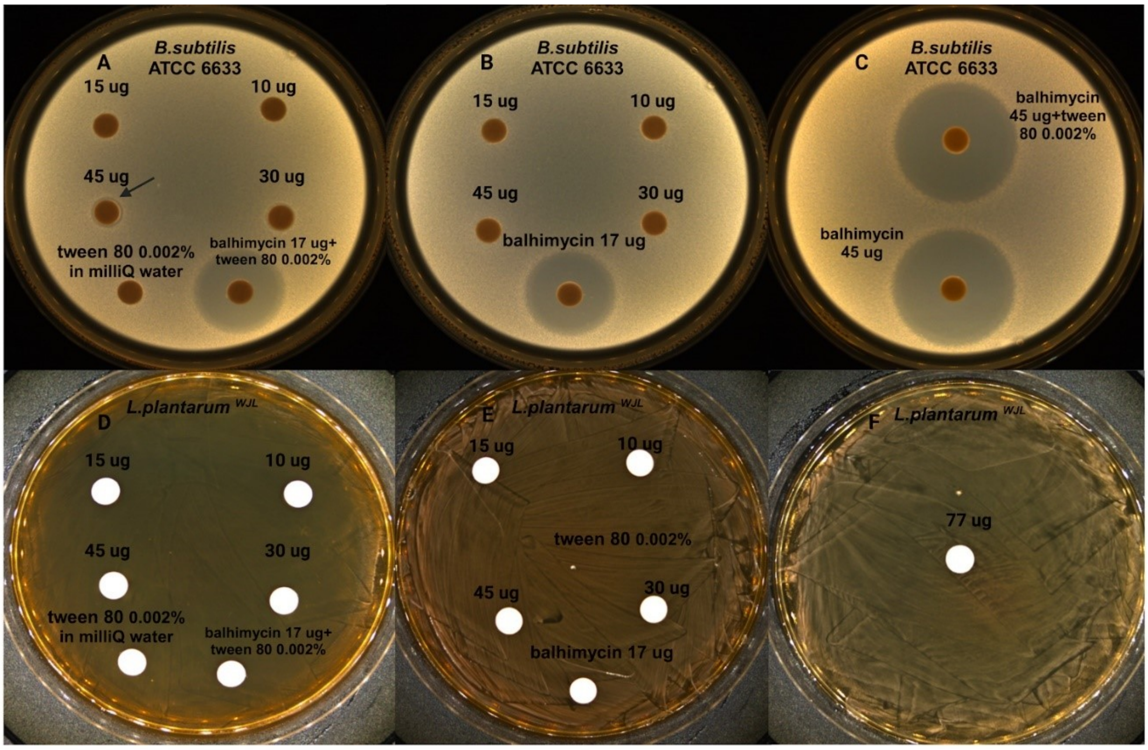

Figure S50. MIC assays of GPA compounds. (A). Bioactivity assays against *B. subtilis* ATCC 6633 using **13/14** GPAs and balhimycin dissolved in Milli-Q water with the addition of 0.002% polysorbate Tween 80 in Milli-Q water and tested at different amounts: 10 µg, 15 µg, 30 µg, and 45 µg for **13/14** and 17 µg for balhimycin. The arrow indicates the zone of inhibition (7 mm) for **13/14** GPAs, whereas 17 µg of balhimycin resulted in a zone of inhibition of 19 mm. (B). Bioactivity assays against *B. subtilis* ATCC 6633 using **13/14** GPAs and balhimycin, dissolved in Milli-Q water and tested at different amounts: 10 µg, 15 µg, 30 µg, and 45 µg for **13/14** and 17 µg for balhimycin, resulting in only an inhibition zone (20 mm) for balhimycin. (C). Bioactivity assays against *B. subtilis* ATCC 6633; balhimycin was dissolved in 0.002% polysorbate Tween 80 in Milli-Q water. 45 µg of both samples were tested, resulting in inhibition zones of 26 mm and 27 mm, respectively. (D). Bioactivity assays against *L. plantarum* ^{WJL} using **13/14** GPAs and balhimycin dissolved in 0.002% polysorbate Tween 80 in Milli-Q and tested at different amounts: 10 µg, 15 µg, 30 µg, and 45 µg for **13/14** and 17 µg for balhimycin. (E). Bioactivity assays against *L. plantarum* ^{WJL} using **13/14** GPAs and balhimycin dissolved in Milli-Q water and tested at different amounts: 10 µg, 15 µg, 30 µg, and 45 µg for **13/14** and 17 µg for balhimycin. (F). Bioactivity assays against *L. plantarum* ^{WJL} using **13/14** GPAs dissolved in Milli-Q water and tested at 77 µg.

*Diameter of disks – 6 mm.

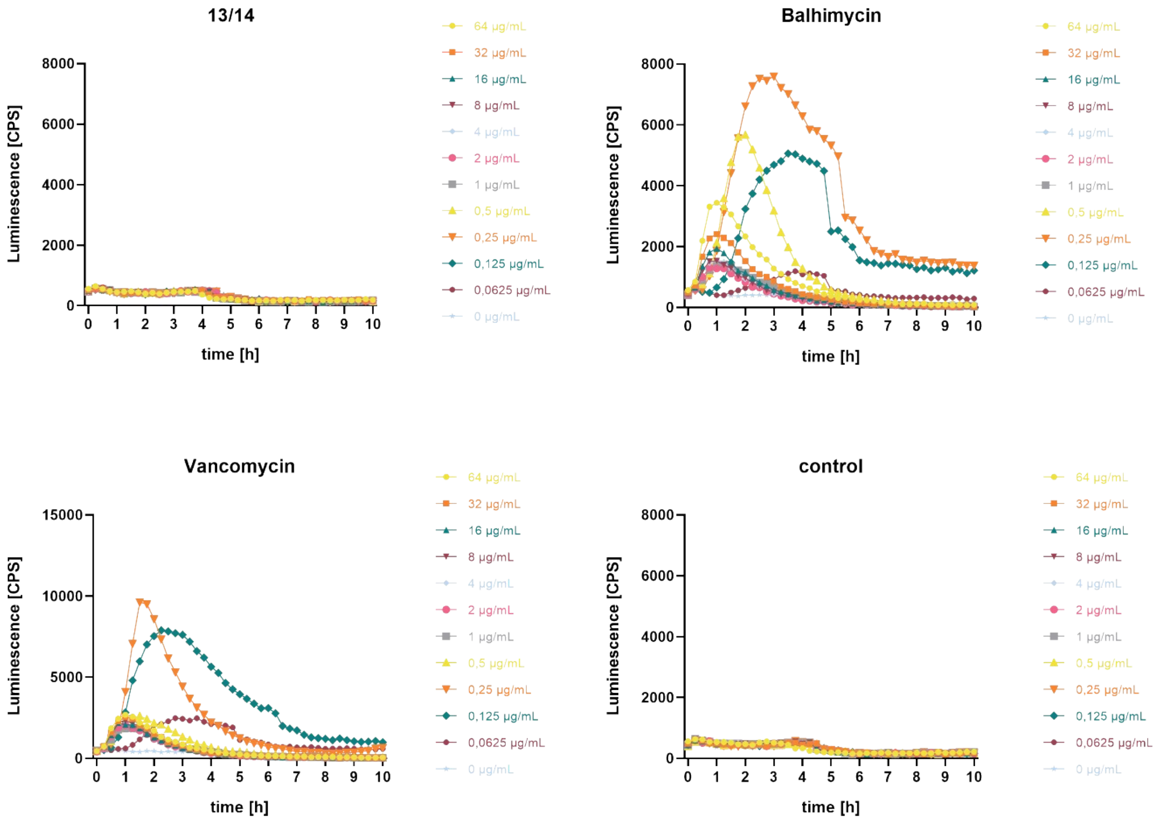

Figure S51. The LiaRS stress response is strongly induced by the GPAs balhimycin and vancomycin, but not by 13/14. The expression of bioluminescence in *B. subtilis* W168 sacA:pCHlux101 (P_{liaI}-*lux*) was monitored after addition of the GPAs or in untreated cells as a control. Balhimycin and vancomycin induce the LiaRS stress response in a concentration-dependent manner, while no luminescence could be detected after addition of **13/14** or in the control.

Supplementary Protocols

Protocol S1: Synthesis of 2-F-4-Hpg.

4-(Benzyloxy)-2-fluorobenzaldehyde (**17**). To suspension of 2-fluorobenzaldehyde[xx] (4.90 g, 35.0 mmol) and potassium carbonate (5.80 g, 42.0 mmol) in dry DMF (105 mL) was slowly added benzyl bromide (7.18 g, 5 mL, 42.0 mmol) dropwise at room temperature. After being stirred for 72 h at room temperature, the mixture containing a white precipitate, was poured on ice water (100 mL). The precipitate was filtered off, washed with cold water (200 mL) and then dissolved in ethyl acetate (300 mL). The ethyl acetate layer was washed with saturated NaCl solution (100 mL), dried over $MgSO_4$, filtered and concentrated in vacuo. The aldehyde **17** (7.80 g, 33.9 mmol, 97%) was obtained as a white powder. R_f (petroleum ether/Et_2O, 2:1): 0.5; ^1H NMR (400 MHz, $CDCl_3$): δ = 5.11 (s, 2H), 6.70 (dd, *J* = 12.3, 2.3 Hz, 1H), 6.82–6.86 (m, 1H), 7.24–7.42(m, 5H), 7.81 (t, *J* = 8.9 Hz, 1H), 10.19(s, 1H).

2-Amino-2-(4-(benzyloxy)-2-fluorophenyl)acetonitrile (**18**). Ammonium chloride (499 mg, 9.33 mmol) was added to a solution of sodium cyanide (305 mg, 6.2 mmol) in 25% ammonia (12.8 mL) and the resulting reaction mixture was cooled to 0 °C. A solution of 4-benzyloxy-2-fluorobenzaldehyde (895 mg, 3.89 mmol) in MeOH (37 mL) was added, before the mixture was stirred for 17 h at room temperature. Most of the organic solvent was removed on the rotary evaporator and the residue diluted with water (20 mL) and extracted with EtOAc (3 × 20 mL). The combined layers were washed with saturated aqueous $NaHCO_3$ solution (2 × 30 mL) and saturated NaCl solution (40 mL), dried over $MgSO_4$, filtered and concentrated in vacuo to afford the title compound as a viscous oil (918 mg, 92%): ^1H NMR (400 MHz, $CDCl_3$): δ = 5.01–5.07 (m, 1H), 5.08 (s, 2H), 6.73–6.79 (m, 1H), 6.80–6.85 (m, 1H), 7.33-7.45 (m, 6H).

Amino-(2-fluoro-4-hydroxyphenyl)acetic acid hydrochloride (**3**). A suspension of amino-(4-benzyloxy-2-fluorophenyl)acetonitrile (**18**) (1.22 g, 8.90 mmol) in HCl (6M, 20 mL) was stirred at 100 °C for 5 h. The solvent was removed in vacuo and the remainder was triturated with Et_2O (2 × 6 mL) to afford the amino acid hydrochloride **3** (1.04 g, 98%, crude) as a brown powder: ^1H NMR (400 MHz, CD_3OD): δ = 5.18 (s, 1H), 6.62–6.67 (m, 1H), 6.68–6.73 (m, 1H), 7.25–7.31 (m, 1H), ^{13}C NMR (100 MHz, CD_3OD): δ = 64.9 (C2), 104.1, 104.4 (C3'), 111.7 (C5'), 113.5 (C1'), 131.8 (C6'), 150.2 (C4'), 161.8, 162.4 (C2'), 170.7 (C1).

Protocol S2: Fermentation and purification of fluoro-balhimycin.

For purification of novel fluorinated balhimycin derivatives, the mutant *A. balhimycina* Δ*hpg* was cultivated in 10 L of R5 medium as described in methods part with supplementation of the mutasynthon (0.015 mg/mL). To eliminate the residual metabolites, the supernatant from the 10 L culture was mixed and extracted by liquid-liquid extraction with ethyl acetate (1:1 volume) and 1-butanol (1:1 volume). After solvent pre-extraction, the supernatant was evaporated in vacuo to remove remaining organic solvents, loaded on a glass column (8.5 cm in diameter, 54 cm in length) packed with Diaion HP-20 adsorbent resin and washed first with water and then with different water-methanol mixtures (10%, 20%, 40%, 60%, 80% and 100% methanol). Targeted fluorinated balhimycin derivatives were detected in 10% -20% methanol fractions by HPLC-MS analysis and evaporated for size-exclusion chromatography. Crude extract was dissolved in 20 mL of 100% methanol and loaded on a glass column (4,5 cm in diameter, 78 cm in length) packed with Sephadex® LH 20 (Sigma-Aldrich, USA). Methanol was used as eluent and fractions were collected every 20 min with a speed of 1 drop per three seconds. Obtained fractions were analyzed with HPLC-MS. Further purification was performed on a preparative HPLC system equipped with a Reprosil – 100 C18 column (7 µM, 250*20 mm, Dr. Maisch, Ammerbuch-Entringen, Germany) with initial elution 2–98% solvent B (acetonitrile) against solvent A (water + 0.1 % formic acid) over 30 min with UV absorption detection of 260 and 280 nm, respectively. Flow rate was of 24 mL/min. Targeted compounds were eluted with 33-34% of solvent B which resulted in 240 mg of semi-pure material. The obtained fractions were combined and dissolved in 10% DMSO in water and purified using a Reprosil pur Basic C18 column (10 µM, 250*20 mm, Dr. Maisch, Ammerbuch-Entringen, Germany) with the same solvent system. The purification method started with 98% of solvent A (water + 0.1 % formic acid) against solvent B (acetonitrile) for 2 minutes and then decreased to 90% for 8 minutes and then went down to 80 % for 16 minutes. The targeted compounds were eluted with 17-18% of solvent B with UV absorption at 260 and 280 nm, respectively. 46 mg of semi-pure material was obtained for size-exclusion chromatography using SkillPak50 TOYOPEARL HW-40F column (2.5 cm diameter, 10 cm length, TOSOH, Canada) with a flow rate of 20 mL/min and milliQ water as eluent. Fractions were collected for 5 minutes, and analysed by LC-MS. 21.3 mg of material was subjected to preparative HPLC system with a flow rate of 24 mL/min equipped with a Reprosil – 100 C18 column (7 µM, 250*20 mm, Dr. Maisch, Ammerbuch-Entringen, Germany) with initial elution 2–98% solvent B (acetonitrile) against solvent A (water + 0.1 % formic acid) over 20 minutes with UV detection at 260 and 280 nm, respectively. The targeted compounds were eluted with 12-14% of solvent B, checked via HPLC-MS, combined and yielded to 3 mg of pure substance.

Supplementary References

1 Nadkarni, S. R. *et al.* Balhimycin, a new glycopeptide antibiotic produced by Amycolatopsis sp. Y-86,21022. Taxonomy, production, isolation and biological activity. *J Antibiot (Tokyo)* **47**, 334-341, (1994).

2 Bierman, M. *et al.* Plasmid cloning vectors for the conjugal transfer of DNA from Escherichia coli to Streptomyces spp. *Gene* **116**, 43-49, (1992).

3 Pelzer, S. *et al.* Identification and Analysis of the Balhimycin Biosynthetic Gene Cluster and Its Use for Manipulating Glycopeptide Biosynthesis in *Amycolatopsis mediterranei* DSM5908. *Antimicrob Agents Chemother* **43**, 1565-1573, (1999).

4 Siezen, R. J. *et al.* Phenotypic and genomic diversity of Lactobacillus plantarum strains isolated from various environmental niches. *Environ Microbiol* **12**, 758-773, (2010).

5 Dietrich, A., Steffens, U., Sass, P. & Bierbaum, G. The hypersusceptible antibiotic screening strain Staphylococcus aureus SG511-Berlin harbors multiple mutations in regulatory genes. *International Journal of Medical Microbiology* **311**, 151545, (2021).

6 Radeck, J. *et al.* Anatomy of the bacitracin resistance network in Bacillus subtilis. *Molecular Microbiology* **100**, 607-620, (2016).

7 Pelzer, S., Reichert, W., Huppert, M., Heckmann, D. & Wohlleben, W. Cloning and analysis of a peptide synthetase gene of the balhimycin producer Amycolatopsis mediterranei DSM5908 and development of a gene disruption/replacement system. *J. Biotech.* **56**, 115-128, (1997).

8 Stachelhaus, T., Mootz, H. D. & Marahiel, M. A. The specificity-conferring code of adenylation domains in nonribosomal peptide synthetases. *Chem. Biol.* **6**, 493-505, (1999).

9 Hansen, M. H. *et al.* Resurrecting ancestral antibiotics: unveiling the origins of modern lipid II targeting glycopeptides. *Nat. Commun.* **14**, 7842, (2023).

10 Ho, Y. T. C. *et al.* Exploring the Flexibility of the Glycopeptide Antibiotic Crosslinking Cascade for Extended Peptide Backbones. *ChemBioChem* **24**, e202200686, (2023).
